# Cytotoxic Indole-Diterpenoids from the Marine-Derived Fungus *Penicillium* sp. KFD28

**DOI:** 10.3390/md19110613

**Published:** 2021-10-28

**Authors:** Lu-Ting Dai, Li Yang, Fan-Dong Kong, Qing-Yun Ma, Qing-Yi Xie, Hao-Fu Dai, Zhi-Fang Yu, You-Xing Zhao

**Affiliations:** 1 College of Food Science and Technology, Nanjing Agricultural University, Nanjing 210095, China; dailuting121@163.com; 2 Haikou Key Laboratory for Research and Utilization of Tropical Natural Products, Institute of Tropical Bioscience and Biotechnology, CATAS, Haikou 571101, China; yangli@itbb.org.cn (L.Y.); maqingyun@itbb.org.cn (Q.-Y.M.); xieqingyi@itbb.org.cn (Q.-Y.X.); 3 Key Laboratory of Chemistry and Engineering of Forest Products, State Ethnic Affairs Commission, Guangxi Key Laboratory of Chemistry and Engineering of Forest Products, Guangxi Collaborative Innovation Center for Chemistry and Engineering of Forest Products, School of Chemistry and Chemical Engineering, Guangxi University for Nationalities, Nanning 530006, China; kongfandong501@126.com; 4 Hainan Institute for Tropical Agricultural Resources, CATAS, Haikou 571101, China; daihaofu@itbb.org.cn

**Keywords:** marine-derived fungus, *Penicillium* sp., indole-diterpenoids, cytotoxicity, antibacterial activity

## Abstract

Four new indole-diterpenoids, named penerpenes K-N (**1**–**4**), along with twelve known ones (**5**–**16**), were isolated from the fermentation broth produced by adding L-tryptophan to the culture medium of the marine-derived fungus *Penicillium* sp. KFD28. The structures of the new compounds were elucidated extensively by 1D and 2D NMR, HRESIMS data spectroscopic analyses and ECD calculations. Compound **4** represents the second example of paxilline-type indole diterpene bearing a 1,3-dioxepane ring. Three compounds (**4**, **9,** and **15**) were cytotoxic to cancer cell lines, of which compound **9** was the most active and showed cytotoxic activity against the human liver cancer cell line BeL-7402 with an IC_50_ value of 5.3 μM. Moreover, six compounds (**5**, **7**, **10**, **12**, **14**, and **15**) showed antibacterial activities against *Staphylococcus aureus* ATCC 6538 and *Bacillus subtilis* ATCC 6633.

## 1. Introduction

Marine fungi have formed different metabolic pathways and adaptation mechanisms within the peculiar marine environment. Hence, marine fungi can produce natural secondary metabolites that are characterized by unique chemical structures and high biological activities [1,2]. Alkaloids derived from marine-derived compounds have received extensive attention in recent years. Indole alkaloids, as an important class of secondary metabolites produced by marine-derived fungi [3], showed excellent biological activities, including cytotoxic [4], antibacterial [5], quorum sensing inhibitory [6], anti-Zika virus [7], and protein tyrosine phosphatase inhibitory activities [8,9].

**Figure 1:**
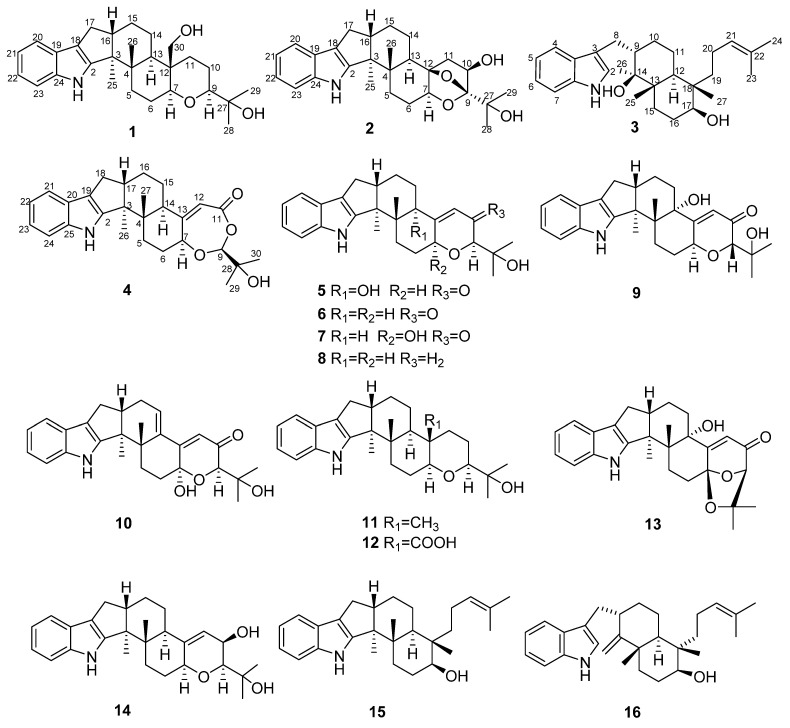
The chemical structures of compounds **1**–**16**.

Marine-derived fungus *Penicillium* sp. KFD28 was isolated and identified from bivalve shellfish, *Meretrix lusoria*, collected from Haikou Bay, China. Our previous study on the secondary metabolites of this fungus discovered a series of indole alkaloids with novel structures and intriguing bioactivities, e.g., protein tyrosine phosphatase inhibitory activity [8,9]. The OSMAC (one strain, many compounds) approach is highly efficient for inducing structural diversity by the variation of cultivation conditions [10]. Most of the indole alkaloids precursors are related to L-tryptophan [11]. To find more new alkaloids from marine fungi, adding amino acids to the culture media is becoming a viable strategy [12,13]. In order to explore the metabolic potential of the fungus *Penicillium* sp. KFD28, we recultured this fungus by adding L-tryptophan to the solid rice culture and found that the HPLC profiles of the extract are different from that of the first time obtained from the liquid medium. Later chemical investigation of the fermentation broth led to the isolation of four new indole-diterpenoids, penpenes K-N (**1**–**4**), along with 12 known ones, including paxilline (**5**) [14], dehydroxypaxilline (**6**) [15], 7-hydroxyl-13-dehydroxypaxilline (**7**) [16], 3-deoxo-4b-deoxypaxilline (**8**) [17], epipaxilline (**9**) [18], 7-hydroxypaxilline-13-ene (**10**) [19], paspaline (**11**) [20], 4a-demethylpaspaline-4a-carboxylic acid (**12**) [17], paspalinine (**13**) [21], PC-M6 (**14**) [22], emindole SB (**15**) [23], emeniveol (**16**) [24] (Figure 1). Herein, the isolation, structure elucidation, and bioactivities of these compounds are reported.

## 2. Results and Discussions

### 2.1. Structure Elucidation

**Figure 2:**
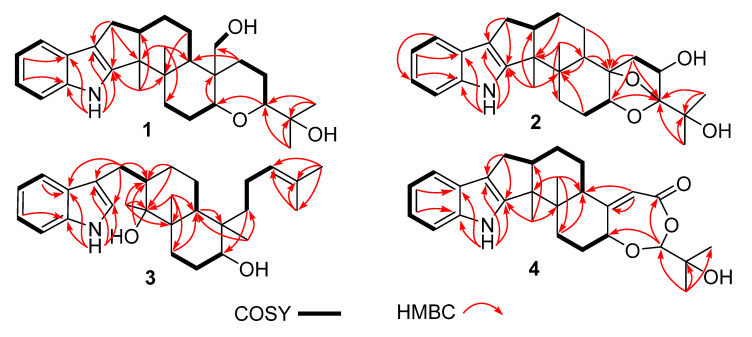
Key COSY and HMBC correlations of compounds **1**–**4**.

**Figure 3:**
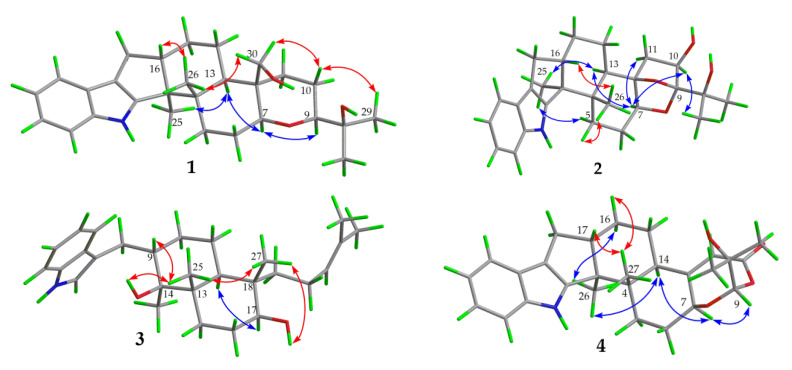
Key ROESY correlations of compounds **1**–**4**.

**Figure 4:**
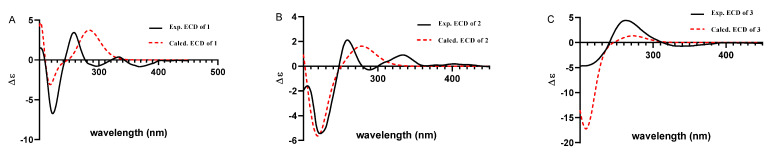
Experimental and calculated ECD curves for compound **1** (**A**); compound **2** (**B**); compound **3** (**C**).

Compound **1** was obtained as a yellow oil. Its formula was determined as C_28_H_39_NO_3_ on the basis of HRESIMS data (*m*/*z* 476.2565 for [M+K]^+^), indicating ten degrees of unsaturation. The ^1^H and ^13^C NMR data of **1**, with the aid of its HSQC spectrum (Supplementary Materials, Figures S1–S4), showed a total of 28 carbon signals comprising eight olefinic or aromatic carbons (four protonated) for a 2,3-disubstituted indole moiety, four methyls, eight sp^3^ methylenes with one oxygenated, four sp^3^ methines with two oxygenated, and four non-protonated sp^3^ carbons with one oxygenated. These NMR data allowed the construction of the carbon skeleton of indole-diterpenoid. Careful contrast of the similar NMR spectral data between **1** and paspaline (**11**) [20] revealed that they had the same planar structure except that the methyl group at C-12 (*δ*_C_ 36.6) in paspaline was oxidized to oxymethylene in **1**. Consistent with the introduction of the hydroxyl at C-30 (*δ*_C_ 58.5), it showed a distinctive deshielding of the C-12 signal (*δ*_C_ 48.8) in **1** compared to those of paspaline (*δ*_C_ 36.6). The linkage of a hydroxyl at C-30 was further supported by the COSY correlation of 30-OH/H_2_-30 (Figure 2; Supplementary Material, Figure S8) and ROESY correlation of H_3_-26/H_2_-30 (Supplementary Material, Figure S9). Detailed analysis of 2D NMR data further confirmed that **1** and paspaline share the same indole-diterpenoid skeleton. The relative configuration of 6/6/6 tricyclic rings in indole-diterpenoid were determined by the ROESY spectrum (Figure 3), in which the sequential correlations of H-16/H_3_-26/H_2_-30/H-10*β*/H_3_-28 suggested the same face of H-16, CH_3_-26, CH_2_-30, and C-27 in the 6/6/6 tricyclic ring system, while the correlations of H_3_-25/H-13/H-7/H-9 suggested CH_3_-25, H-13, H-7, and H-9 were on the opposite face of this system. It has been reported that the strong Cotton effect (CE) around 220 nm was related to the absolute configurations of the chiral carbons around the indole chromophore in the paxilline-type indole-diterpene [17]. Thus, the strong negative CE at 223 nm in the experimental ECD spectrum of **1** (Figure 4) suggested its (3*S*,4*S*,7*S*,9*S*,12*S*,13*R*,16*S*)-**1** absolute configuration [17]. As for the absolute configuration of **1**, the ECD spectrum of (3*S*,4*S*,7*S*,9*S*,12*S*,13*R*,16*S*)-**1** was calculated; this deduction was further supported, establishing the absolute configuration of **1** as presented in Figure 1.

Compound **2** was isolated as a yellow oil. Its formula was determined as C_27_H_35_NO_4_ on the basis of HRESIMS data, indicating 11 degrees of unsaturation. Its ^13^C NMR data showed a total of 27 carbon signals comprising eight aromatic or olefinic carbons for an indole moiety, four methyls, six sp^3^ methylenes, four sp^3^ methines with two oxygenated, and five non-protonated sp^3^ carbons with three oxygenated. Analysis of the NMR spectra (Table 1 and Table 2, Supplementary Materials, Figures S12–S18) of **2** suggested that its structure was related to that of 4a-demethylpaspaline-3,4,4a-triol [17], and the main difference being C-9 (*δ*_C_ 109.8), C-11 (*δ*_C_ 43.7), and C-12 (*δ*_C_ 84.6) in **2** instead of C-9 (*δ*_C_ 82.6), C-11 (*δ*_C_ 73.5), and C-12 (*δ*_C_ 76.1) in 4a-demethylpaspaline-3,4,4a-triol, indicating the replacement of two oxygenated carbons by a methylene (C-11) and an acetal carbon (C-9) in **2**. The HMBC correlations (Supplementary Material, Figure S16) from H-11, H-7, H-29, and H-28 to C-9 confirmed this deduction. The presence of an indole moiety, together with the HRESIMS data (Supplementary Material, Figure S19), indicated that **2** has a heptacyclic ring system. At this point, one more ring was required to fulfill the 11 double-bond equivalents, and an oxygen bridge was proposed according to the molecular formula. The oxygen bridge was assigned to connect C-9 and C-12 as deduced from distinctive deshielding of the C-12 (*δ*_C_ 84.6) and C-9 (*δ*_C_ 109.8) in **2** compared to corresponding C-12 (*δ*_C_ 76.1) and C-9(*δ*_C_ 96.4-96.5) [25,26] with a free hydroxyl group. In the ROESY spectrum (Supplementary Material, Figure S18), correlations of H-16/H_3_-26/H-5*β*, and H-5*α*/H_3_-25/H-13/H-7/H-10/H_3_-28 determined the relative configuration of **2**, as shown in Figure 3. In addition, the *β* orientation of the oxygen bridge was also proved by the ROESY correlation of H-7/H_2_-11 with the aid of the 3D ball-and-stick molecular model. Thus, the planar structure of compound **2** was established and named penerpene L. The ECD curve (Figure 4) of compound **2** is similar to **1**, indicating that the absolute configurations for the chiral carbons C-3, C-4, C-16, and C-13 in **1** were the same as those of **2**. The ECD calculation experiment also confirmed the above deduction (Figure 4), establishing the (3*S*,4*S*,7*S*,9*S*,10*R*,12*R*,13*R*,16*S*)-**2** absolute configuration.

Compound **3** was assigned the molecular formula of C_28_H_41_NO_2_ by HRESIMS, indicating nine degrees of unsaturation. The ^1^H NMR spectrum (Supplementary Material, Figure S21) displayed the typical pattern of a 3-substituted indole moiety with five aromatic protons at *δ*_H_ 7.04 (s, H-2), 7.30 (d, *J* = 8.0, H-7), 6.93 (t, *J* = 7.6, H-5), 7.02 (t, *J* = 7.6, H-6) and 7.55 (d, *J* = 8.0, H-4), as well as one clear olefinic proton at *δ*_H_ 5.05 (t, H-21), one oxygenated proton *δ*_H_ 3.27 (1H, H-17), and five methyls at *δ*_H_ 1.62 (s, 24-Me), 1.55 (s, 23-Me), 0.62 (s, 27-Me), 1.11 (s, 26-Me), 0.94 (s, 25-Me). The ^13^C NMR data of **3** (Supplementary Materials, Figures S22 and S23) displayed 28 carbon signals, including ten aromatic or olefinic carbons (six protonated), five methyls, seven sp^3^ methylenes, three sp^3^ methines with one oxygenated, three non-protonated sp^3^ carbons with one oxygenated. These NMR spectra data indicated that **3** was very similar to emeniveol (**16**) [24] except that a methyl in C-26 (*δ*_C/H_ 16.3/1.11) and an oxygenated non-protonated sp^3^ carbon C-14 (*δ*_C_ 76.4) in **3** replaced two olefinic carbons in emeniveol, suggesting that the exocyclic double bond in emeniveol changed to a methyl and an oxygenated non-protonated sp^3^ carbon. This obvious difference was supported by the HMBC correlations (Supplementary Material, Figure S25) from the H_3_-26 to C-14, C-13 (*δ*_C_ 42.0) and C-9 (*δ*_C_ 42.9). The relative configuration of **3** was determined by the ROESY spectrum (Figure 3), and the correlations of 14-OH/H_3_-25/H_3_-27/17-OH and H-9/H_3_-25 suggested the same face of these protons, while the correlation of H-12/H-17 indicated that these protons were on the opposite face to 17-OH. Thus, the planar structure of **3** was assigned, as shown in Figure 1. The absolute configuration of **3** was established as (9*S*,12*S*,13*S*,14*S*,17*S*,18*S*)-**3** by comparison of its experimental ECD spectrum with the calculated ECD curves (Figure 4).

The molecular formula of compound **4** was established as C_27_H_33_NO_4_ on the basis of HRESIMS data, indicating 12 degrees of unsaturation. Analysis of the ^1^H and ^13^C NMR spectra (Table 1 and Table 2, Supplementary Materials, Figures S30 and S31) of **4** suggested that its structure was closely related to that of penerpene G [9], a previously reported indole diterpene with an unusual 6/5/5/6/6/7 hexacyclic ring system bearing a 1,3-dioxepane ring. The main difference between them was the replacement of an oxygenated non-protonated sp^3^ carbon C-14 (*δ*_C_ 78.3) in penerpene G by that of a sp^3^ methine (*δ*_C_ 42.8) in **4**. This assignment was confirmed by HMBC (Figure 2) correlations from H-12 (*δ*_H_ 5.54) and H-27 (*δ*_H_ 0.82) to C-14 and the COSY correction of H-14/H-15. The ROESY corrections (Figure 3) of H_3_-27/H-17/H-16*β* and H-16*α/*H_3_-26/H-14/H-7/H-9 assigned the same relative configuration of **4** as that of penerpene G. The ECD curves of compound **4** (Supplementary Material, Figure S43) are similar to that reported for penerpene G, containing strong positive CEs at a shorter wavelength (219 nm in **4** and 220 nm in penerpene G) and strong negative CEs at a longer wavelength (238 nm in **4** and 234 nm in penerpene G), leading to the determination of the absolute configuration of **4** as (3*S*,4*S*,7*S*,9*S*,14*R*,17*S*)-**4**.

### 2.2. Biological Assay

The cytotoxic activities of compounds **1**–**16** were conducted by the MTT assay method [27] using cisplatin as the positive control. All compounds were tested against the human cervical cancer cell line HeLa, human gastric cancer cell line SGC-7901, human lung carcinoma cell line A549, and human liver cancer cell line BeL-7402. The results (Table 3) indicated that compound **9** exhibited the most pronounced activity against BeL-7402 with an IC_50_ value of 5.3 μM and was comparable to that of positive control cisplatin (IC_50_ 4.1 μM). Compound **9** also displayed moderate cytotoxic activity against A549. While compounds **4** showed low cytotoxicity against HeLa. Compound **15** displayed mild inhibitory activity against HeLa, A549, and BeL-7402 (IC_50_ = 24.4–40.6 μM). The remaining compounds were found to be inactive against the three cell lines. All the tested compounds **1**–**16** were inactive against the cell line SGC-7901. The loss of a hydroxyl at C-14 suggested being a determinant of cytotoxicity shown by compound **4** against HeLa (**4** vs. penerpene G [9]). It is worth mentioning that it was the first-time report of the cytotoxicity of epipaxilline (**9**) [18]. Arintari et al. [19] and Sallam et al. [28] demonstrated the cytotoxicities of emindole SB (**15**) against human breast cell line MCF-7, murine lymphoma cell line L5178Y, and the human embryonic kidney cell line HEK-293, which provides emindole SB (**15)** a meaningful pharmacophore for further biological studies.

Compounds **1**–**16** were also tested for their antibacterial activity against *Escherichia coli* ATCC 25922, *Staphylococcus aureus* ATCC 6538, *Listeria monocytogenes* ATCC 1911, and *Bacillus subtilis* ATCC 6633 using the 96-well microtiter plates method [29] reported previously and using ampicillin as a positive control. The results (Table 4) revealed that six compounds **5**, **7**, **10**, **12**, **14**, and **15** showed moderate inhibitory activity against *S. aureus* ATCC 6538. Emindole SB (**15**) displayed reported selectivity toward *S. aureus* ATCC 33591 (MIC = 6.25 μg/mL) [21]. Compound **7** showed reasonable antibacterial activity against *B. subtilis* ATCC 6633 (MIC = 16 μg/mL), but compounds **5**, **10**, and **12** exhibited lower inhibitory. The results of the rest ten compounds (**1**–**4**, **6**, **8**–**9**, **11**, **13**, and **16**) did not show remarkable antibacterial activities (MIC > 128 µg/mL) against *S. aureus* and *B. subtilis*. In this assay, none of these compounds showed inhibitory activity against *E. coli* ATCC 25922 and *L. monocytogenes* ATCC 1911 (MIC > 128 µg/mL).

## 3. Experimental

### 3.1. General Experimental Procedures

NMR spectra were recorded on Bruker AV-500 and Bruker AV-600 spectrometers (Bruker, Bremen, Germany) with TMS as an internal standard. The mass spectrometric (HRESIMS) data were acquired using an API QSTAR Pulsar mass spectrometer (Bruker, Bremen, Germany) and an AB SCIEX Trip TOF 5600+ mass spectrometer (SCIEX, Framingham, MA, USA). Optical rotations were measured with a JASCO P-1020 digital polarimeter (Jasco, Tokyo, Japan). IR spectra were recorded on a Shimadzu UV2550 spectrophotometer (Shimadzu, Kyoto, Japan). UV spectra and ECD data were collected using a JASCO J-715 spectropolarimeter (Jasco, Tokyo, Japan). Semipreparative HPLC was carried out using an ODS column (YMC-pack ODS-A, 10 × 250 mm, 5 μm, 4 mL/min, YMC, Kyoto, Japan).

### 3.2. Fungus Material

The fungus *Penicillium* sp. KFD28 (GenBank accession No. MK934323) was isolated from a bivalve mollusk, *Meretrix lusoria*, collected from Haikou Bay, Hainan province, in China. A reference culture of *Penicillium* sp. KFD28 is deposited in our laboratory and maintained at −80 °C.

### 3.3. Culture Conditions

The fungus *Penicillium* sp. KFD28 was cultured in 200 × 1000 mL Erlenmeyer flasks containing 100 g rice and 100 mL of water (33 g sea salt, 5 g L-tryptophan per liter pure water). The fungus was cultured in the medium and incubated at room temperature for thirty days.

### 3.4. Extraction and Isolation

The fermented material was extracted three times with EtOAc to obtain 300 g crude extract. The extract was extracted between petroleum ether and 90% methanol (1:1) to remove the oil. The secondary metabolites extract (46 g) was subjected to a silica gel VLC column, eluting with a stepwise gradient of petroleum ether-EtOAc (10:1, 8:1, 6:1, 4:1, 2:1, 1:1, 1:2, *v/v*) to yield ten subfractions (Fr. 1–Fr. 10).

Fr. 3 (4.2 g) was applied to ODS silica gel with gradient elution of MeOH-H_2_O (1:4, 3:7, 2:3, 1:1, 3:2, 7:3, 4:1, 9:1, 0:1, *v/v*) and afforded nine subfractions (Fr. 3-1–Fr. 3-9). Fr. 3-9 (182 mg) was applied to semipreparative HPLC (YMC-pack ODS-A, 5 μm; 10 × 250 mm; 85% MeOH/H_2_O; 4 mL/min) to obtain compounds **16** (*t*_R_ 20.0 min, 1.0 mg), **11** (*t*_R_ 23.0 min, 9.7 mg), and **8** (*t*_R_ 32.0 min, 20.8 mg). Fr. 5 (3.8 g) was applied to ODS silica gel with gradient elution of MeOH-H_2_O (1:9, 1:4, 3:7, 2:3, 1:1, 3:2, 7:3, 4:1, 9:1, 0:1, *v/v*) to get ten subfractions. Fr. 5-10 (323 mg) was purified by semipreparative HPLC (YMC-pack ODS-A, 5 μm; 10 × 250 mm; 80% MeOH/H_2_O; 4 mL/min) to give compound **15** (*t*_R_ 28.5 min, 53.4 mg). Fr. 6 (5.1 g) was separated by ODS silica gel with gradient elution of MeOH-H_2_O (1:9, 1:4, 3:7, 2:3, 1:1, 3:2, 7:3, 4:1, 9:1, 0:1, *v/v*) to yield ten subfractions (Fr. 6-1–Fr. 6-10). Fr. 6-10 (1.1 g) was subjected to semipreparative HPLC (YMC-pack ODS-A, 5 μm; 10 × 250 mm; 65% MeCN/H_2_O; 4 mL/min) to give compounds **5** (*t*_R_ 11.5 min, 182.6 mg) and **6** (*t*_R_ 18.0 min, 25.8 mg). Fr. 7 (4.7 g) was applied to ODS silica gel with gradient elution of MeOH-H_2_O (1:9, 1:4, 3:7, 2:3, 1:1, 3:2, 7:3, 4:1, 9:1, 0:1, *v/v*), ten subfractions (Fr. 7-1–7-10) were obtained. Fr. 7-7 (587 mg) was eluted with MeCN-H_2_O through ODS silica gel (3:7, 2:3, 1:1, 3:2, 7:3, *v/v*) to afford five subfractions (Fr. 7-7-1–7-7-5). Fr. 7-7-4 (117 mg) was separated by semipreparative HPLC (YMC-pack ODS-A, 5 μm; 10 × 250 mm; 70% MeCN/H_2_O; 4 mL/min) to give compounds **2** (*t*_R_ 8.6 min, 0.6 mg), **3** (*t*_R_ 9.4 min, 1.8 mg), **1** (*t*_R_ 10.4 min, 2.0 mg), **13** (*t*_R_ 11.6 min, 0.6 mg), **14** (*t*_R_ 13.5 min, 9.9 mg), and **7** (*t*_R_ 22.0 min, 8.1 mg). Fr. 7-7-5 (137 mg) was applied to semipreparative HPLC (YMC-pack ODS-A, 5 μm; 10 × 250 mm; 70% MeOH/H_2_O; 4 mL/min) to get compounds **9** (*t*_R_ 14.0 min, 2.0 mg) and **10** (*t*_R_ 18.4min, 3.4 mg). Fr. 8 (5.8 g) was subjected to ODS silica gel with gradient elution of MeOH-H_2_O (1:4, 3:7, 2:3, 1:1, 3:2, 7:3, 4:1, 9:1, 0:1, *v/v*) to get nine subfractions. Then, Fr. 8-7 (925 mg) was eluted through ODS silica gel column of MeCN-H_2_O (1:4, 3:7, 2:3, 1:1, 3:2, *v/v*) to get five subfractions (Fr. 8-7-1–8-7-5). Fr. 8-7-5 (29 mg) was separated by a semipreparative HPLC (YMC-pack ODS-A, 5 μm; 10 × 250 mm; 60% MeCN/H_2_O; 4 mL/min) to yield compound **4** (*t*_R_ 13.0 min, 2.2 mg). Fr. 8-9 (812 mg) was eluted by a semipreparative HPLC (YMC-pack ODS-A, 5 μm; 10 × 250 mm; 80% MeOH/H_2_O; 4 mL/min) to afford compound **12** (*t*_R_ 30.0 min, 157.9 mg).

Penerpene K (**1**): Yellow oil; ${\left[\alpha \right]}_{D}^{25}$−23.0 (*c* 0.01, MeOH); UV (MeOH) *λ*_max_ (log*ε*): 231 (3.41), 280 (2.77) nm; ECD (0.38 mM, MeOH) *λ*_max_ (∆*ε*): 204 (2.46), 223 (−6.08), 255 (2.89); 294 (−0.62) nm; IR (KBr) *ν*_max_: 3412, 2933, 1640, 1606, 1458, 1382, 1307, 1257, 1212, 1160, and 1088 cm^−1^; ^1^H and ^13^C NMR spectral data, Table 1 and Table 2; HRESIMS *m/z* 476.2565 ([M+K]^+^ calcd 476.2562).

Penerpene L (**2**): Yellow oil; ${\left[\alpha \right]}_{D}^{25}$−22.0 (*c* 0.01, MeOH); UV (MeOH) *λ*_max_ (log*ε*): 231 (3.15), 282 (2.41) nm; ECD (1.10 mM, MeOH) *λ*_max_ (∆*ε*): 203 (0.14), 224 (−1.63), 260 (0.58), 292 (−0.08), 330 (0.24) nm; IR (KBr) *ν*_max_: 3411, 2935, 1645, 1604, 1452, 1381, 1307, 1256, 1158, 1074, and 1018 cm^−1^; ^1^H and ^13^C NMR spectral data, Table 1 and Table 2; HRESIMS *m/z* 476.2210 ([M+K]^+^ calcd 476.2198).

Penerpene M (**3**): White powder; ${\left[\alpha \right]}_{D}^{25}$+15.0 (*c* 0.01, MeOH); UV (MeOH) λ_max_ (log*ε*): 226 (2.84), 261 (2.32) nm; ECD (1.15 mM, MeOH) λ_max_ (∆*ε*): 198 (1.32), 202 (−1.27), 221 (−0.92), 263 (0.94) nm; IR (KBr) *ν*_max_: 3383, 2931, 1648, 1609, 1575, 1357, 1316, 1257, 1208, 1160, 1118, and 1044 cm^−1^; ^1^H and ^13^C NMR spectral data, Table 1 and Table 2; HRESIMS *m/z* 446.3020 ([M+Na]^+^ calcd 446.3030).

Penerpene N (**4**): Yellow oil; ${\left[\alpha \right]}_{D}^{25}$+21.0 (*c* 0.01, MeOH); UV (MeOH) λ_max_ (log*ε*): 231 (3.05), 277 (2.44) nm; ECD (0.77mM, MeOH) λ_max_ (∆*ε*): 197 (−3.23), 219 (4.27), 238 (−6.39), 257 (3.21) nm; IR (KBr) *ν*_max_: 3415, 2937, 1641, 1408, 1110, 1408, 1110, 1042, 922, 852, 688, and 565 cm^−1^; ^1^H and ^13^C NMR spectral data, Table 1 and Table 2; HRESIMS *m/z* 434.2338 ([M-H]^‒^ calcd 434.2331).

## 4. Conclusions

To summarize, based on the OSMAC culture strategy, four new indole-diterpenoids were isolated from the marine-derived fungus *Penicillium* sp. KFD28 secondary metabolites. The absolute configurations of new compounds **1**–**3** were determined by spectroscopic methods coupled with experimental and calculated ECD. New compound **4** showed mild cytotoxicity against the human cervical cancer cell line HeLa. Notably, compound **9** exhibited strong cytotoxic activity against the human liver cancer cell line BeL-7402 with IC_50_ values of 5.3 μM, indicating that compound **9** deserves further study for its therapeutic potential to develop new anti-hepatoma drugs. Compound **7** showed pronounced antibacterial activity against *Bacillus subtilis* with MIC values of 16 μg/mL, which had the potential to become an antibiotic. However, the mechanisms causing cytotoxicity and bacteria restraint were unknown and require further study. In general, this study expanded the application of the OSMAC method to increase the chemical and biological diversity of natural products isolated from the *Penicillium* sp. KFD28.

## Figures and Tables

**Table 1:** ^1^H NMR (500 and 600 MHz) data of **1**–**4** in DMSO-*d*_6_.

Position	1	2	3	4
	*δ*_H_ (*J* in Hz)	*δ*_H_ (*J* in Hz)	*δ*_H_ (*J* in Hz)	*δ*_H_ (*J* in Hz)
2			7.04 (1H, s)	
4			7.55 (1H, d, 8.0)	
5	1.92 (1H, m)	1.88 (1H, overlap)	6.93 (1H, t, 8.0)	2.01 (1H, m)
	1.80 (1H, m)	1.60 (1H, overlap)		1.94 (1H, m)
6	1.51 (1H, m)	1.90 (1H, m)	7.02 (1H, t, 8.0)	1.72 (1H, overlap)
	1.62 (1H, overlap)	1.48 (1H, m)		2.20 (1H, m)
7	3.03 (1H, dd, 12.1, 3.8)	3.49 (1H, dd, 9.7, 7.0)	7.30 (1H, d, 8.0)	4.46 (1H, m)
8			2.07 (1H, m)	
			3.15 (1H, d, 13.3)	
9	3.10 (1H, dd, 12.0, 2.7)		1.81 (1H, m)	5.05 (1H, s)
10	1.41 (1H, m)	4.04 (1H, dd, 2.1, 6.7)	1.10 (1H, m)	
	1.62 (1H, overlap)		1.65 (1H, m)	
11	1.74 (1H, m)	1.88 (1H, overlap)	1.26 (2H, m)	
	1.69 (1H, s)	1.70 (1H, m)		
12			1.81 (1H, m)	5.54 (1H, s)
13	1.39 (1H, m)	2.06 (1H, dd, 12.7, 3.1)		
14	0.83 (1H, m)	1.74 (1H, m)		2.55 (1H, m)
	2.35 (1H, m)	1.64 (1H, m)		
15	1.44 (1H, m)	1.78 (1H, m)	1.42 (2H, overlap)	1.53 (1H, m)
	1.62 (1H, m)	1.60 (1H, overlap)		1.62 (1H, overlap)
16	2.61 (1H, m)	2.86 (1H, m)	1.24 (1H, overlap)	1.72 (1H, overlap)
			1.54 (1H, m)	1.62 (1H, overlap)
17	2.54 (1H, m)	2.61 (1H, m)	3.27 (1H, m)	2.68 (1H, m)
	2.21 (1H, dd, 12.3, 10.9)	2.29 (1H, dd, 11.2, 12.6)		
18				2.33 (1H, m)
				2.61 (1H, m)
19			1.02 (1H, m)	
			1.47 (1H, m)	
20	7.27 (1H, d, 7.5)	7.27 (1H, d, 7.5)	1.84 (2H, m)	
21	6.91 (1H, t, 7.5)	6.90 (1H, m)	5.05 (1H, t, 7.6)	7.27 (1H, m)
22	6.91 (1H, t, 7.5)	6.94 (1H, m)		6.92 (1H, t, 7.9)
23	7.24 (1H, d, 7.5)	7.26 (1H, d, 7.5)	1.55 (3H, s)	6.96 (1H, t, 7.9)
24			1.62 (3H, s)	7.27 (1H, m)
25	0.94 (3H, s)	0.94 (3H, s)	0.94 (3H, s)	
26	1.13 (3H, s)	1.17 (3H, s)	1.11 (3H, s)	1.01 (3H, s)
27			0.62 (3H, s)	0.82 (3H, s)
28	1.08 (3H, s)	1.26 (3H, s)		
29	1.03 (3H, s)	1.24 (3H, s)		1.14 (3H, s)
30	3.69 (1H, m)			1.13 (3H, s)
	3.86 (1H, m)			
1-NH	10.55 (1H, s)	10.68 (1H, s)	10.67 (1H, s)	10.72(1H, s)
14-OH			4.04 (1H, s)	
17-OH			4.22 (1H, d, 4.8)	
10-OH		4.88 (1H, s)		
27-OH	4.16 (1H, s)			
28-OH				4.77 (1H, s)
30-OH	4.07 (1H, s)			

Compound **1** was measured with 500 MHz ^1^H NMR. Compounds **2**, **3**, and **4** were measured with 600 MHz ^1^H NMR.

**Table 2:** ^13^C NMR (125 and 150 MHz) data of **1**–**4** in DMSO-*d*_6_.

Position	1	2	3	4
	*δ_C_*	*δ_C_*	*δ_C_*	*δ_C_*
2	151.5, C	150.9, C	122.6, CH	150.2, C
3	52.9, C	50.8, C	114.4, C	42.5, C
3a			127.6, C	
4	39.9, C	38.5, C	118.7, CH	50.0, C
5	32.7, CH_2_	30.6, CH_2_	117.9, CH	30.8, CH_2_
6	24.5, CH_2_	23.2, CH_2_	120.6, CH	30.0, CH_2_
7	85.2, CH	78.2, CH	111.2, CH	83.0, CH
7a			136.3, C	
8			25.5, CH_2_	
9	84.9, CH	109.8, C	42.9, CH	102.3, CH
10	25.7, CH_2_	73.1, CH	27.2, CH_2_	
11	23.8, CH_2_	43.7, CH_2_	20.7, CH_2_	166.8, C
12	48.8, C	84.6, C	41.1, CH	113.1, CH
13	46.9, CH	38.8, CH	42.0, C	163.1, C
14	31.8, CH_2_	26.2, CH_2_	76.4, C	42.8, CH
15	21.3, CH_2_	25.2, CH_2_	29.9, CH_2_	25.6, CH_2_
16	49.0, CH	49.0, CH	28.5, CH_2_	23.9, CH_2_
17	27.3, CH_2_	27.1, CH_2_	71.5, CH	48.7, CH
18	116.1, C	115.9, C	40.6, C	26.9, CH_2_
19	124.6, C	124.4, C	37.0, CH_2_	115.8, C
20	117.8 CH	117.7, CH	21.2, CH_2_	124.3, C
21	118.6, CH	118.5, CH	125.3, CH	117.7, CH
22	119.3, CH	119.4, CH	129.8, C	118.5, CH
23	112.0, CH	111.9, CH	17.5, CH_3_	119.5, CH
24	140.4, C	140.1, C	25.6, CH_3_	111.9, CH
25	14.8, CH_3_	14.4, CH_3_	14.6, CH_3_	140.1, C
26	19.5, CH_3_	16.7, CH_3_	16.3, CH_3_	14.7, CH_3_
27	70.7, C	70.9, C	17.2, CH_3_	15.4, CH_3_
28	26.9, CH_3_	26.4, CH_3_		69.9, C
29	24.9, CH_3_	24.7, CH_3_		24.0, CH_3_
30	58.5, CH_2_			23.8, CH_3_

Compound **1** was measured with 125 MHz ^13^C NMR. Compounds **2**, **3***,* and **4** were measured with 150 MHz ^13^C NMR.

**Table 3:** Cytotoxicity of compounds **4**, **9**, and **15**.

Compound	IC_50_ (μM)		
	HeLa	A549	BeL-7402
**4**	36.3	>50	>50
**9**	>50	28.4	5.3
**15**	33.1	24.4	40.6
Cisplatin ^a^	8.6	4.5	4.1

^a^ Positive control.

**Table 4:** Antibacterial activities of compounds **5**, **7**, **10**, **12**, **14**, and **15**.

Compound	MIC(μg/mL)	
	*Staphylococcus aureus* *ATCC 6538*	*Bacillus subtilis* *ATCC 6633*
**5**	128	32
**7**	64	16
**10**	64	64
**12**	64	128
**14**	64	>128
**15**	32	>128
Ampicillin ^a^	<1	<1

^a^ Positive control.

## Supplementary Materials

The following are available online at https://www.mdpi.com/article/10.3390/md19110613/s1, Figures S1–S38: 1D NMR, 2D NMR, IR, and HRESIMS spectra of the new compounds **1**–**4**; Figure S39: the picture of strain *Penicillium* sp. KFD28; Figure S40: Separation flow chart of fungus *Penicillium* sp. KFD28 secondary metabolites extract; Figure S41: HPLC chromatograms of the EtOAc extracts monitored at wavelengths of 230 nm and 274 nm; Figure S42: HPLC chromatograms of the EtOAc extracts using Rice solid medium with L-tryptophan, and compounds **6**, **12**, **14**, **8**, and **11** monitored at wavelengths of 230 nm and 274 nm; Figure S43: ECD curve for compound **4** and computation section of compounds **1**–**3**.

## References

[1] Lindequist U. (2016). Marine-derived pharmaceuticals-challenges and opportunities. Biomol. Ther..

[2] Carroll A.R., Copp B.R., Davis R.A., Keyzers R.A., Prinsep M.R. (2019). Marine natural products. Nat. Prod. Rep..

[3] Hu Y.W., Chen J.H., Hu G.P., Yu J.C., Zhu X., Lin Y.C., Chen S.P., Yuan J. (2015). Statistical research on the bioactivity of new marine natural products discovered during the 28 years from 1985 to 2012. Mar. Drugs.

[4] Li T.T., Wang Y., Li L., Tang M.Y., Meng Q.H., Zhang C., Hua E.B., Pei Y.H., Sun Y. (2021). New cytotoxic cytochalasans from a plant-associated fungus *Chaetomium globosum* kz-19. Mar. Drugs.

[5] Kubota T., Nakamura K., Kurimoto S.I., Sakai K., Fromont J., Gonoi T., Kobayashi J. (2017). Zamamidine D, a manzamine alkaloid from an okinawan *Amphimedon* sp. marine sponge. J. Nat. Prod..

[6] Kong F.D., Zhang S.L., Zhou S.Q., Ma Q.Y., Xie Q.Y., Chen J.P., Li J.H., Zhou L.M., Yuan J.Z., Hu Z. (2019). Quinazoline-containing indole alkaloids from the marine-derived fungus *Aspergillus* sp. HNMF114. J. Nat. Prod..

[7] Guo Y.W., Liu X.J., Yuan J., Li H.J., Mahmud T., Hong M.T., Yu J.C., Lan W.J. (2020). L-tryptophan induces a marine-derived *Fusarium* sp. to produce indole alkaloids with activity against the Zika virus. J. Nat. Prod..

[8] Kong F.D., Fan P., Zhou L.M., Ma Q.Y., Xie Q.Y., Zheng H.Z., Zhang R.S., Yuan J.Z., Dai H.F., Luo D.Q. (2019). Penerpenes A-D, four indole terpenoids with potent protein tyrosine phosphatase inhibitory activity from the marine-derived fungus *Penicillium* sp. KFD28. Org. Lett..

[9] Zhou L.M., Kong F.D., Fan P., Ma Q.Y., Xie Q.Y., Li J.H., Zheng H.Z., Zheng Z.H., Yuan J.Z., Dai H.F. (2019). Indole-diterpenoids with protein tyrosine phosphatase inhibitory activities from the marine-derived fungus *Penicillium* sp. KFD28. J. Nat. Prod..

[10] Bode H.B., Bethe B., Höfs R., Zeeck A. (2002). Big effects from small changes: Possible ways to explore nature’s chemical diversity. ChemBioChem.

[11] Xu W., Gavia D.J., Tang Y. (2014). Biosynthesis of fungal indole alkaloids. Nat. Prod. Rep..

[12] Huang L.H., Xu M.Y., Li H.J., Li J.Q., Chen Y.X., Ma W.Z., Li Y.P., Xu J., Yang D.P., Lan W.J. (2017). Amino acid-directed strategy for inducing the marine-derived fungus *Scedosporium apiospermum* F41–1 to maximize alkaloid diversity. Org. Lett..

[13] Liu S.S., Yang L., Kong F.D., Zhao J.H., Yao L., Yuchi Z.G., Ma Q.Y., Xie Q.Y., Zhou L.M., Guo M.F. (2021). Three new quinazoline-containing indole alkaloids from the marine-derived fungus *Aspergillus* sp. HNMF114. Front. Microbiol..

[14] Hosoe T., Nozawa K., Udagawa S.I., Nakajima S., Kawai K.I. (2008). Structures of new indoloditerpenes, possible biosynthetic precursors of the tremorgenic mycotoxins, penitrems, from *Penicillium crustosum*. Chem. Phar. Bull..

[15] Nozawa K., Horie Y., Udagawa S.I., Kawai K.I., Yamazaki M. (1989). Isolation of a new tremorgenic indologiterpene, 1′-*O*-Acetylpaxilline, from *Emericella striata* and distribution of paxilline in *Emericella* spp.. Chem. Phar. Bull..

[16] Belofsky G.N., Gloer J.B., Wicklow D.T., Dowd P.F. (1995). Antiinsectan alkaloids: Shearinines A-C and a new paxilline derivative from the Ascostromata of *Eupenicillium Shearii*. Tetrahedron.

[17] Fan Y.Q., Wang Y., Liu P.P., Fu P., Zhu T.H., Wang W., Zhu W.M. (2013). Indole-diterpenoids with anti-H1N1 activity from the aciduric fungus *Penicillium camemberti* OUCMDZ-1492. J. Nat. Prod..

[18] Chen M.Y., Xie Q.Y., Kong F.D., Ma Q.Y., Zhou L.M., Yuan J.Z., Dai H.F., Wu Y.G., Zhao Y.X. (2020). Two new indole-diterpenoids from the marine-derived fungus *Penicillium* sp. KFD28. J. Asian Nat. Prod. Res..

[19] Ariantari N.P., Ancheeva E., Wang C.Y., Mándi A., Knedel T.O., Kurtán T., Chaidir C., Müller W.E.G., Kassack M.U., Janiak C. (2019). Indole diterpenoids from an endophytic *Penicillium* sp.. J. Nat. Prod..

[20] Munday-Finch S.C., Wilkins A.L., Miles C.O. (1996). Isolation of paspaline B, an indole-diterpenoid from *Penicillium paxilli*. Phytochemistry.

[21] Liang Z.Y., Shen N.X., Zhou X.J., Zheng Y.Y., Chen M., Wang C.Y. (2020). Bioactive indole diterpenoids and polyketides from the marine-derived fungus *penicillium javanicum*. Chem. Nat. Comp..

[22] Yamaguchi T., Nozawa K., Hosoe T., Nakajima S., Kawai K.I. (1993). Indoloditerpenes related to tremorgenic mycotoxins, penitrems, from *Penicillium crustosum*. Phytochemistry.

[23] Nozawa K., Nakajima S., Kawai K.I., Udagawa S.I. (1988). Isolation and structures of indoloditerpenes, possible biosynthetic intermediates to the tremorgenic mycotoxin, paxilline, from *Emericella striata*. J. Chem. Soc. Perkin Trans..

[24] Kimura Y., Nishibe M., Nakajima H., Hamasaki T., Shigemitsu N., Sugawara F., Stout T.J., Clardy J. (1992). Emeniveol: A new pollen growth inhibitor from the fungus, *Emericella nivea*. Tetrahedr. Lett..

[25] Yang M.H., Wang J.S., Luo J.G., Wang X.B., Kong L.Y. (2011). Chisopanins A-K, 11 new protolimonoids from *chisocheton paniculatus* and their anti-inflammatory activities. Bioorg. Med. Chem..

[26] Luo X.D., Wu S.H., Wu D.G., Ma Y.B., Qi S.H. (2002). Three new apo-tirucallols with six-membered hemiacetal from meliaceae. Tetrahedron.

[27] Chen C., Liang F., Chen B., Sun Z.Y., Xue T.D., Yang R.L., Luo D.Q. (2016). Identification of demethylincisterol A_3_ as a selective inhibitor of protein tyrosine phosphatase Shp2. Eur. J. Phar..

[28] Sallam A.A., Houssen W.E., Gissendanner C.R., Orabi K.Y., Foudah A.I., Sayed K.A.E. (2013). Bioguided discovery and pharmacophore modeling of the mycotoxic indole diterpene alkaloids penitrems as breast cancer proliferation, migration, and invasion inhibitors. Med. Chem. Commun..

[29] Guo J.J., Dai B.L., Chen N.P., Jin L.X., Jiang F.S., Ding Z.S., Qian C.D. (2016). The anti-staphylococcus aureus activity of the phenanthrene fraction from fibrous roots of *Bletilla striata*. BMC Complement. Altern. Med..

