# Supplementary material for: Cytotoxic Indole-Diterpenoids from the Marine-Derived Fungus Penicillium sp. KFD28
Source: Mar Drugs. 2021 Oct 28;19(11):613. doi: 10.3390/md19110613 (PMC8619218; doi:10.3390/md19110613)
Supplement: Supplementary file 1 [file marinedrugs-19-00613-s001.zip › marinedrugs-1419406-supplementary.pdf]

Cytotoxic Indole-Diterpenoids from the Marine-Derived Fungus *Penicillium* sp. KFD28

Lu-Ting Dai [1], Li Yang [2], Fan-Dong Kong [3], Qing-Yun Ma [2], Qing-Yi Xie [2], Hao-Fu Dai [4], Zhi-Fang Yu [1,*], and You-Xing Zhao [2,*]

[1]College of Food Science and Technology, Nanjing Agricultural University, Nanjing 210095, China

[2]Haikou Key Laboratory for Research and Utilization of Tropical Natural Products, Institute of Tropical Bioscience and Biotechnology, CATAS, Haikou 571101, China;

[3]Key Laboratory of Chemistry and Engineering of Forest Products, State Ethnic Affairs Commission, Guangxi Key Laboratory of Chemistry and Engineering of Forest Products, Guangxi Collaborative Innovation Center for Chemistry and Engineering of Forest Products, School of Chemistry and Chemical Engineering, Guangxi University for Nationalities, Nanning 530006, China.

[4]Hainan Institute for Tropical Agricultural Resources, CATAS, Haikou 571101, China.

* Correspondence: yuzhifang@njau.edu.cn (Z.-F.Y.); zhaoyouxing@itbb.org.cn (Y.-X.Z.);
Tel.: +86-139-5169-2350 (Z.-F.Y.); +86-898-6698-9095 (Y.-X.Z.)

List of Supporting Information

NMR, HRESIMS, and IR spectra of compound 1-4 3
Figure S1. The ^{1}H NMR Spectrum of Compound **1** in DMSO 3
Figure S2. The ^{13}C NMR Spectrum of Compound **1** in DMSO 4
Figure S3. The DEPT Spectrum of Compounds **1** in DMSO 5
Figure S4. The HMQC Spectrum of Compound **1** in DMSO 6
Figure S5. The HMBC Spectrum of Compound **1** in DMSO 7
Figure S6. The correlation of H-11 (δ_H 1.69) with C-30 (δ_C 58.5) in HMBC Spectrum of Compound **1** in DMSO 8
Figure S7. The COSY Spectrum of Compound **1** in DMSO 9
Figure S8. The correlation of H$_2$-30 (δ_C 3.86) with 30-OH (δ 4.07) in COSY Spectrum of Compound **1** in DMSO 10
Figure S9. The ROESY Spectrum of Compound **1** in DMSO 11
Figure S10. The HRESIMS Spectroscopic Data of Compound **1** 12
Figure S11. The IR Spectrum of Compound **1** 13
Figure S12. The ^{1}H NMR Spectrum of Compounds **2** in DMSO 14
Figure S13. The ^{13}C NMR Spectrum of Compounds **2** in DMSO 15
Figure S14. The DEPT Spectrum of Compounds **2** in DMSO 16
Figure S15. The HMQC Spectrum of Compounds **2** in DMSO 17
Figure S16. The HMBC Spectrum of Compound **2** in DMSO 18
Figure S17. The COSY Spectrum of Compounds **2** in DMSO 19
Figure S18. The ROESY Spectrum of Compound **2** in DMSO 20
Figure S19. The HRESIMS Spectroscopic Data of Compounds **2** 21
Figure S20. The IR Spectrum of Compounds **2** 22
Figure S21. The ^{1}H NMR Spectrum of Compound **3** in DMSO 23
Figure S22. The ^{13}C NMR Spectrum of Compound **3** in DMSO 24

Figure S23. The DEPT Spectrum of Compound **3** in DMSO 25
Figure S24. The HMQC Spectrum of Compound **3** in DMSO 26
Figure S25. The HMBC Spectrum of Compound **3** in DMSO 27
Figure S26. The COSY Spectrum of Compound **3** in DMSO 28
Figure S27. The ROESY Spectrum of Compound **3** in DMSO 29
Figure S28. The HRESIMS Spectroscopic Data of Compound **3** 30
Figure S29. The IR Spectrum of Compound **3** 31
Figure S30. The ^{1}H NMR Spectrum of Compound **4** in DMSO 32
Figure S31. The ^{13}C NMR Spectrum of Compound **4** in DMSO 33
Figure S32. The DEPT Spectrum of Compound **4** in DMSO 34
Figure S33. The HMQC Spectrum of Compound **4** in DMSO 35
Figure S34. The HMBC Spectrum of Compound **4** in DMSO 36
Figure S35. The COSY Spectrum of Compound **4** in DMSO 37
Figure S36. The ROESY Spectrum of Compound **4** in DMSO 38
Figure S37. The HRESIMS Spectroscopic Data of Compound **4** 39
Figure S38. The IR Spectrum of Compound **4** 40
The strain of *Penicillium* sp. KFD28 41
Figure S39. The picture of strain *Penicillium* sp. KFD28 41
Figure S40. Separation flow chart of *Penicillium* sp. KFD28 methanolic exact 42
Figure S41. HPLC chromatograms of the EtOAc extracts monitored at wavelengths of 230 nm (blue line) and 274 nm (red line). (A) Liquid medium; (B) Solid rice medium with L-tryptophan. 43
Figure S42. HPLC chromatograms of the EtOAc extracts using Solid rice medium with L-tryptophan, and compounds **6**, **12**, **14, 8** and **11** monitored at wavelengths of 230 nm (blue line) and 274 nm (red line)... 44
ECD curve for compound 4 45
Figure S43. ECD curve for compound **4** 45
Computation section 45
Figure S44. Experimental (black bold) spectrum of **1** in methanol and calculated ECD spectra of **1** (red dash).. 46
Figure S45. Optimized geometries of **1** at B3LYP-D3BJ/6-31G(d) level in methanol. 46
Figure S46. Experimental (black bold) spectrum of **2** in methanol and calculated ECD spectra of **2** (red dash).. 47
Figure S47. Optimized geometries of **1** at B3LYP-D3BJ/6-31G(d) level in methanol. 47
Figure S48. Experimental (black bold) spectrum of **3** in methanol and calculated ECD spectra of simplified structure (**3a**) of **3** (red dash)... 47
Figure S49. Optimized geometries of isomers of simplified structure (**3a**) of **3** at B3LYP-D3BJ/6-31G(d) level in methanol.. 48
Table S1. Conformational analysis of the optimized isomers **1** at B3LYP/6-311G(d) level in chloroform. 48

NMR, HRESIMS, and IR spectra of compound 1-4

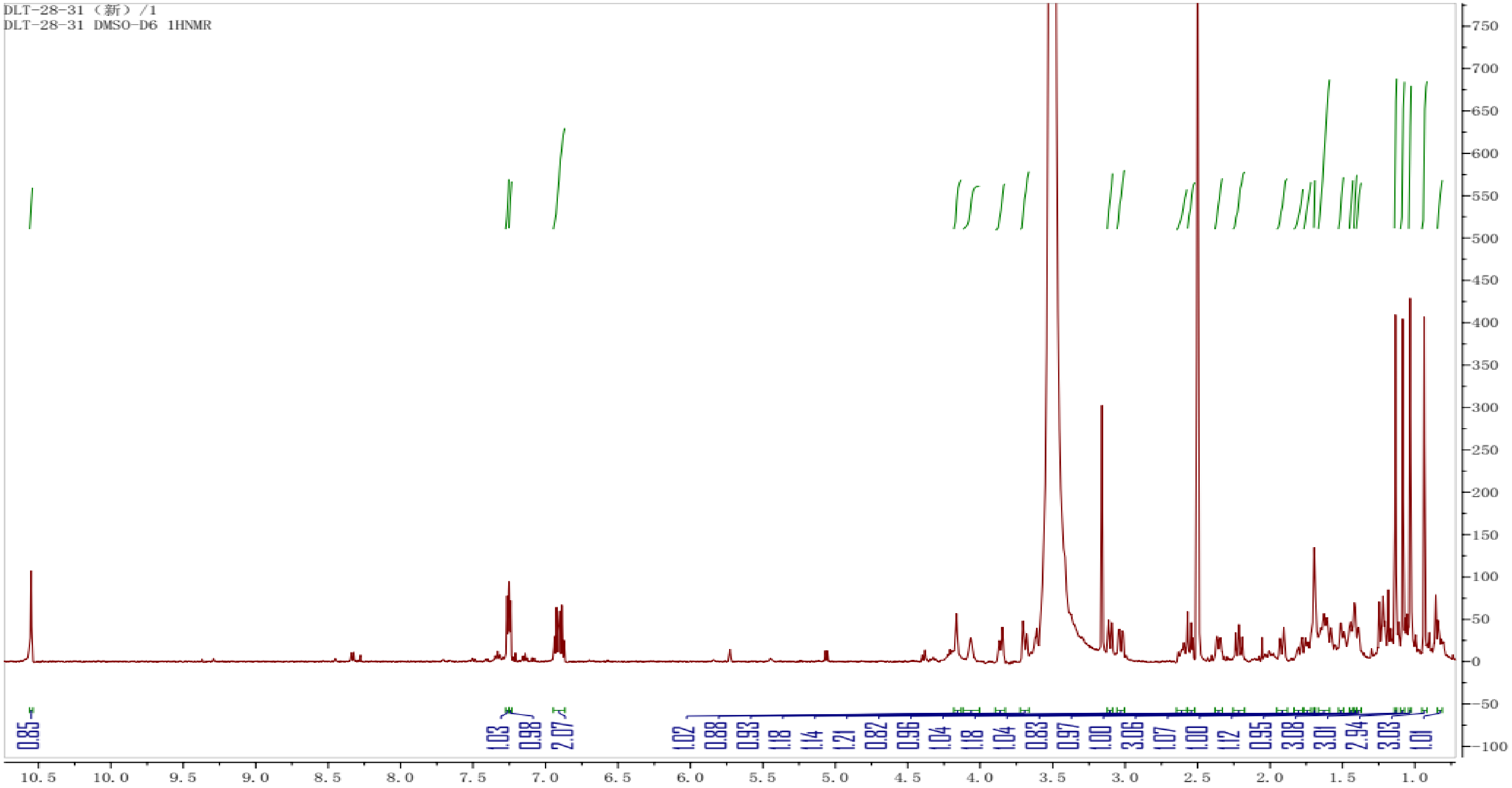

Figure S1. The ^{1}H NMR Spectrum of Compound **1** in DMSO

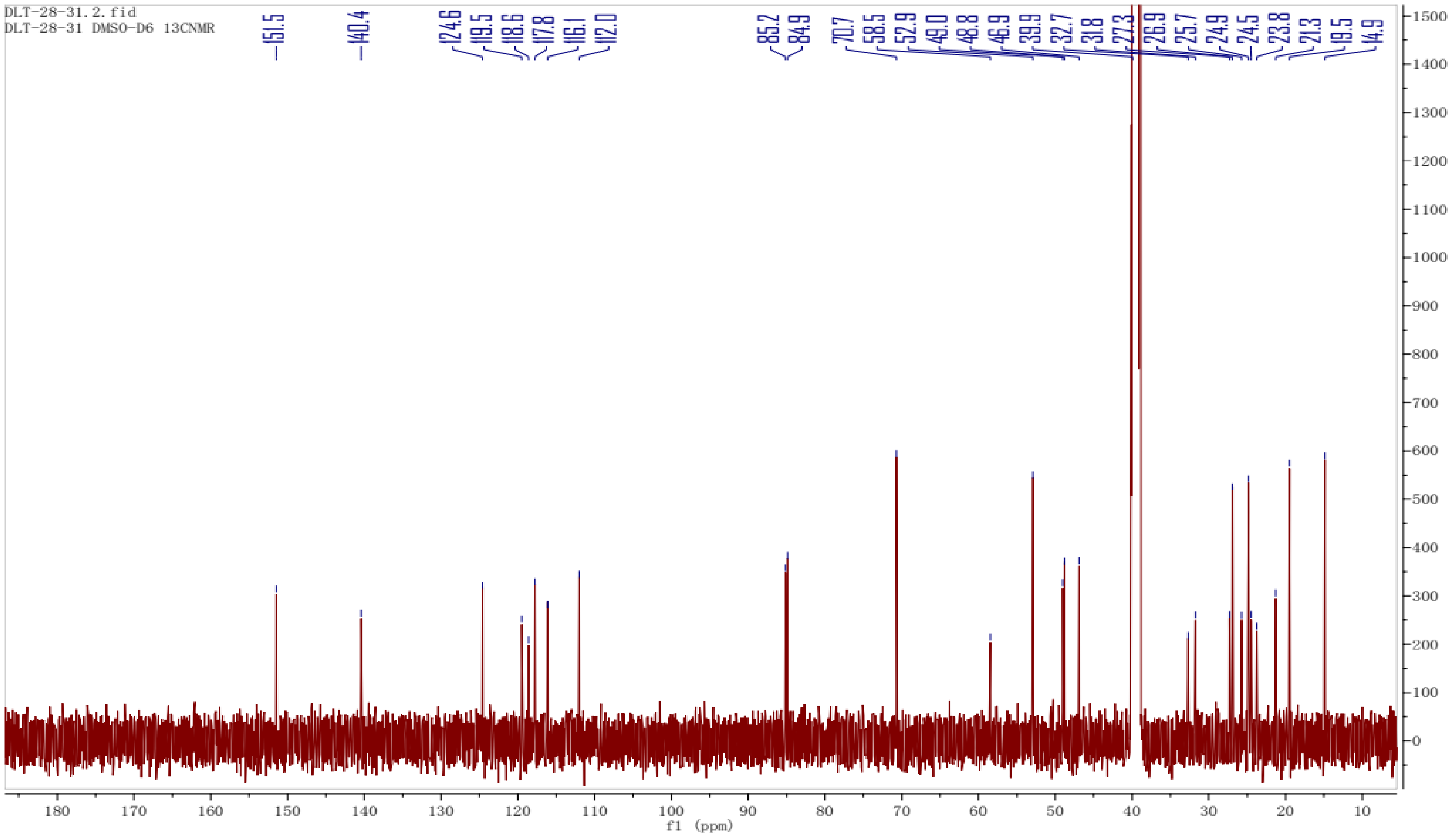

Figure S2. The ^{13}C NMR Spectrum of Compound **1** in DMSO

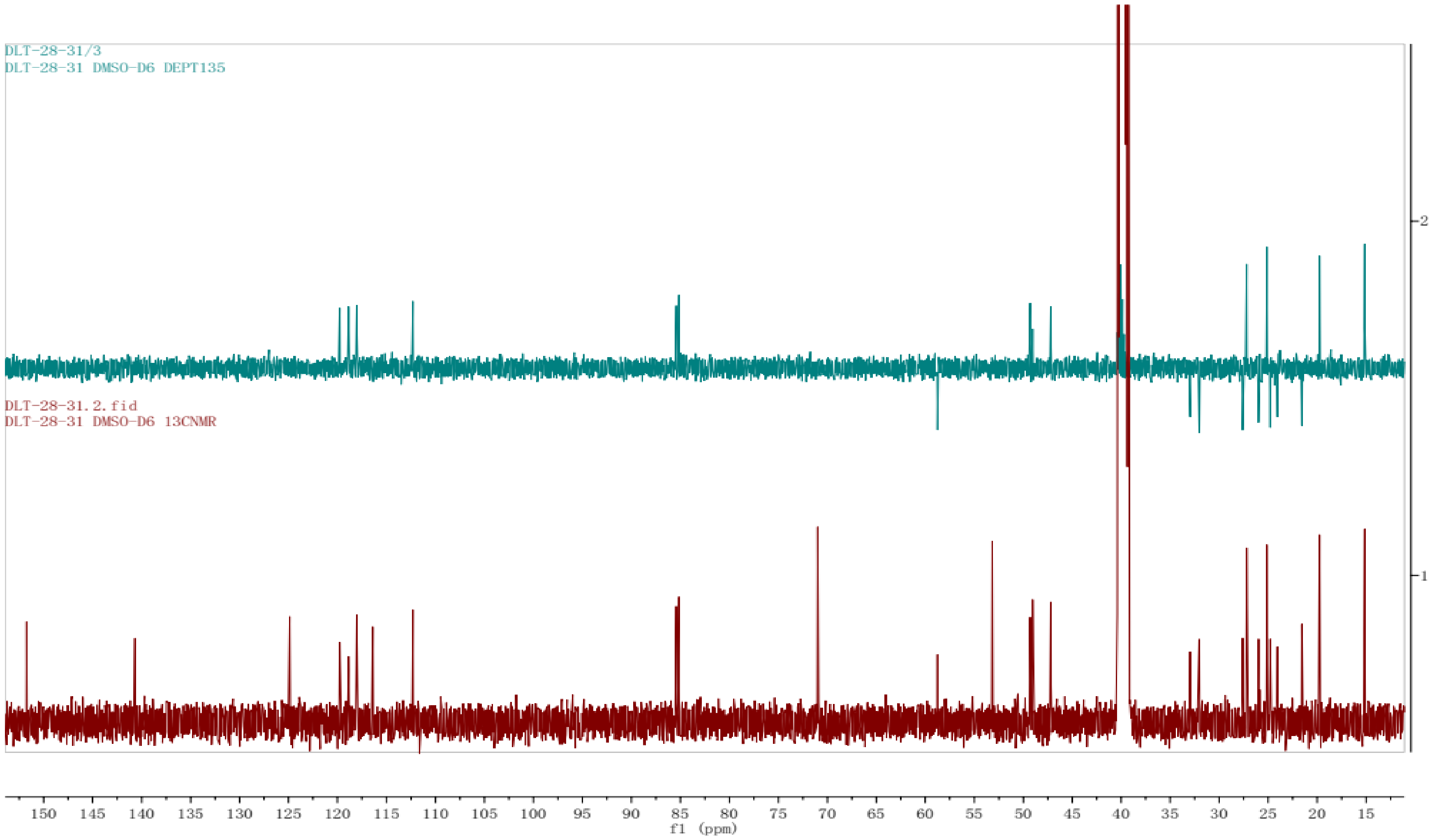

Figure S3. The DEPT Spectrum of Compounds **1** in DMSO

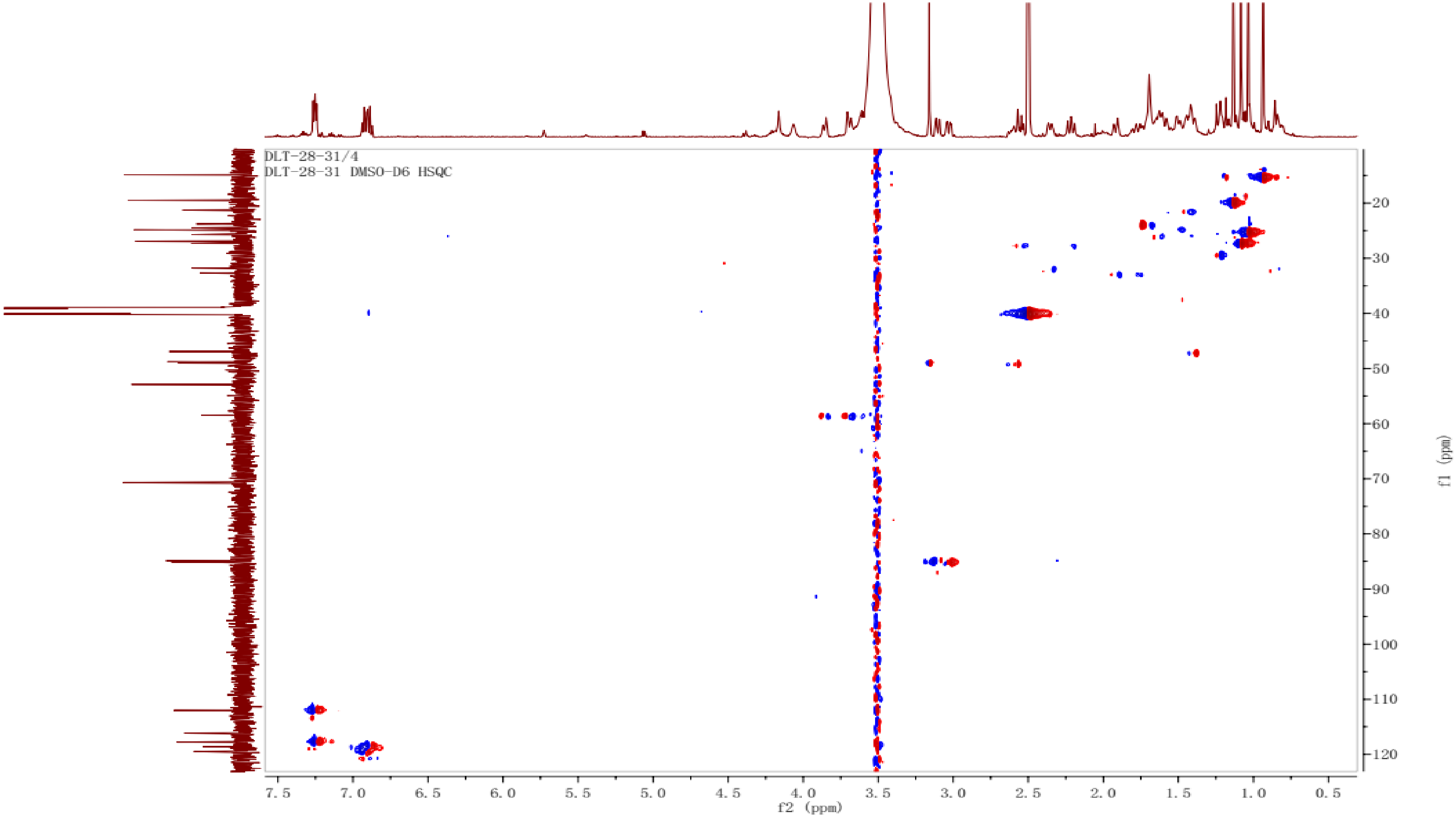

Figure S4. The HMQC Spectrum of Compound **1** in DMSO

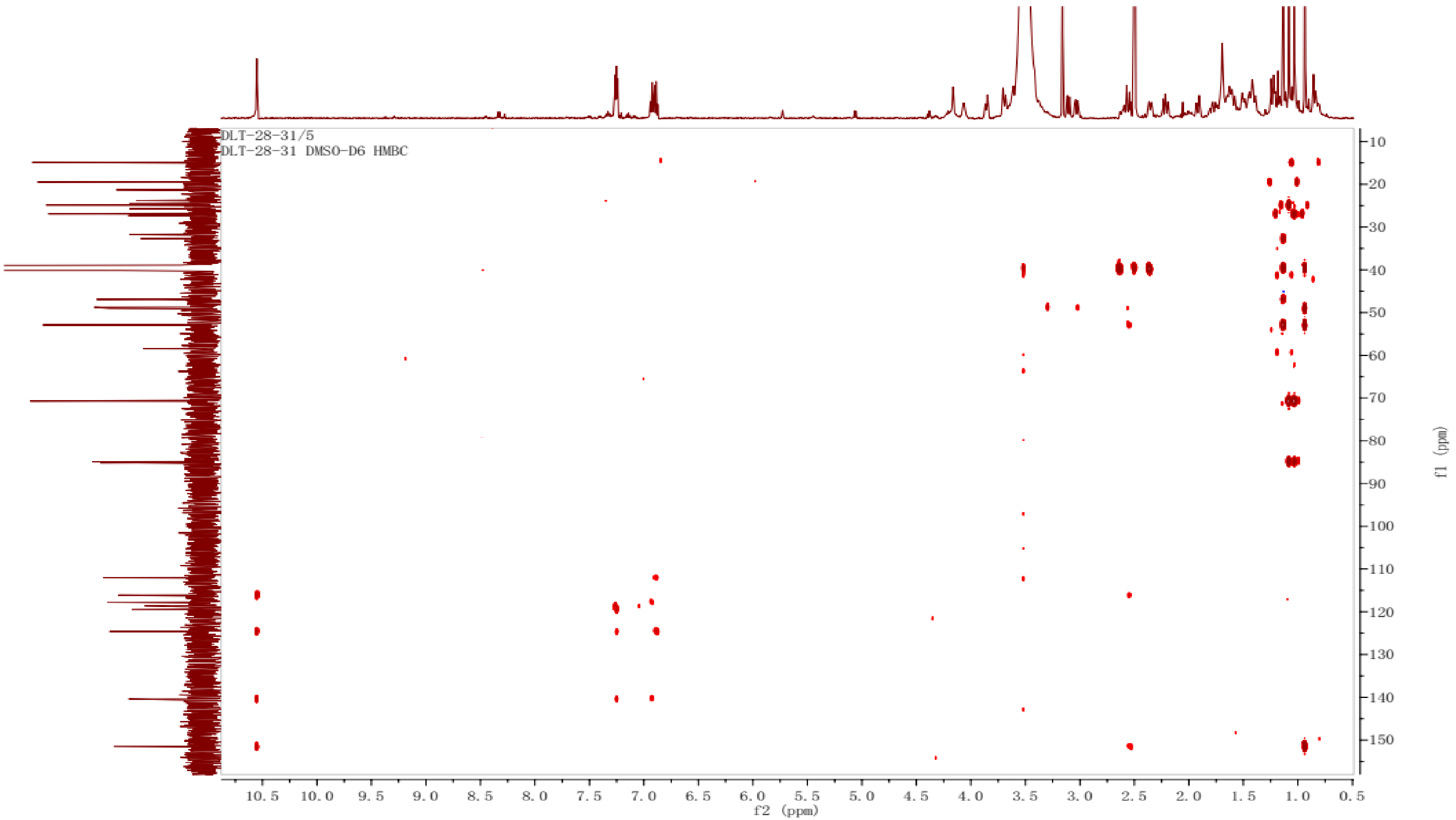

Figure S5. The HMBC Spectrum of Compound **1** in DMSO

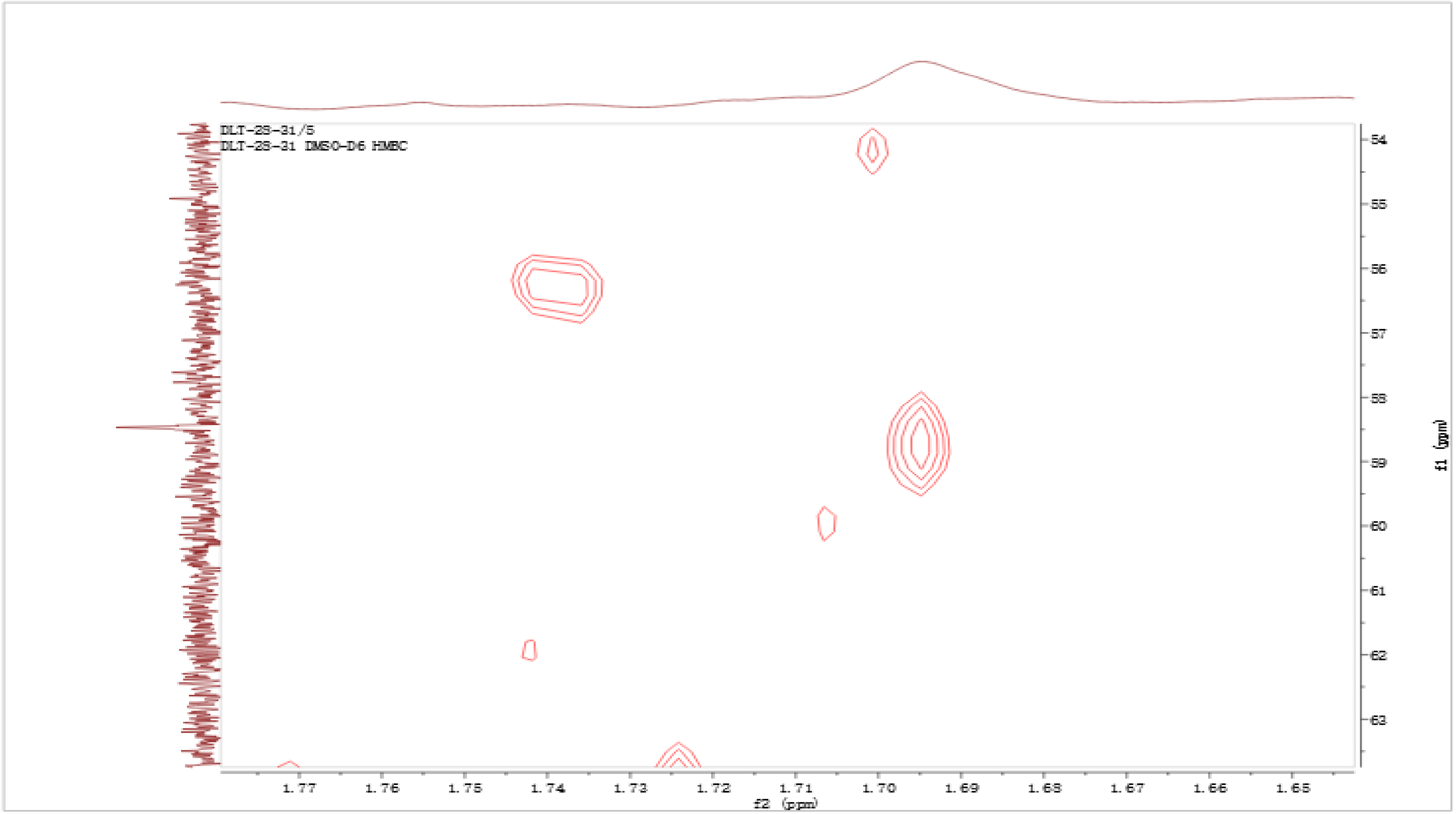

Figure S6. The correlation of H-11 (δ_H 1.69) with C-30 (δ_C 58.5) in HMBC Spectrum of Compound **1** in DMSO

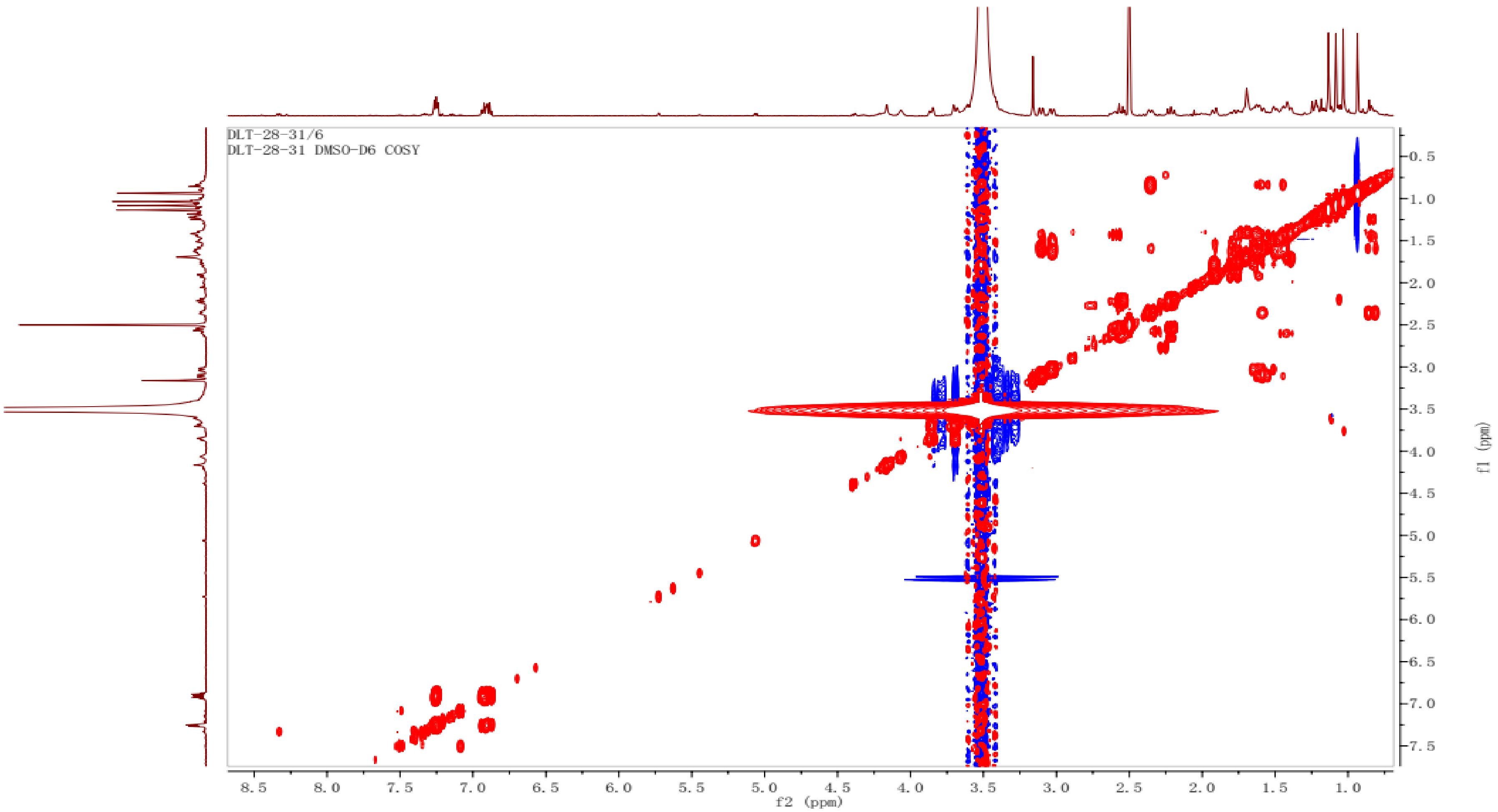

Figure S7. The COSY Spectrum of Compound **1** in DMSO

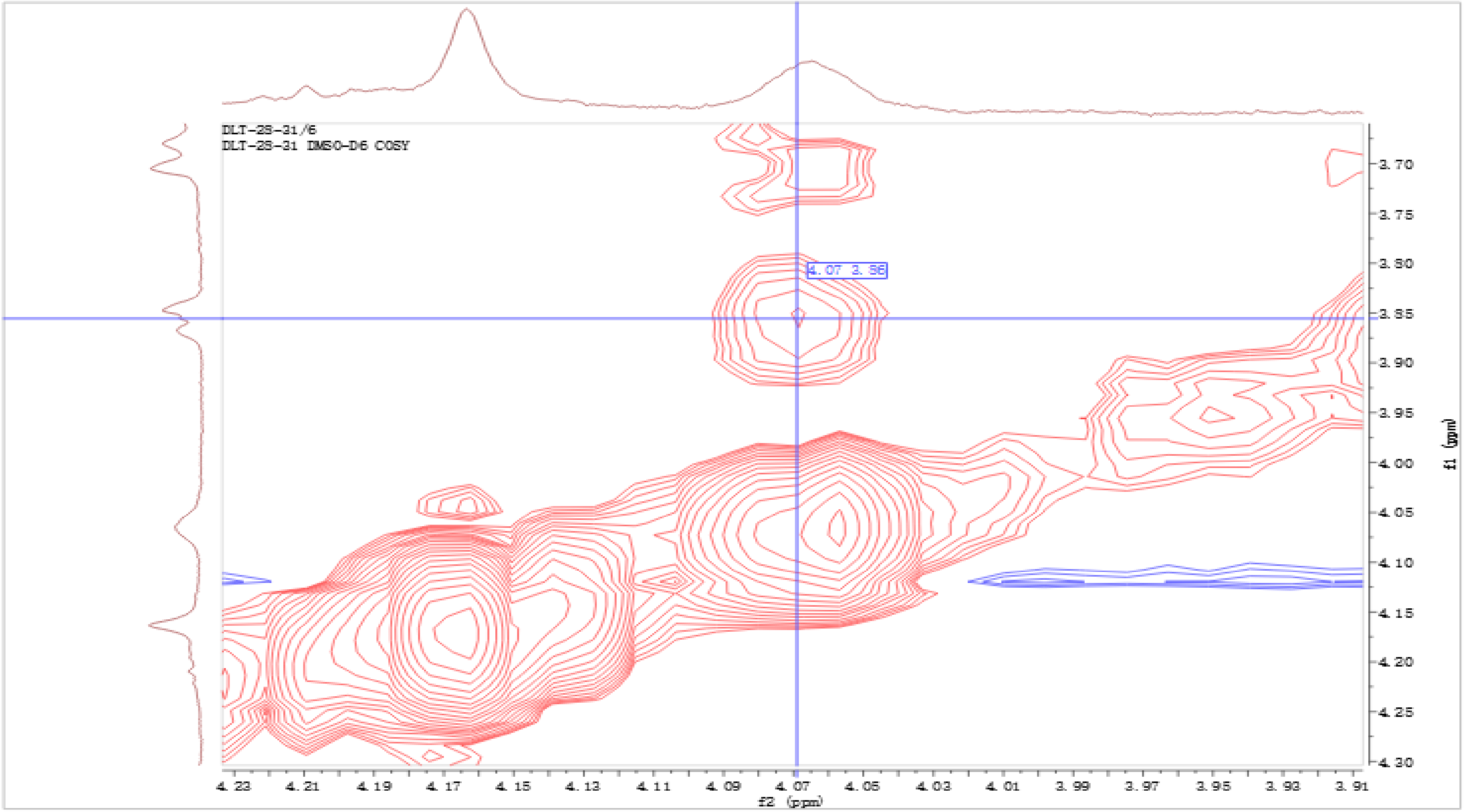

Figure S8. The correlation of H_2-30 (δ_C 3.86) with 30-OH (δ 4.07) in COSY Spectrum of Compound **1** in DMSO

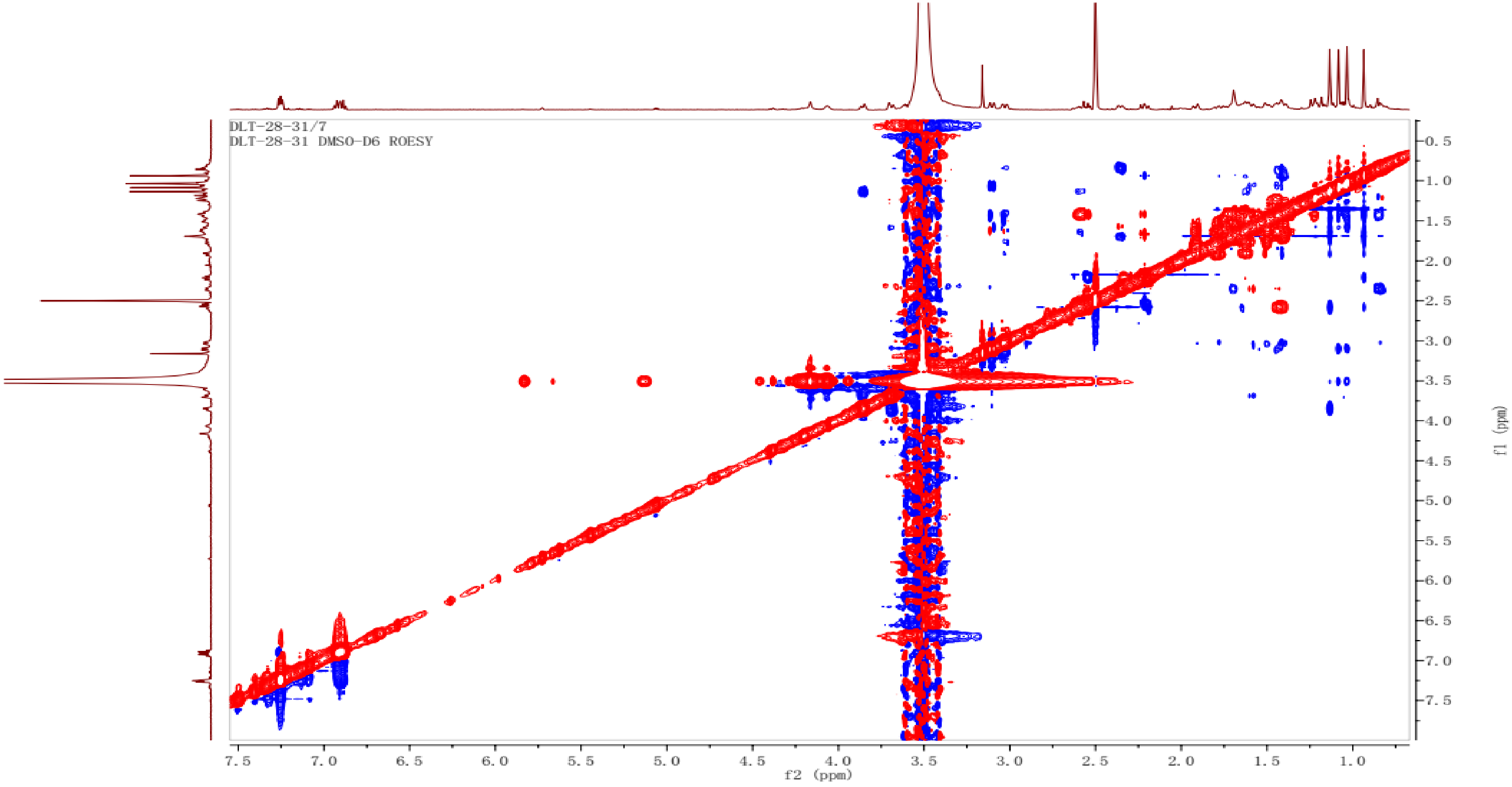

Figure S9. The ROESY Spectrum of Compound **1** in DMSO

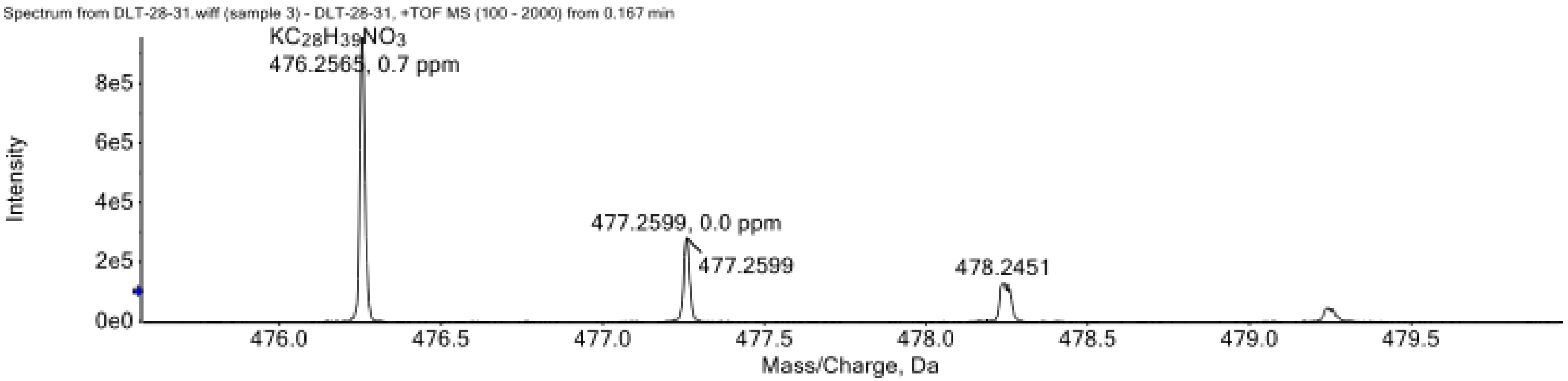

Hit	Formula	m/z	RDB	ppm	MS Rank	MSMS ppm	MSMS Rank	Found
1	C28H39NO3	476.2562	10.0	0.7	1			NA/NA

Figure S10. The HRESIMS Spectroscopic Data of Compound **1**

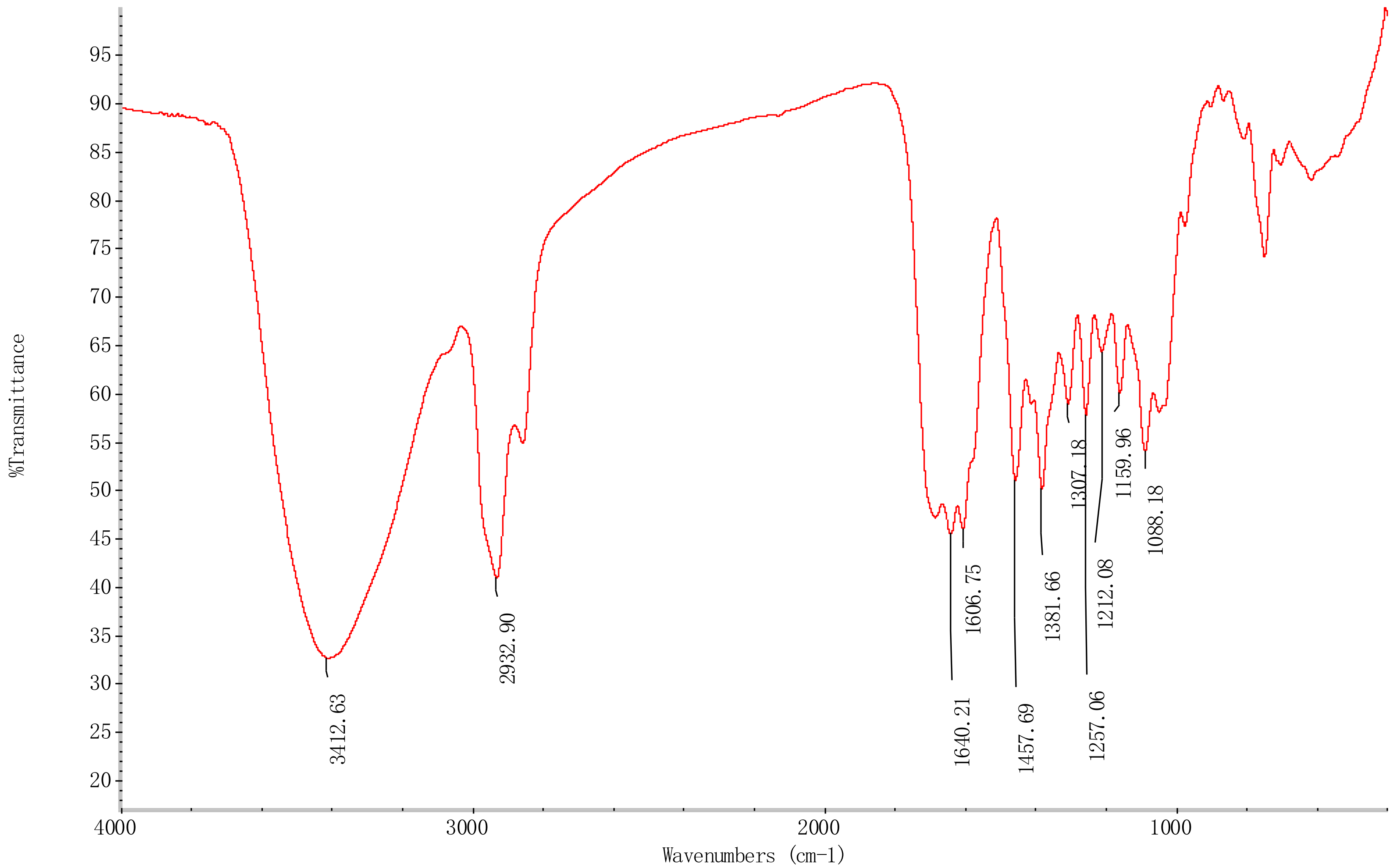

Figure S11. The IR Spectrum of Compound **1**

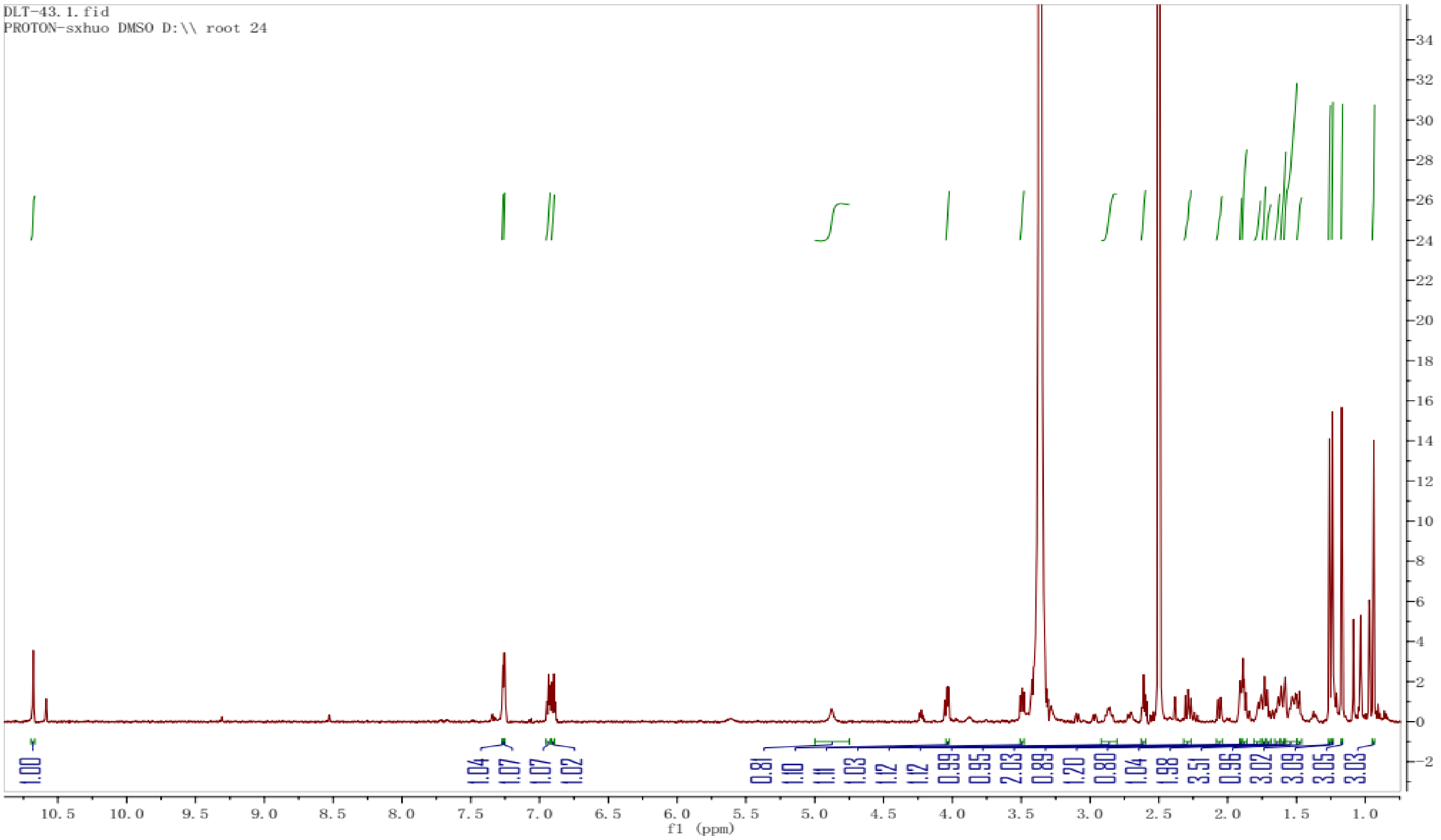

Figure S12. The ^{1}H NMR Spectrum of Compounds **2** in DMSO

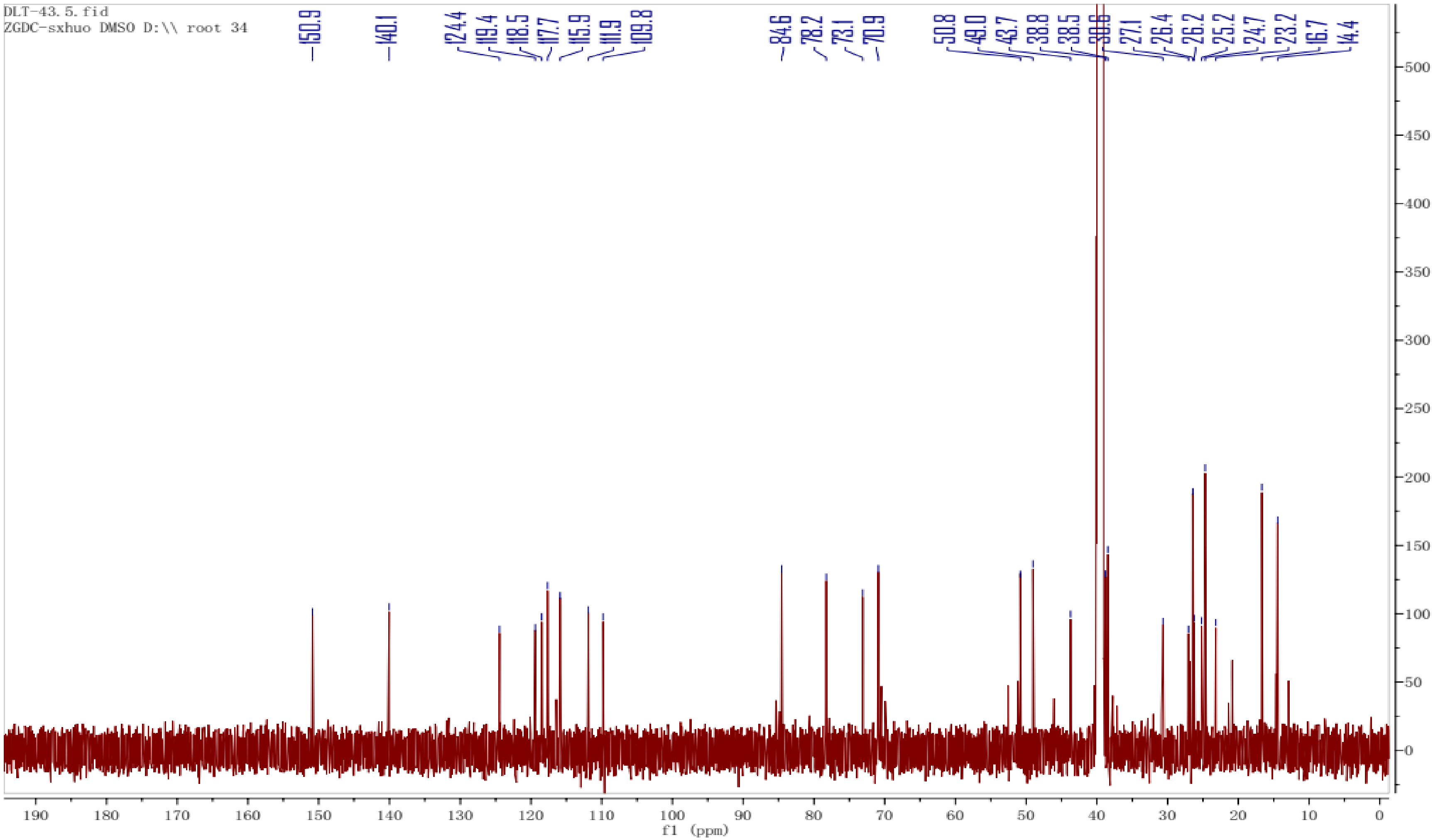

Figure S13. The ^{13}C NMR Spectrum of Compounds **2** in DMSO

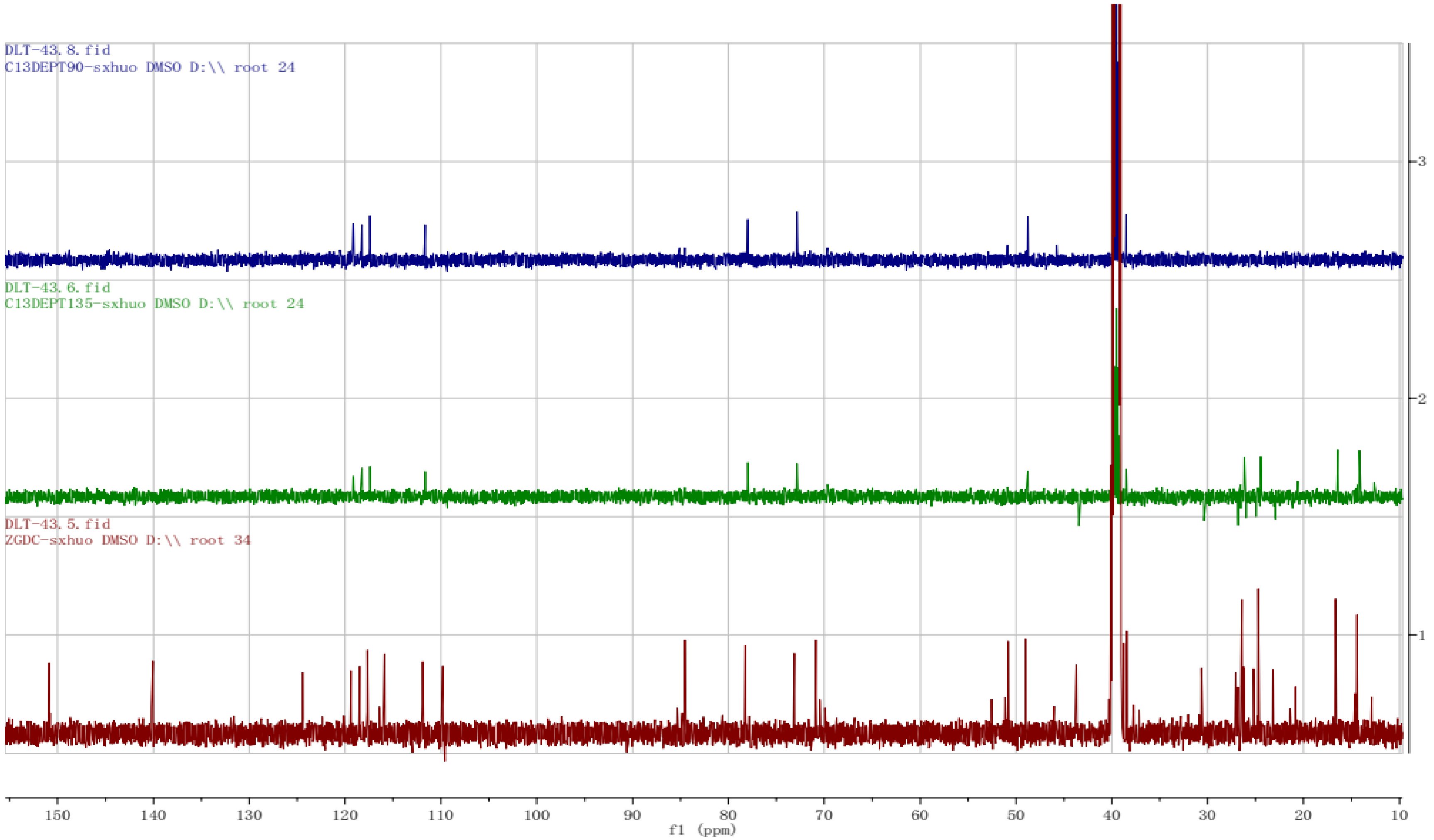

Figure S14. The DEPT Spectrum of Compounds **2** in DMSO

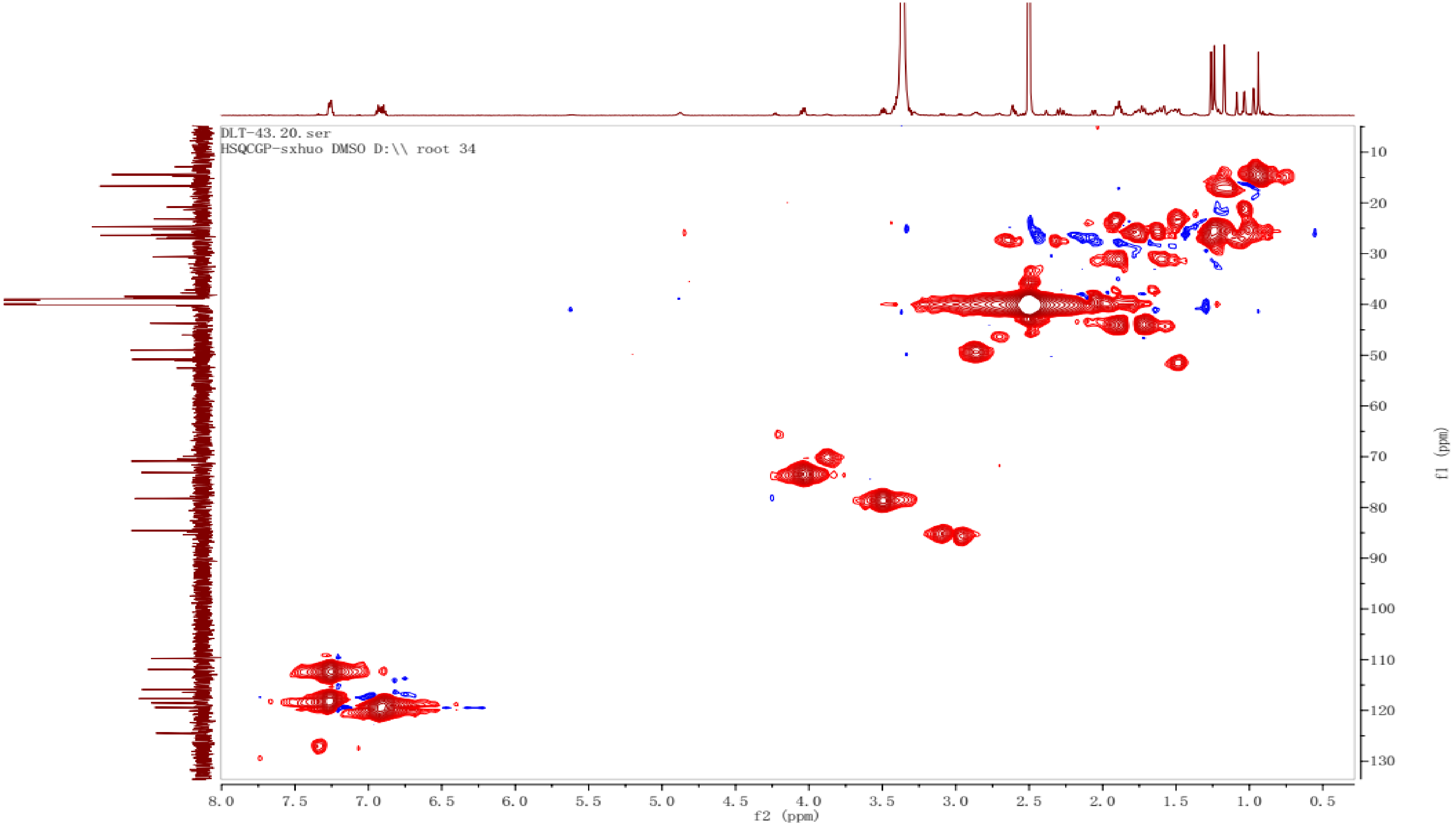

Figure S15. The HMQC Spectrum of Compounds **2** in DMSO

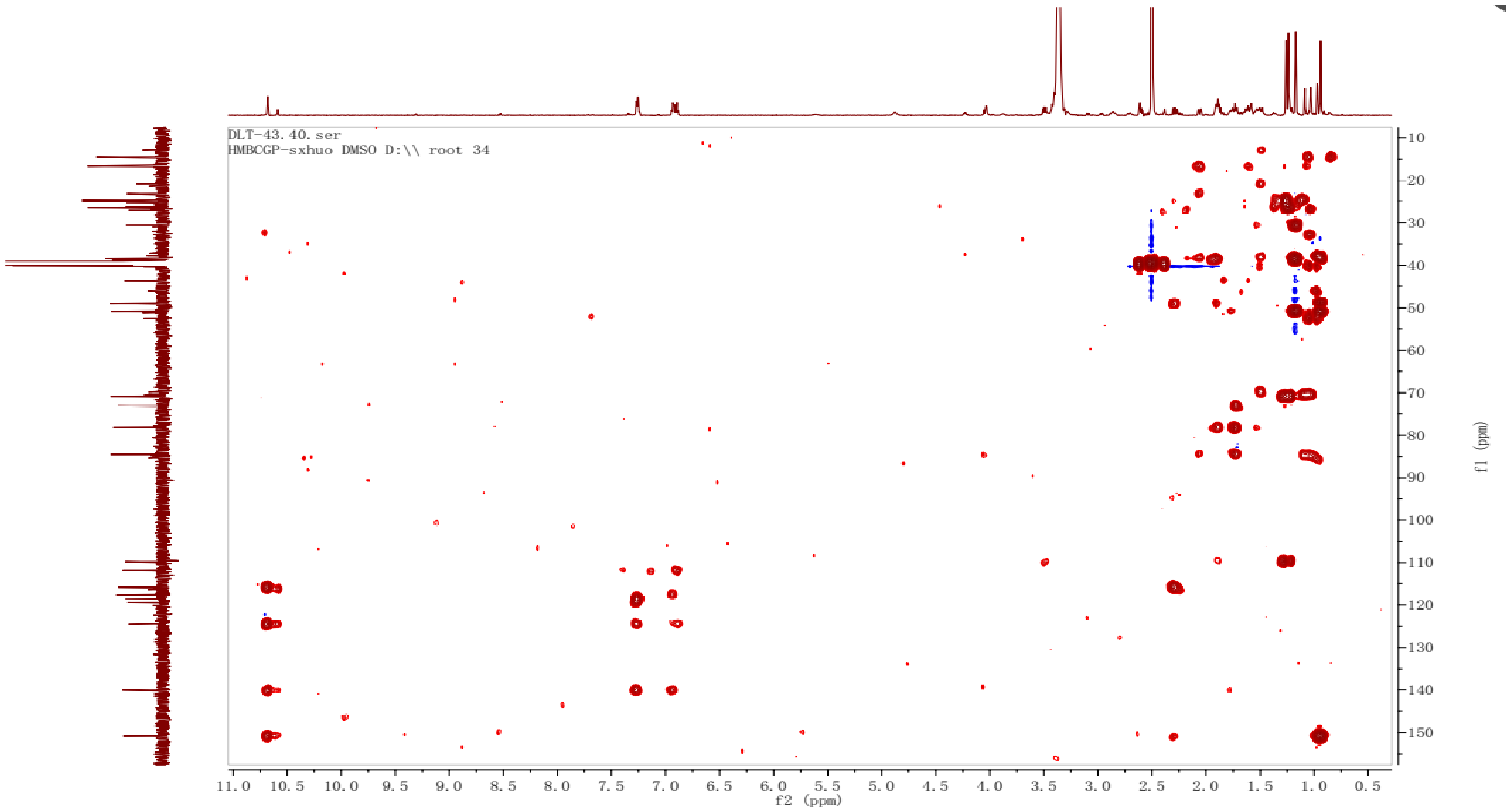

Figure S16. The HMBC Spectrum of Compound **2** in DMSO

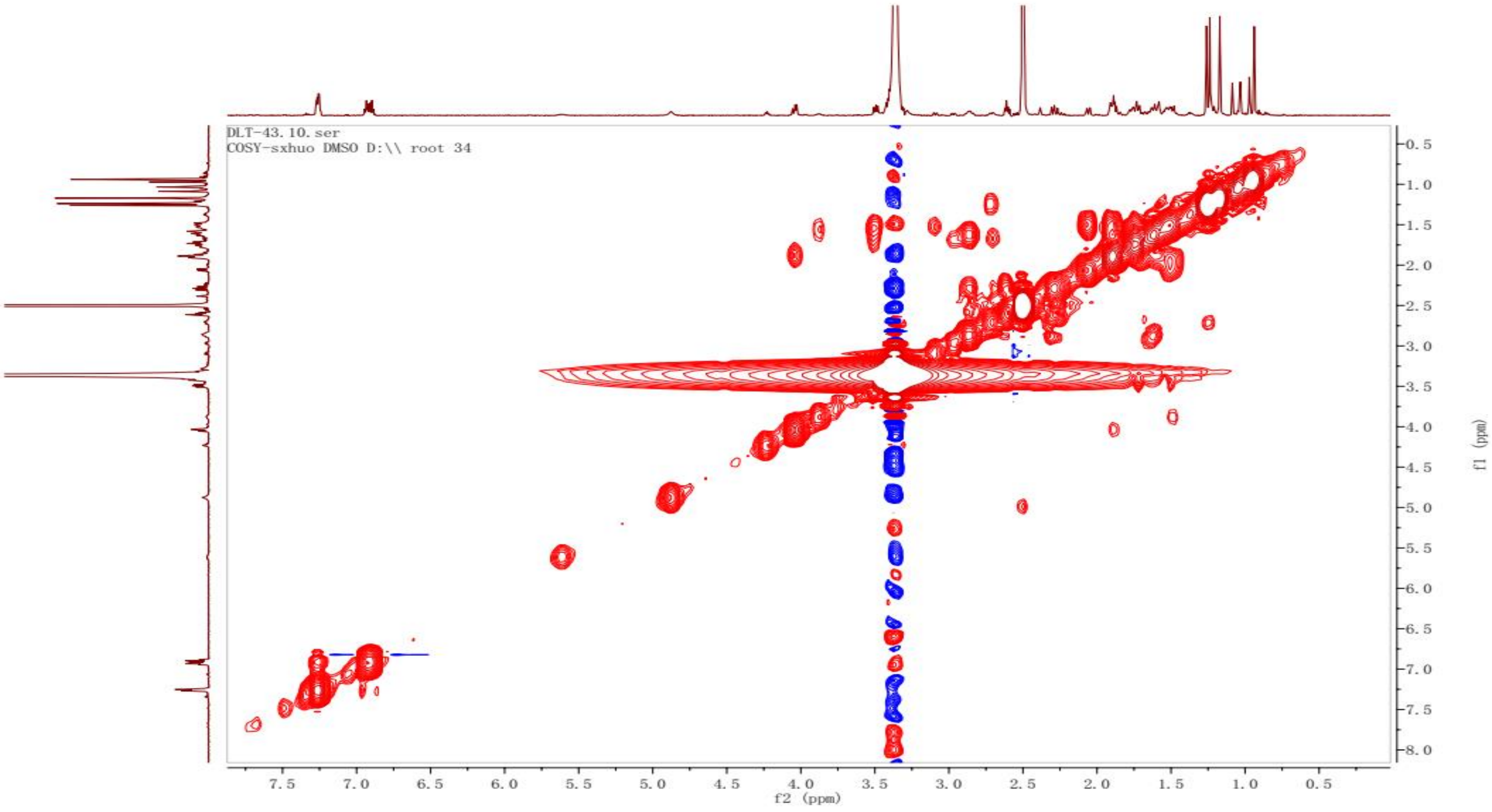

Figure S17. The COSY Spectrum of Compounds **2** in DMSO

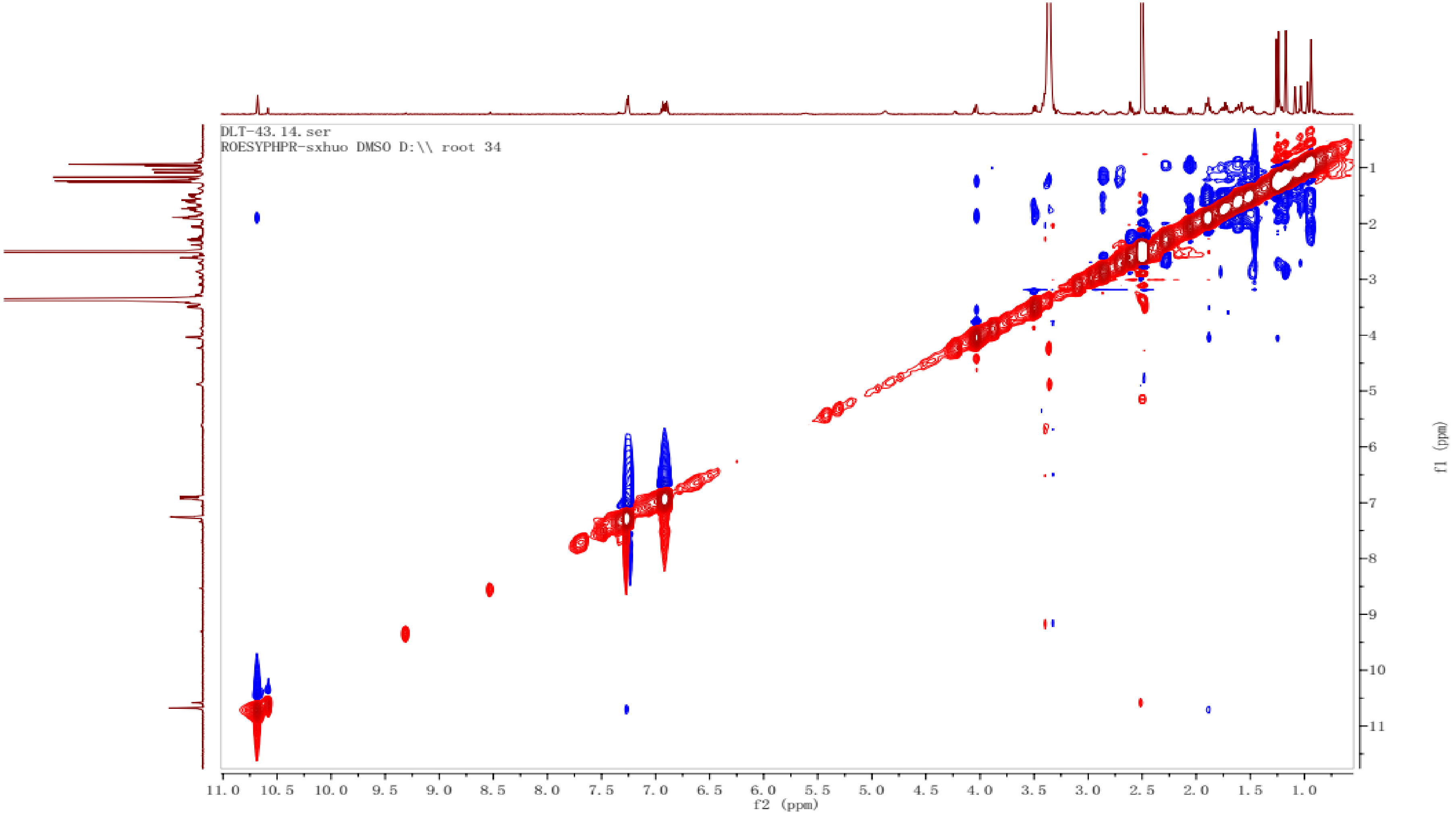

Figure S18. The ROESY Spectrum of Compound **2** in DMSO

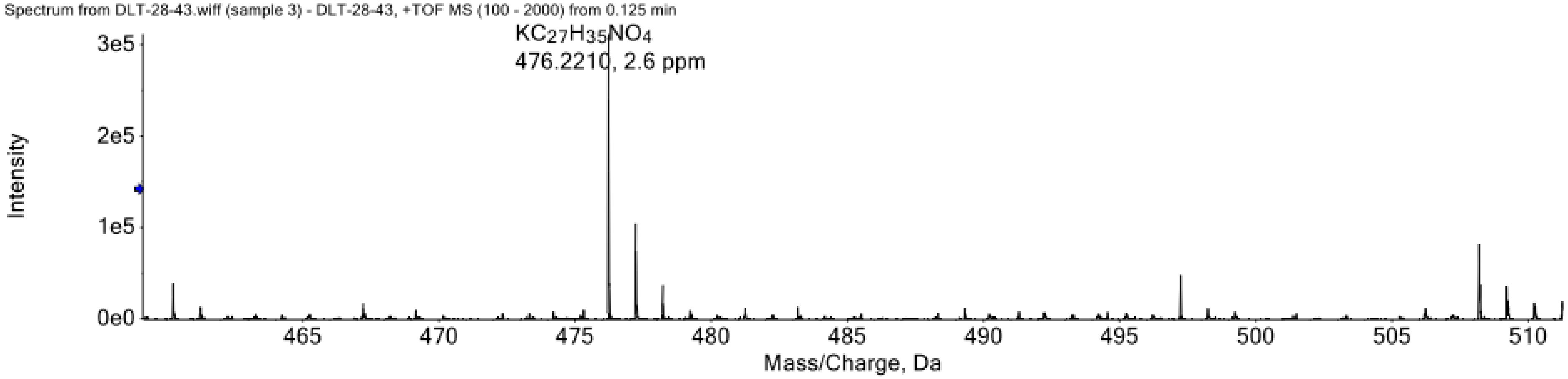

Hit	Formula	m/z	RDB	ppm	MS Rank	MSMS ppm	MSMS Rank	Found
1	C27H35NO4	476.2198	11.0	2.6	1			NA/NA

Figure S19. The HRESIMS Spectroscopic Data of Compounds **2**

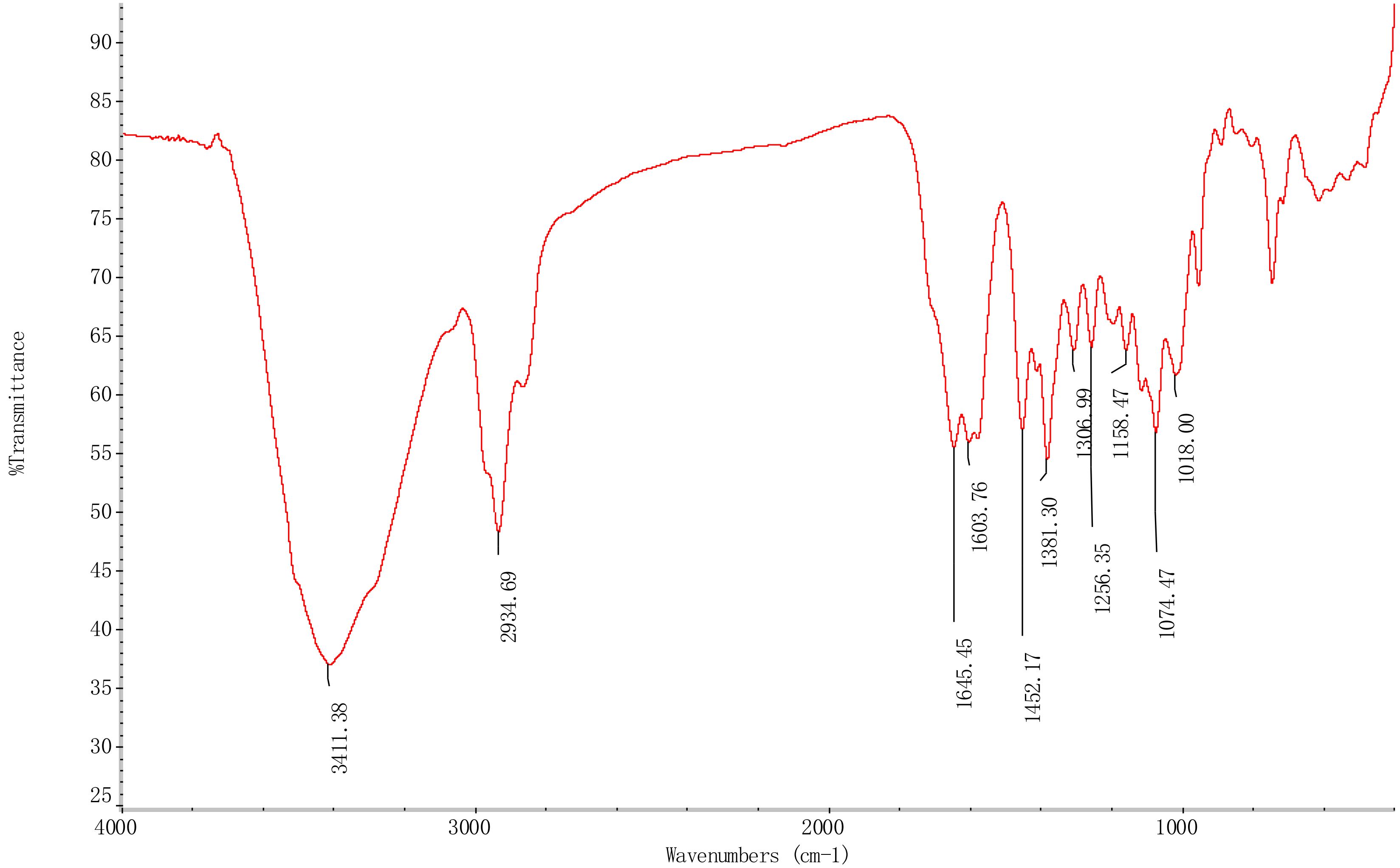

Figure S20. The IR Spectrum of Compounds **2**

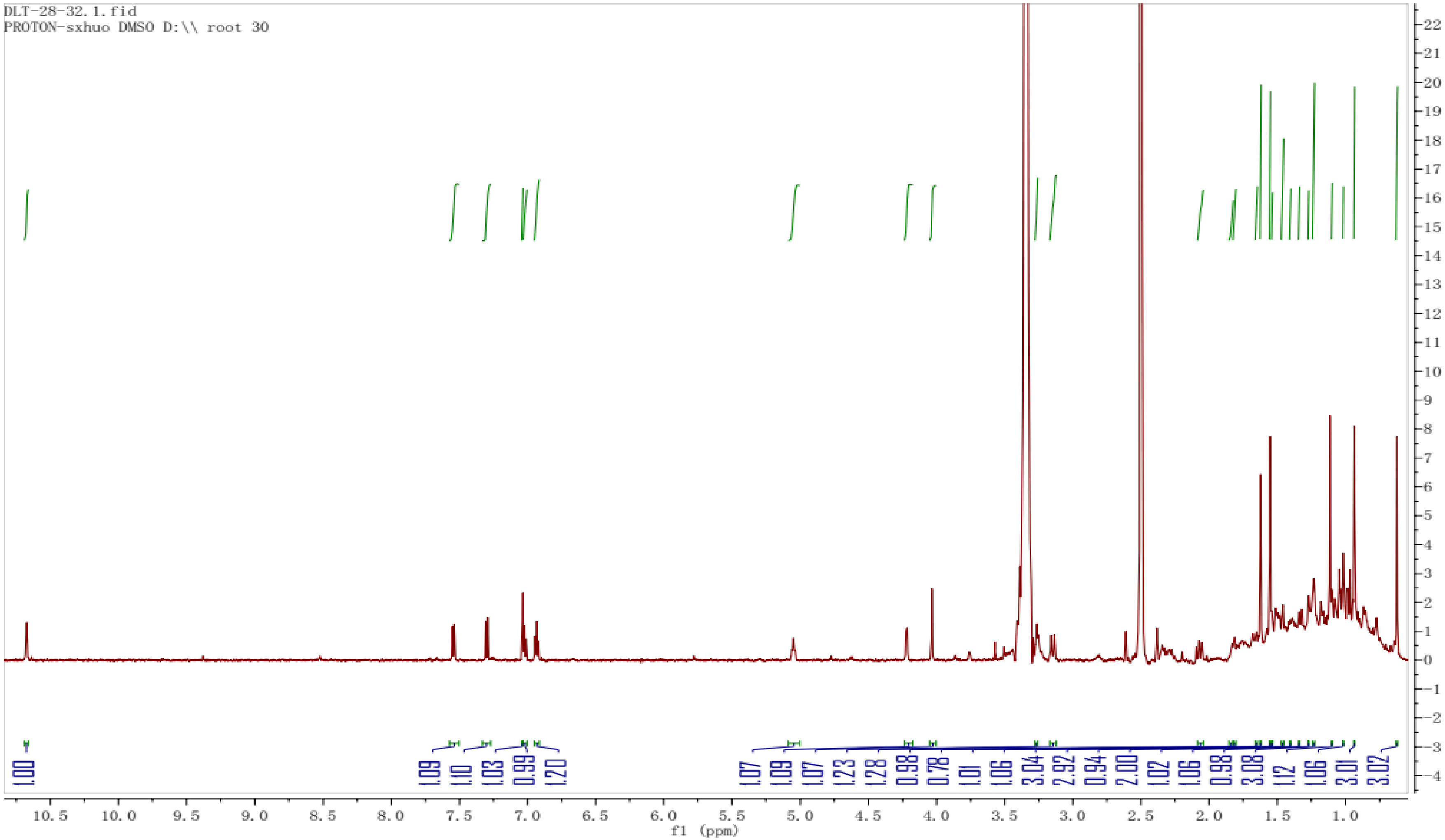

Figure S21. The ^{1}H NMR Spectrum of Compound **3** in DMSO

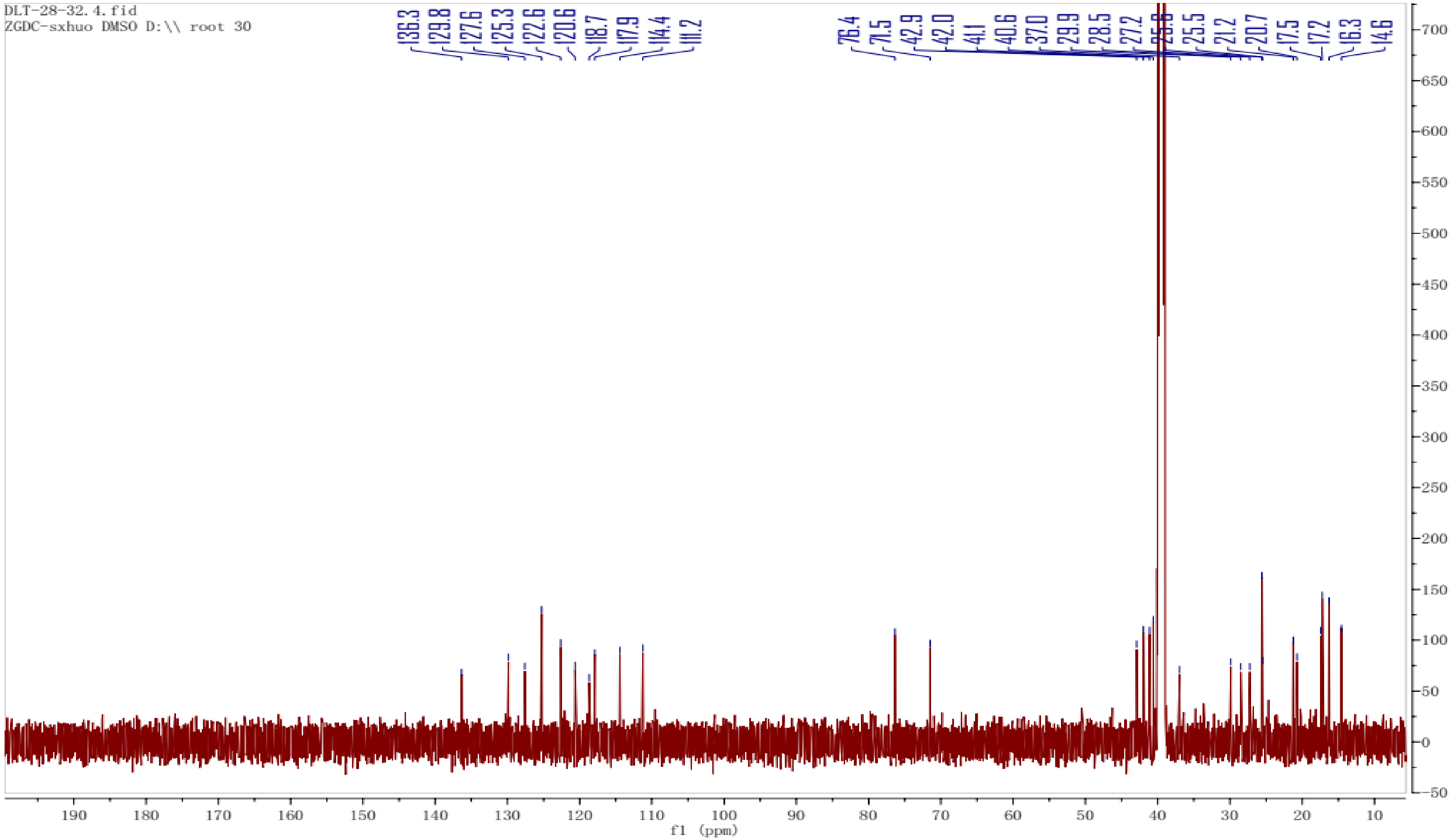

Figure S22. The ^{13}C NMR Spectrum of Compound **3** in DMSO

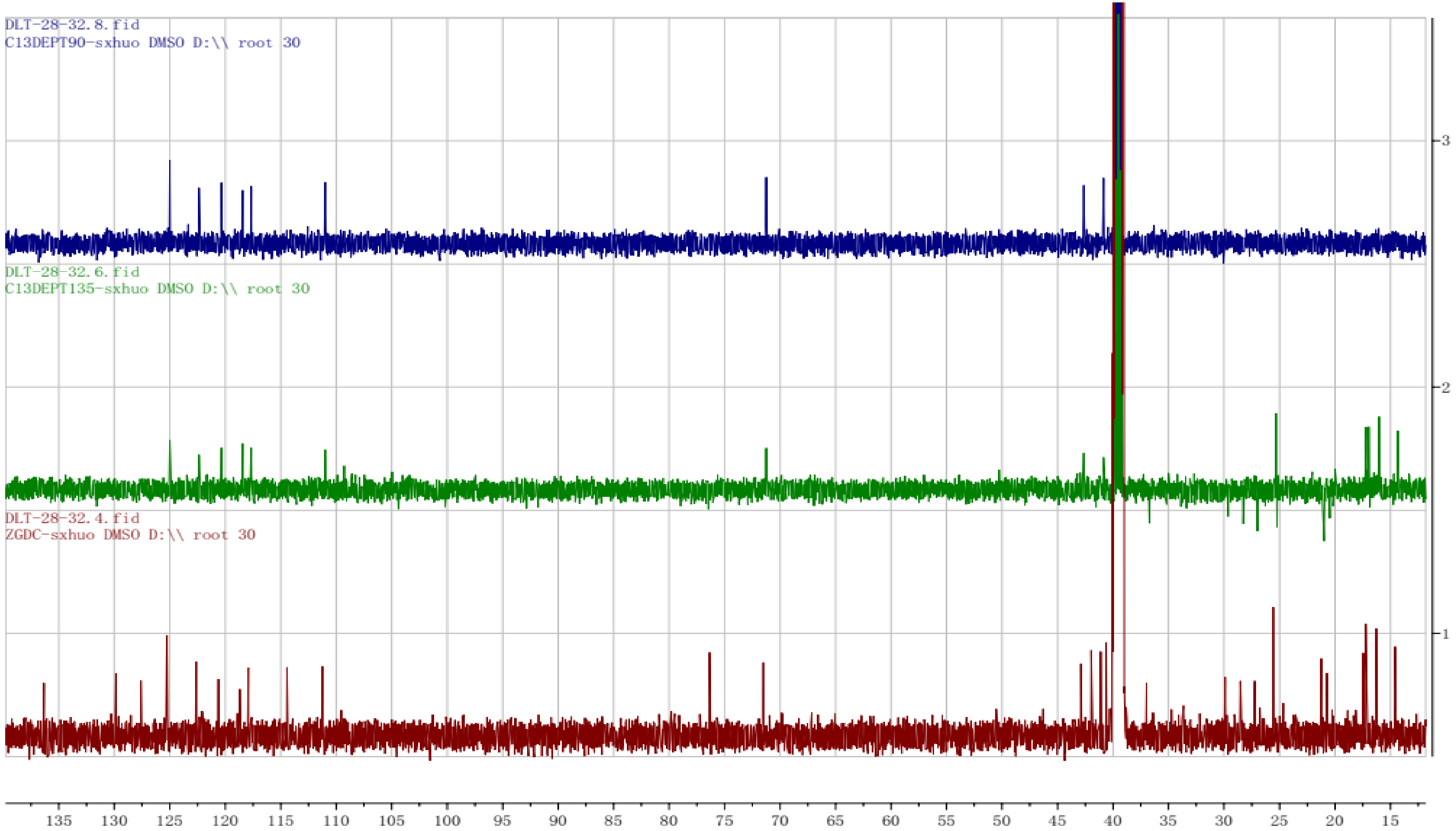

Figure S23. The DEPT Spectrum of Compound **3** in DMSO

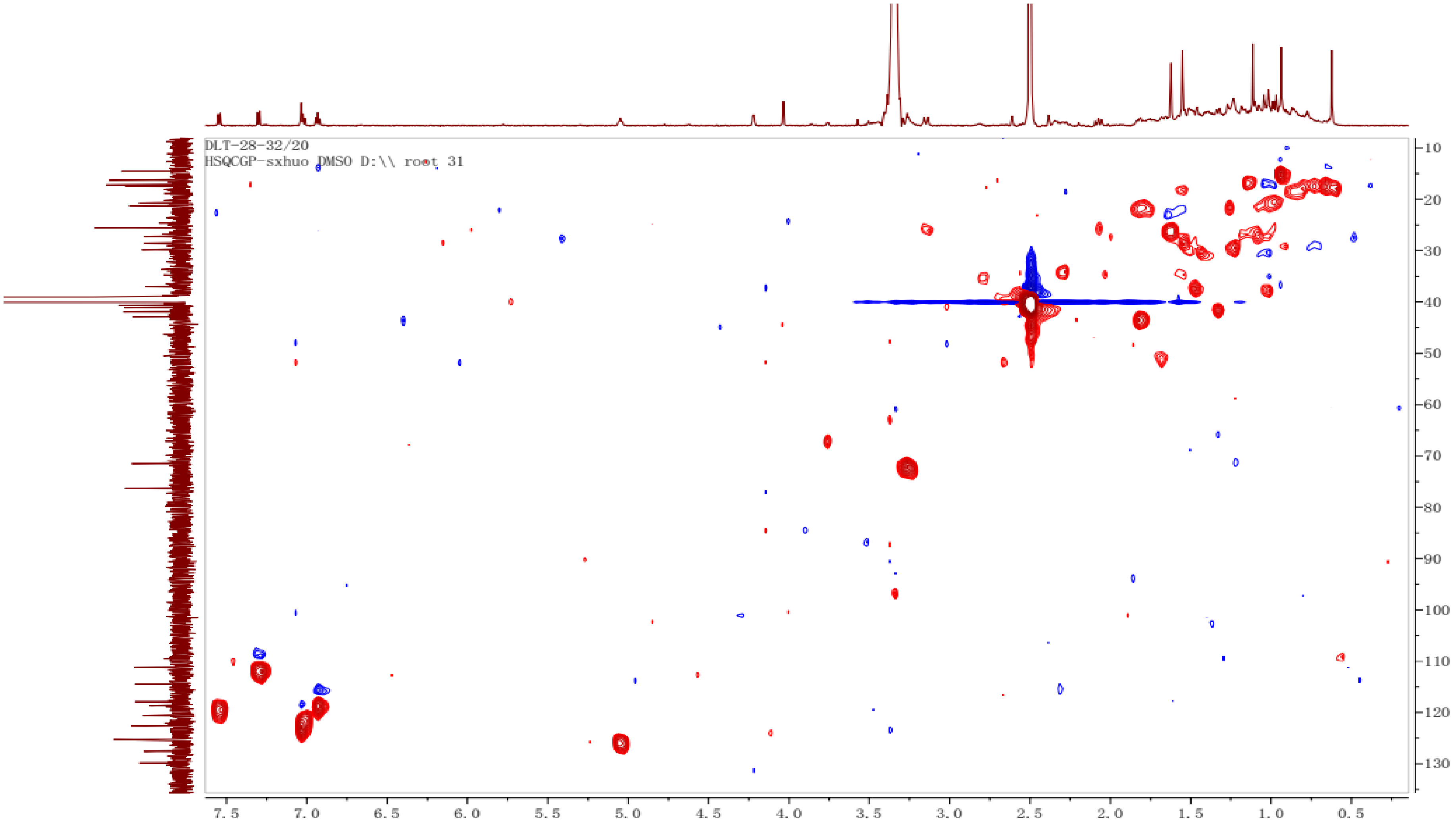

Figure S24. The HMQC Spectrum of Compound **3** in DMSO

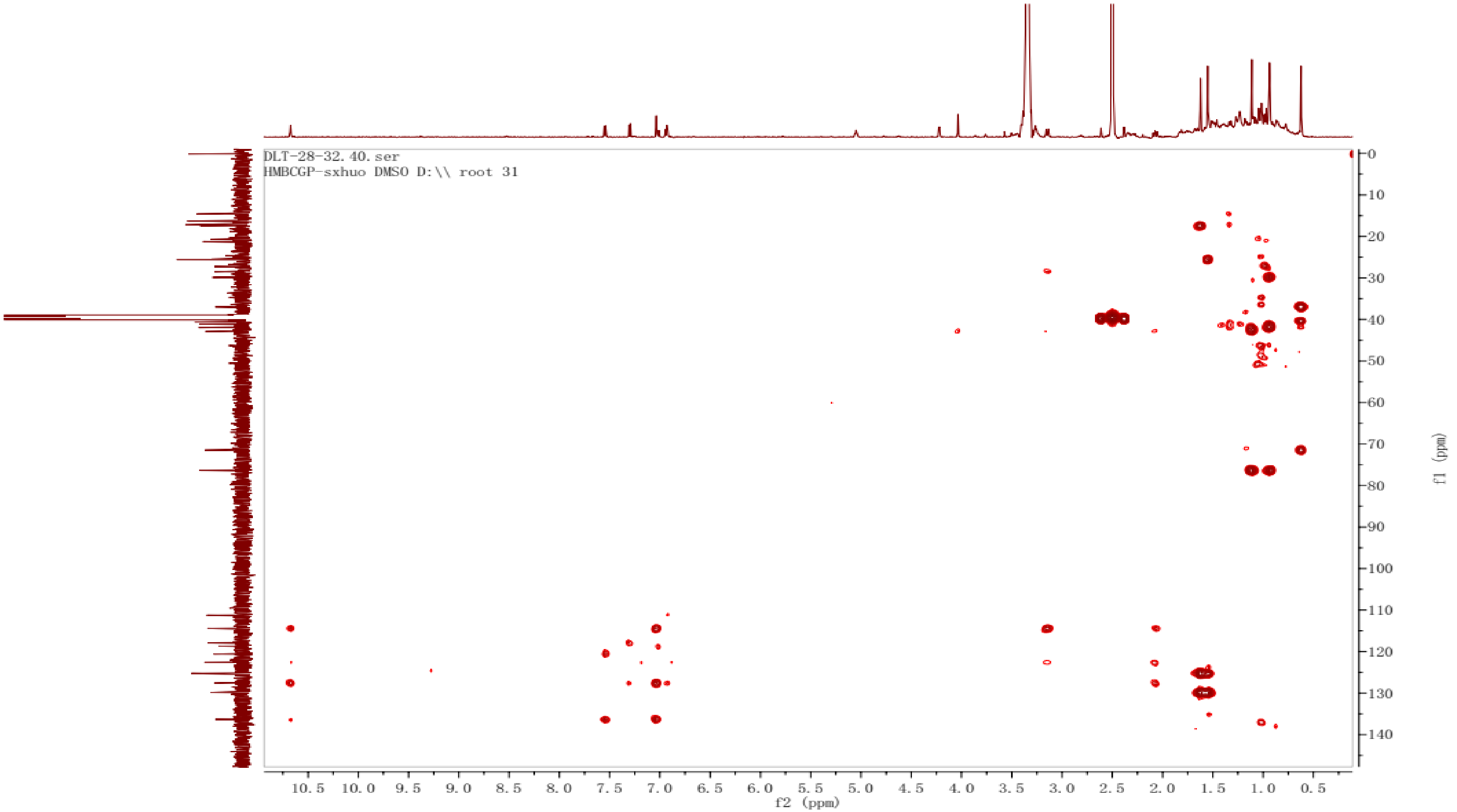

Figure S25. The HMBC Spectrum of Compound **3** in DMSO

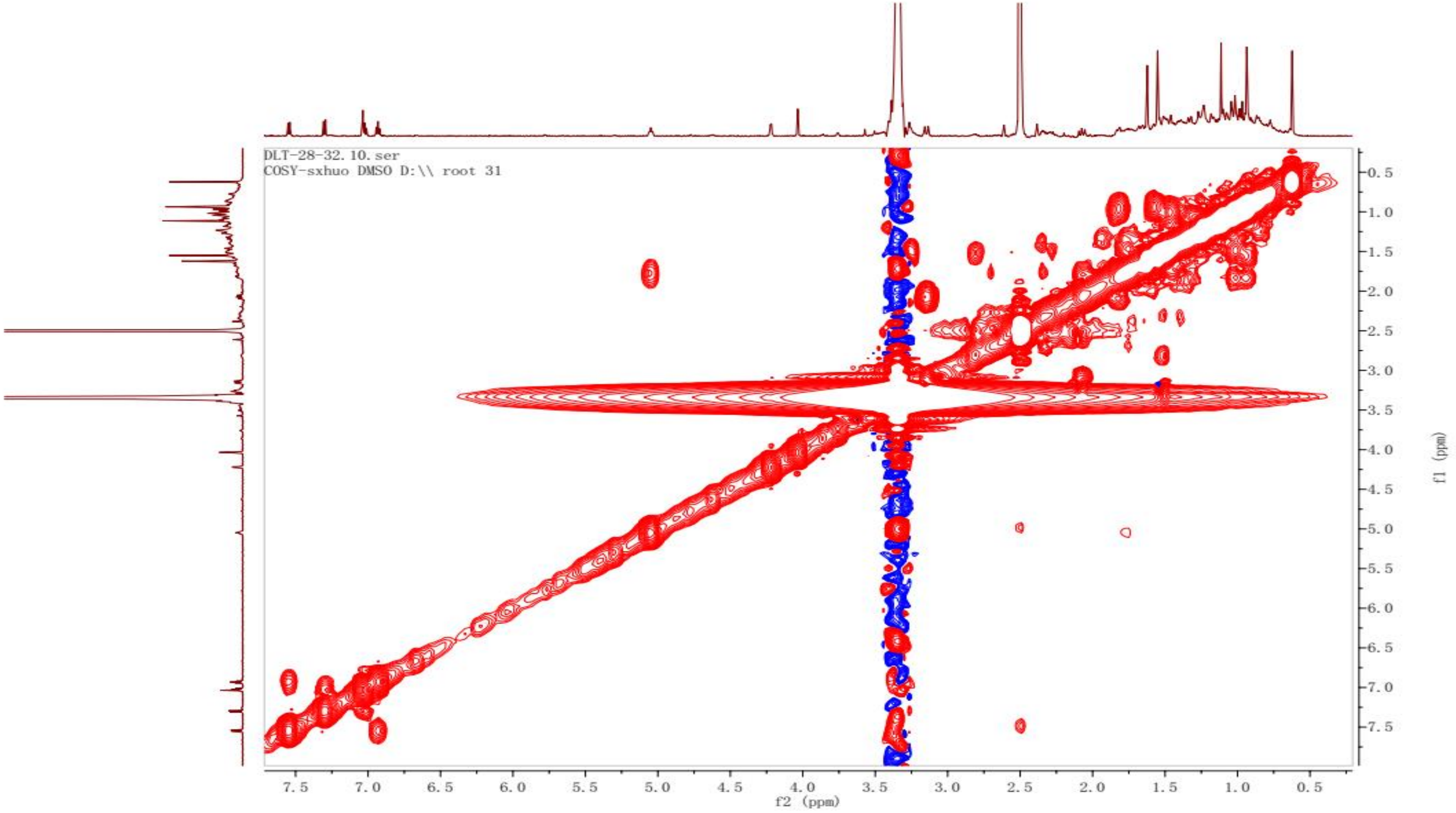

Figure S26. The COSY Spectrum of Compound **3** in DMSO

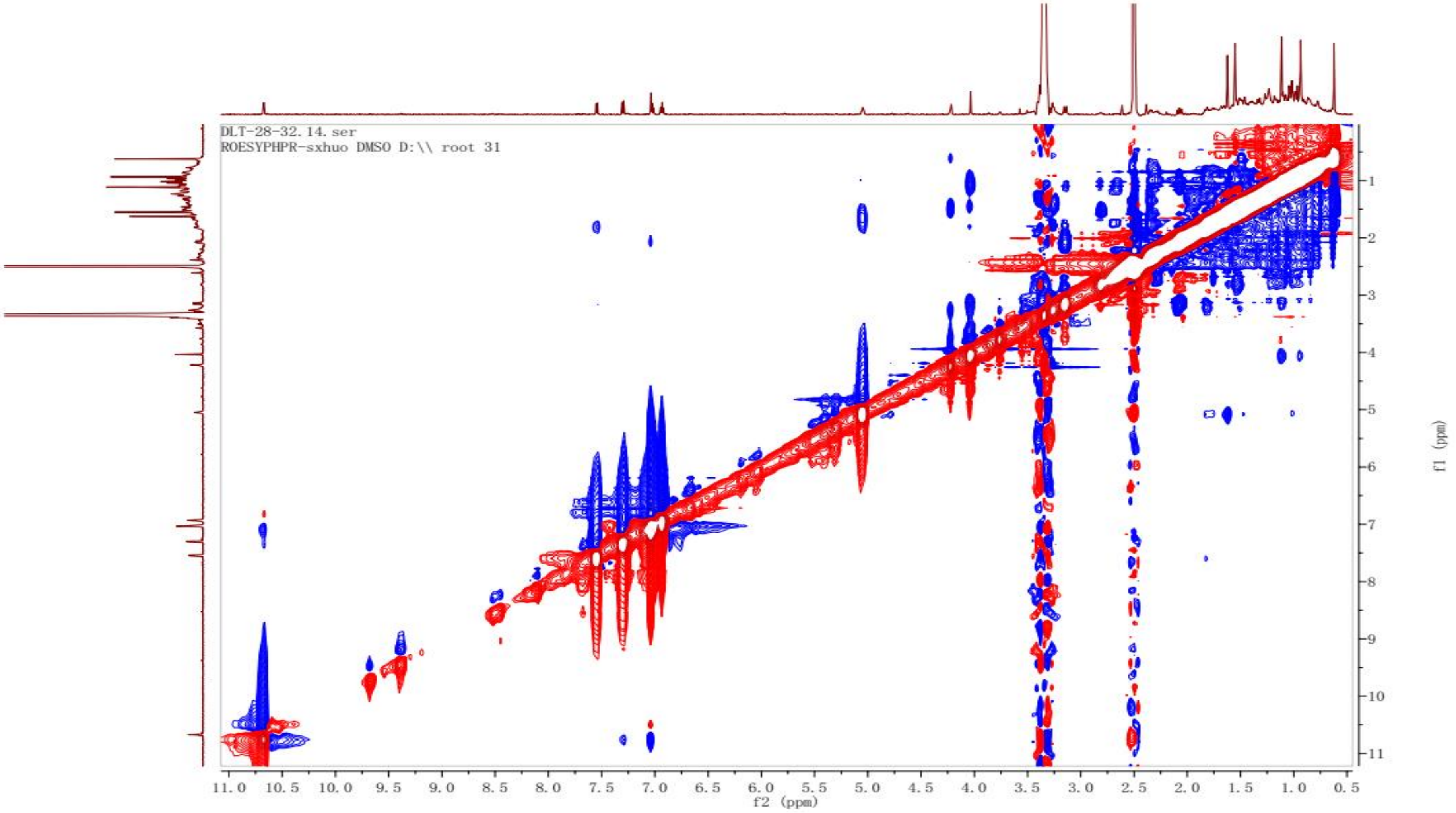

Figure S27. The ROESY Spectrum of Compound **3** in DMSO

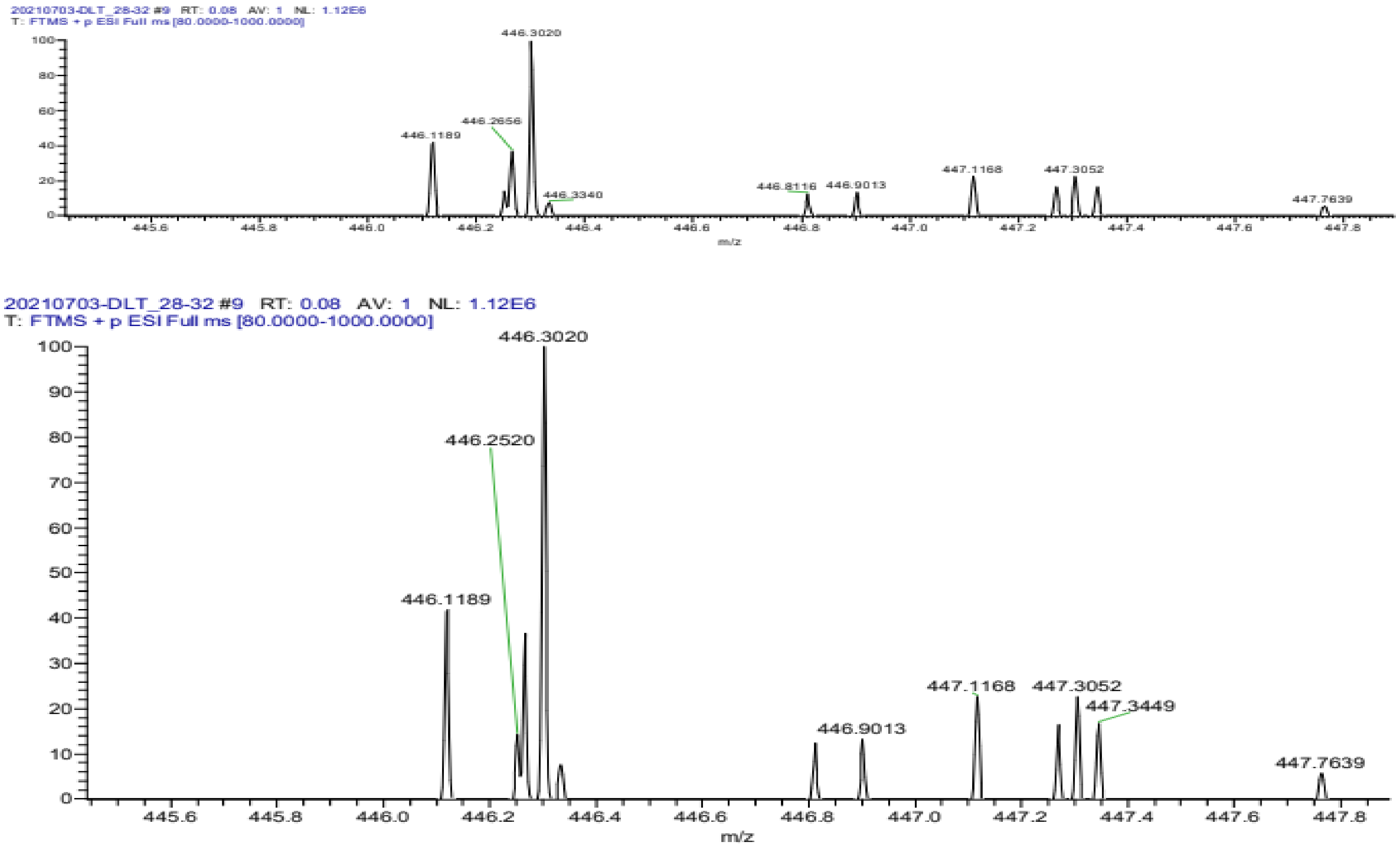

Figure S28. The HRESIMS Spectroscopic Data of Compound **3**

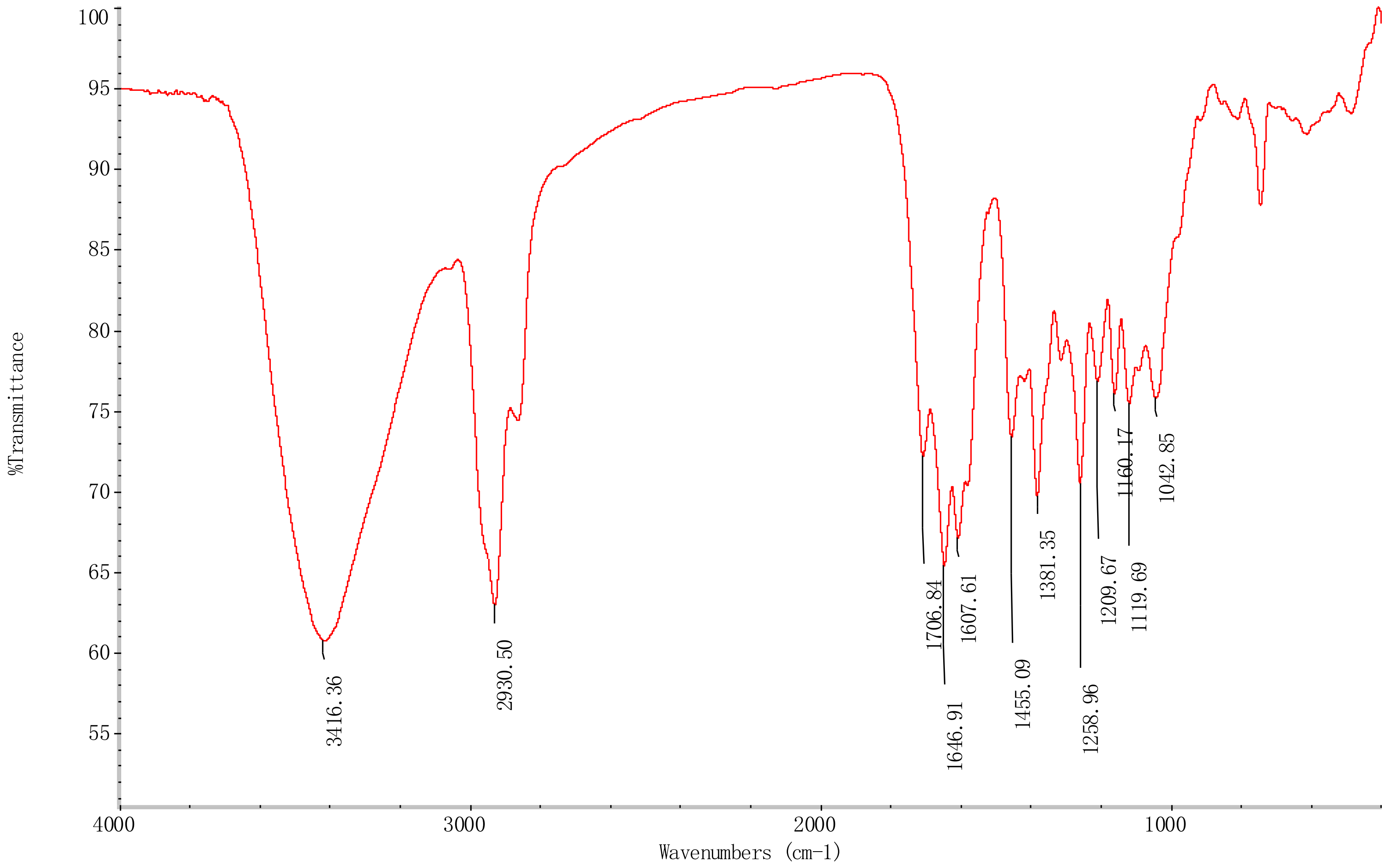

Figure S29. The IR Spectrum of Compound **3**

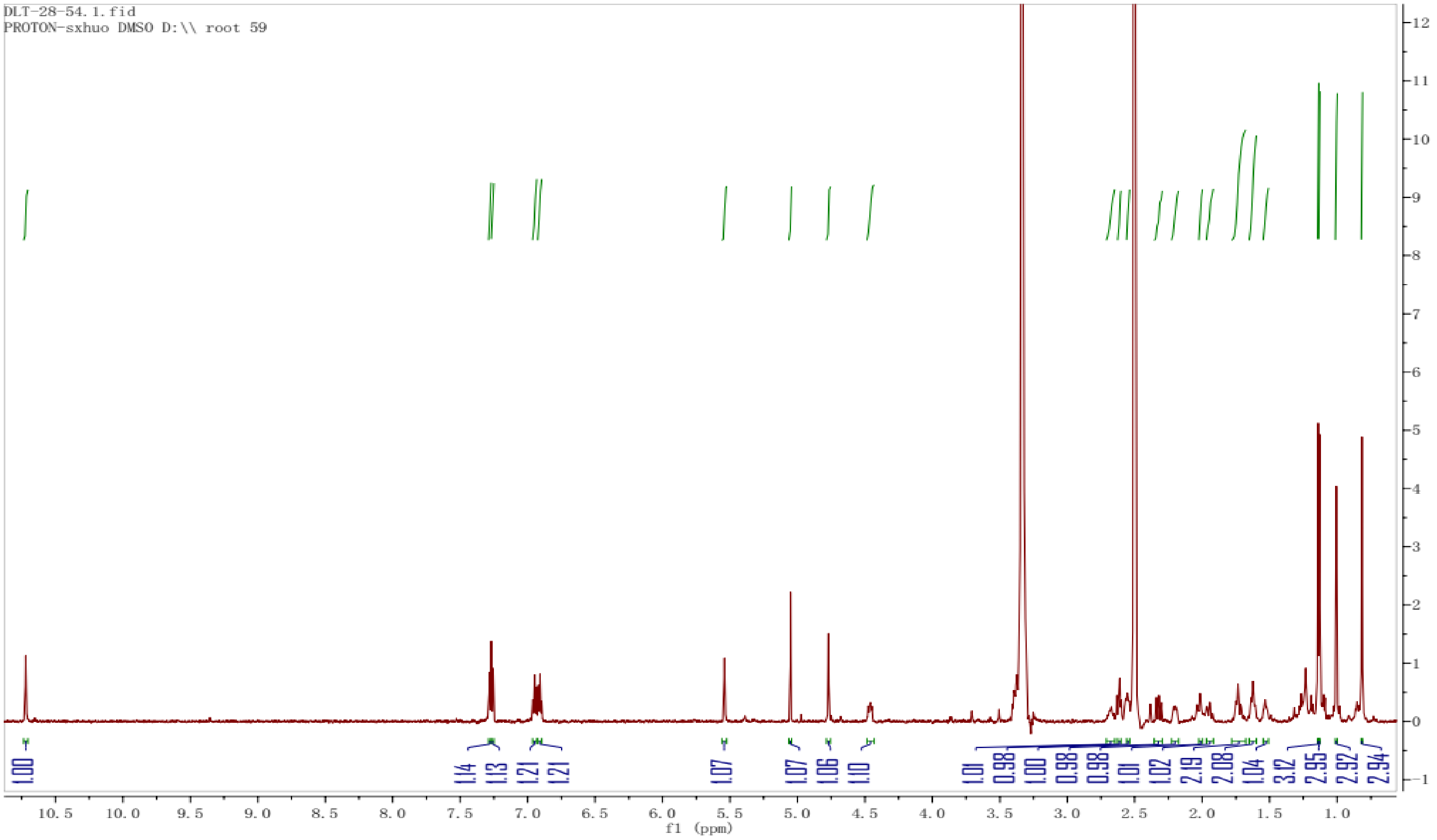

Figure S30. The ^{1}H NMR Spectrum of Compound **4** in DMSO

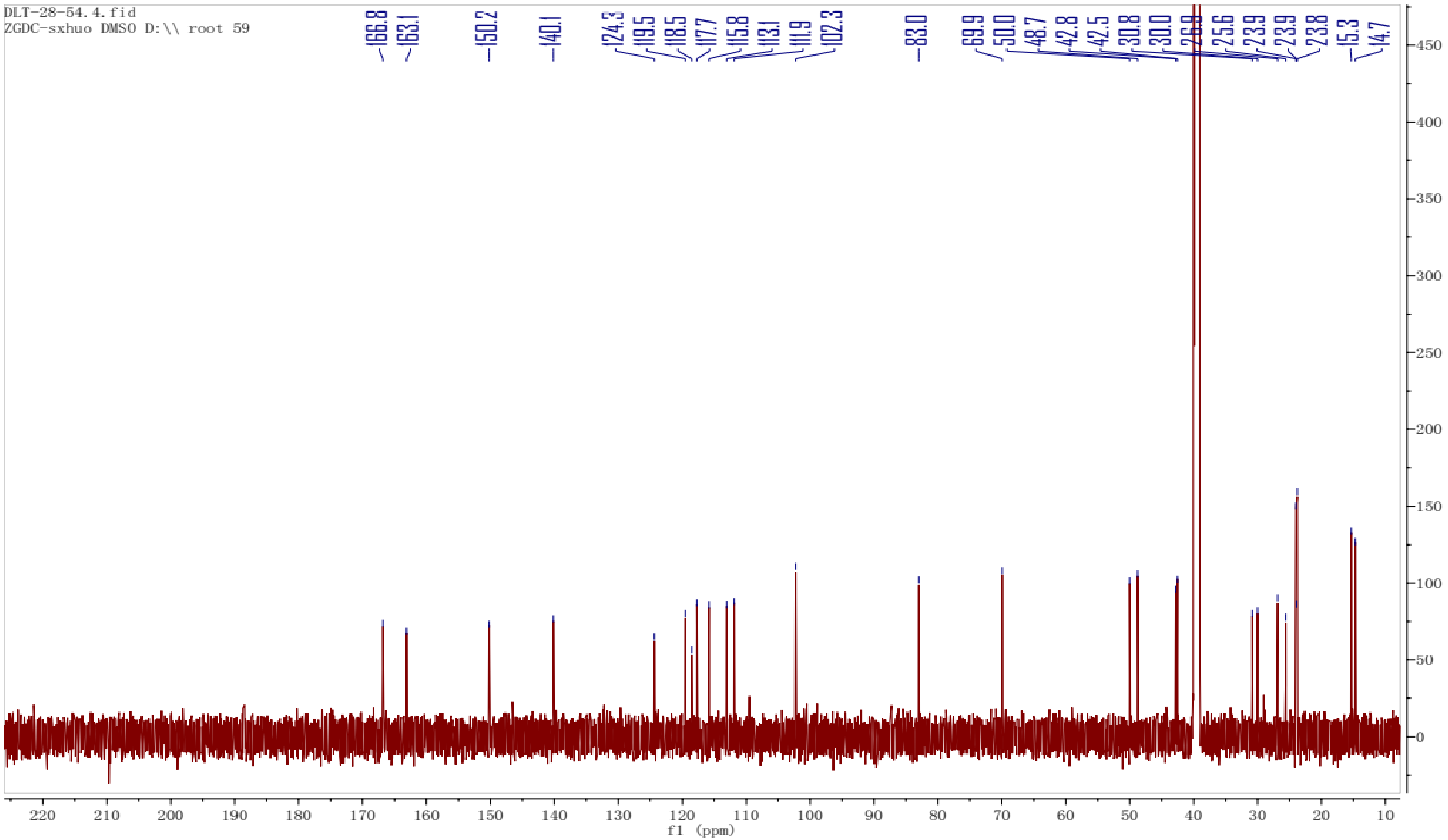

Figure S31. The ^{13}C NMR Spectrum of Compound **4** in DMSO

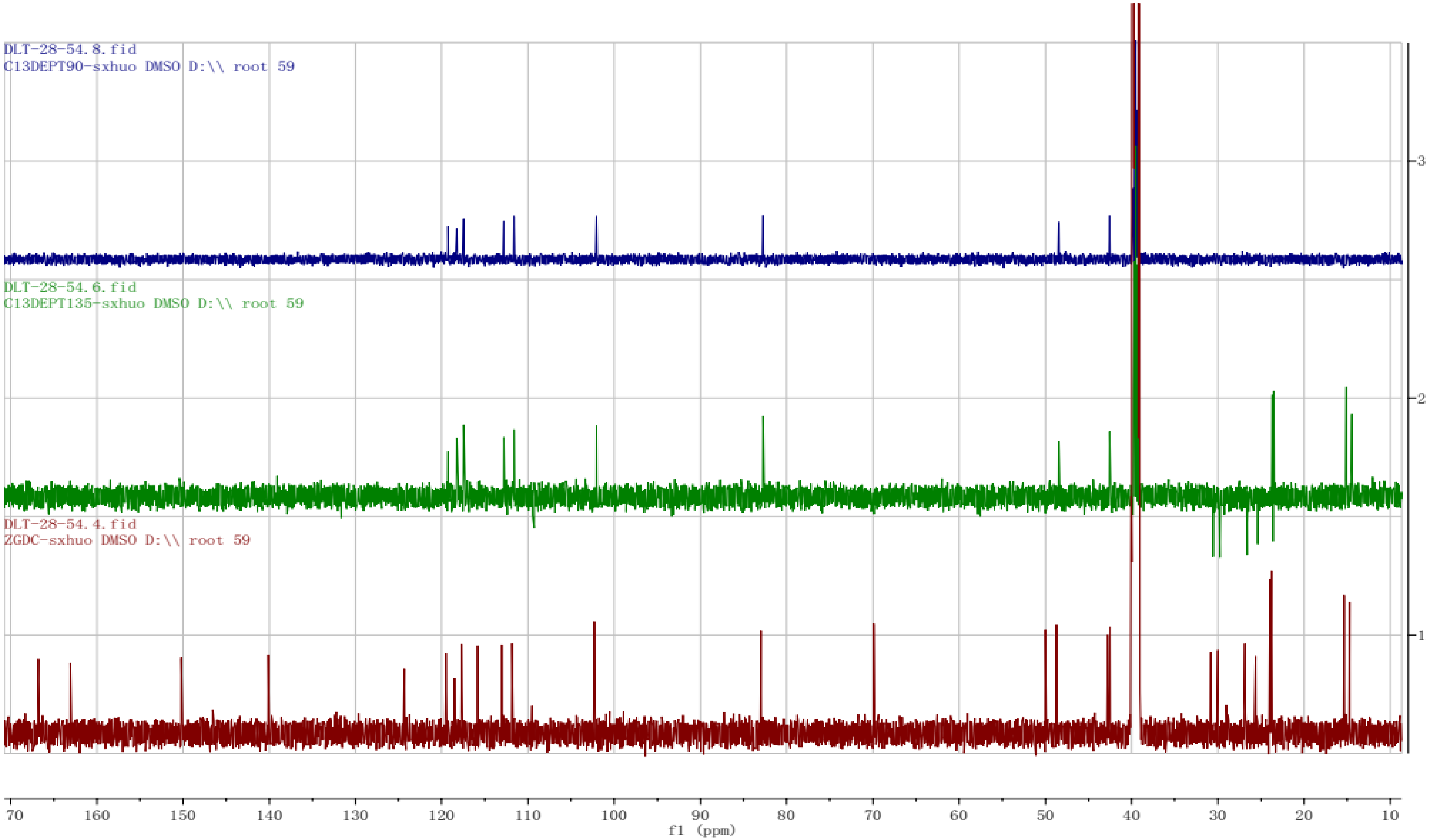

Figure S32. The DEPT Spectrum of Compound **4** in DMSO

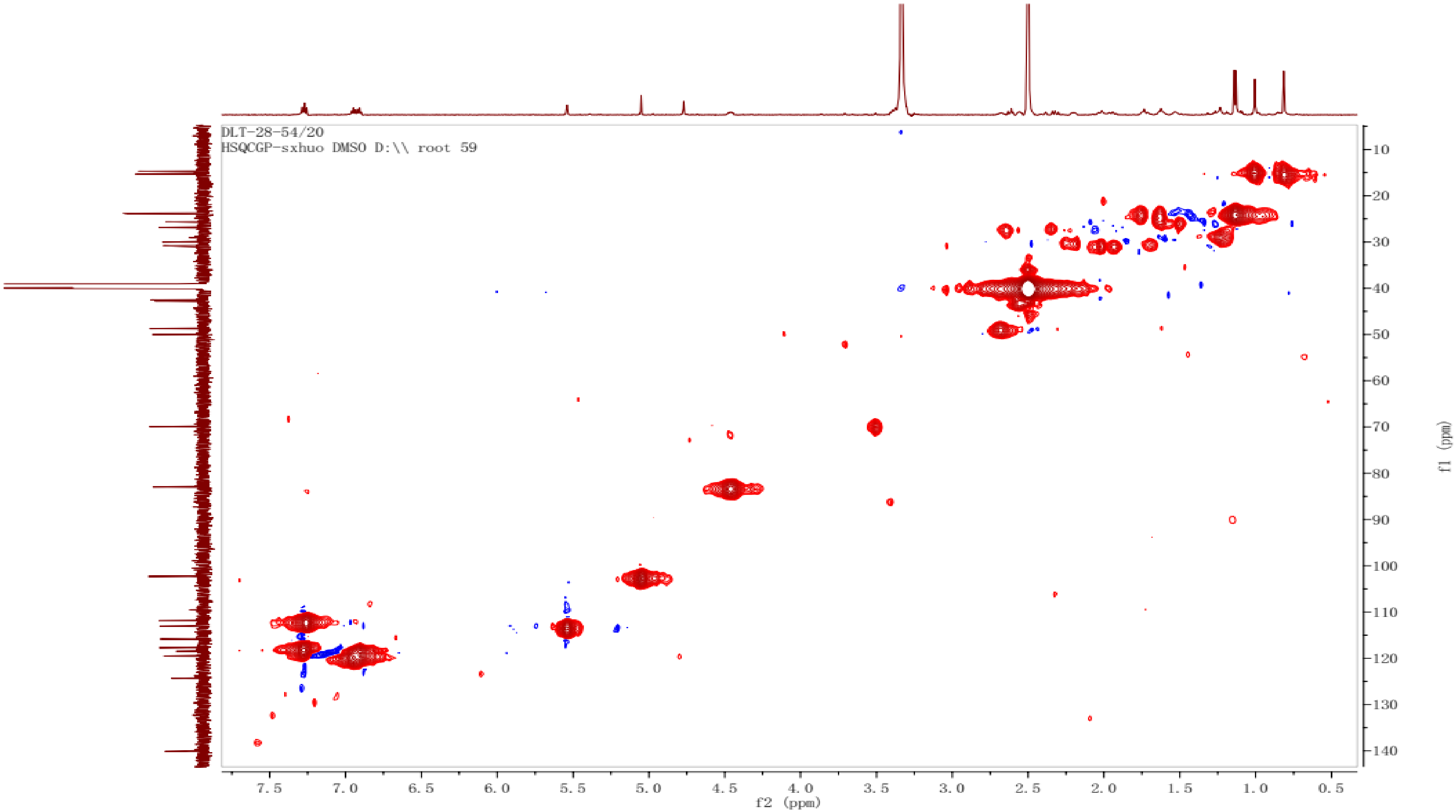

Figure S33. The HMQC Spectrum of Compound **4** in DMSO

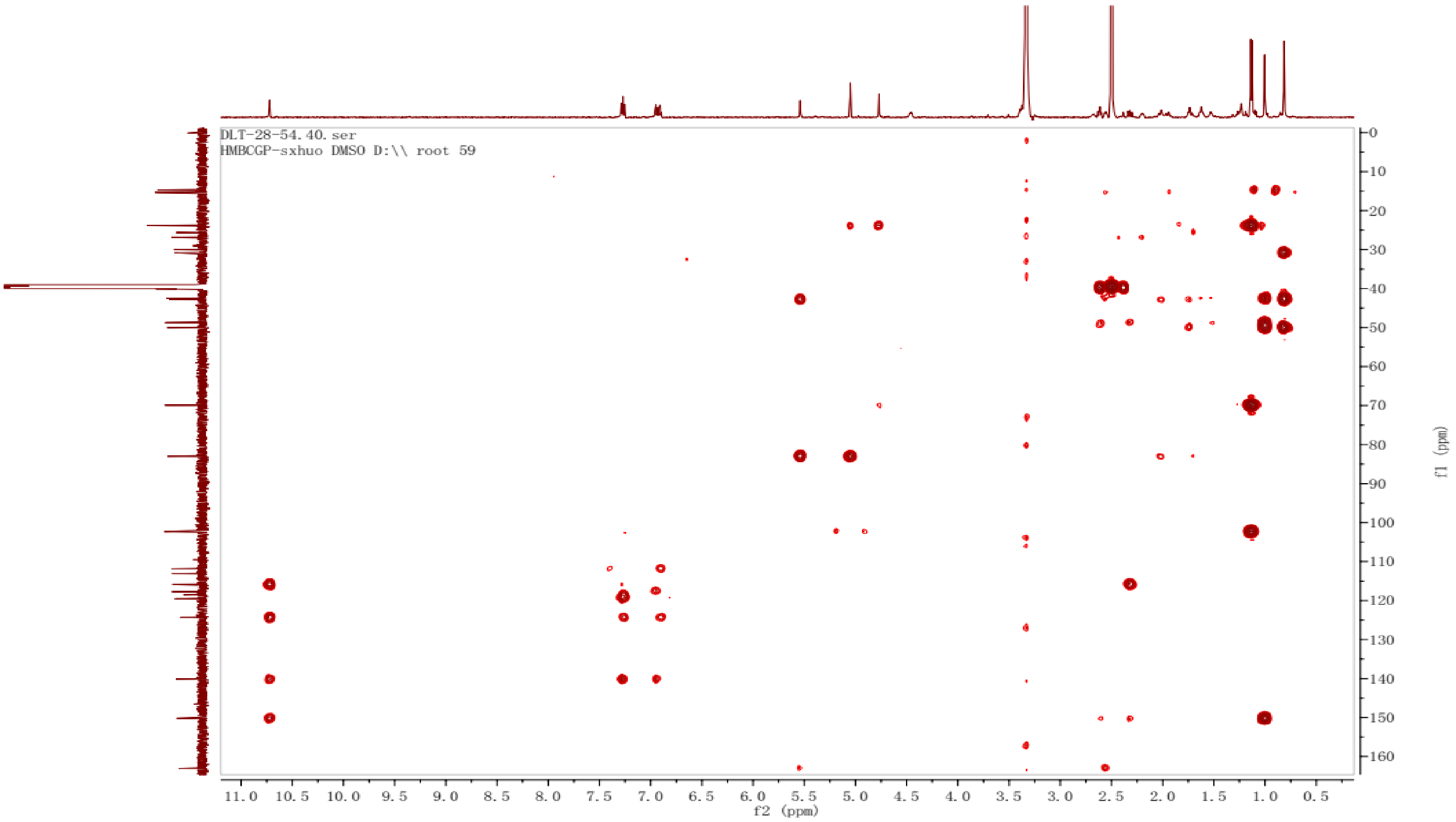

Figure S34. The HMBC Spectrum of Compound **4** in DMSO

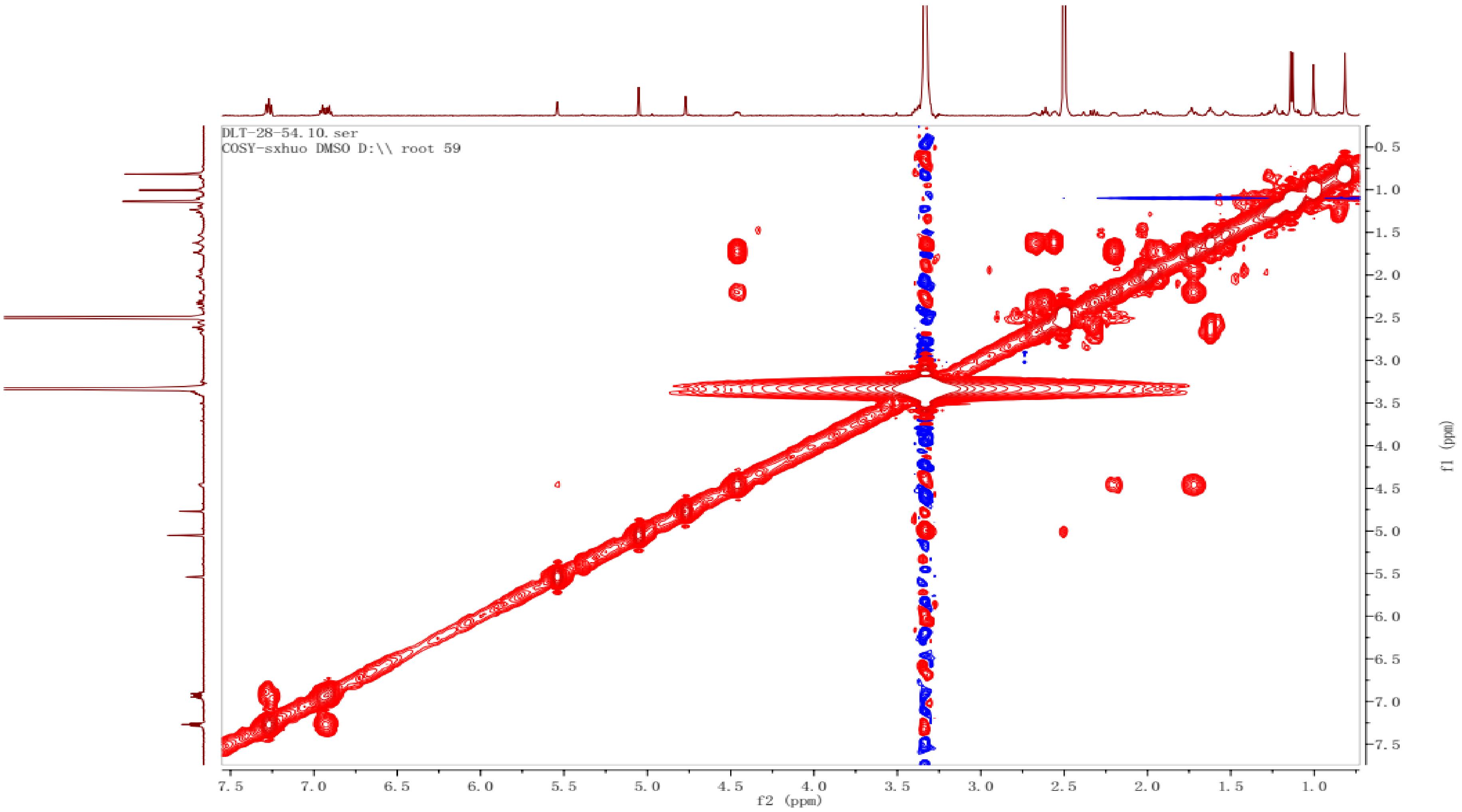

Figure S35. The COSY Spectrum of Compound **4** in DMSO

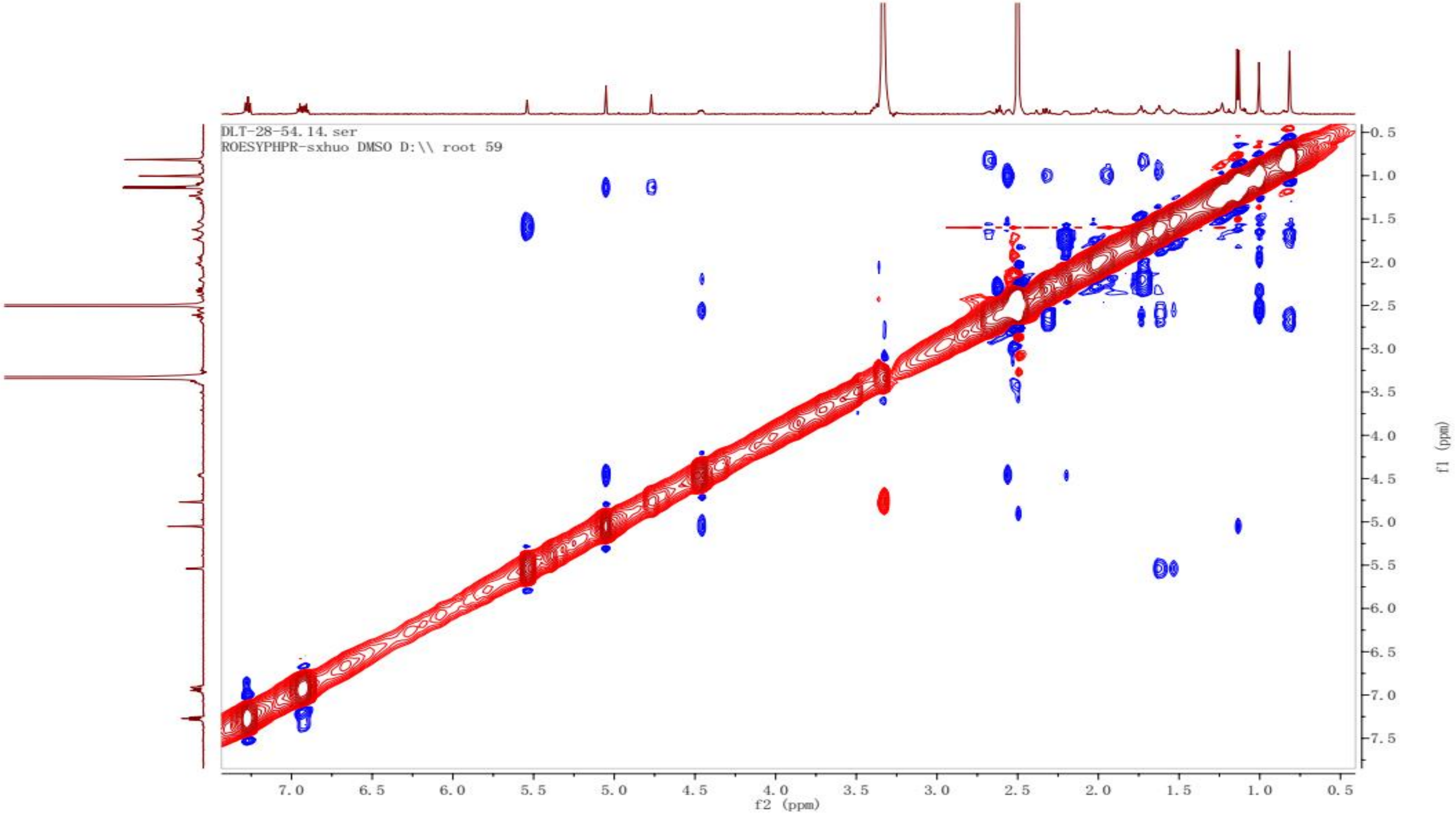

Figure S36. The ROESY Spectrum of Compound **4** in DMSO

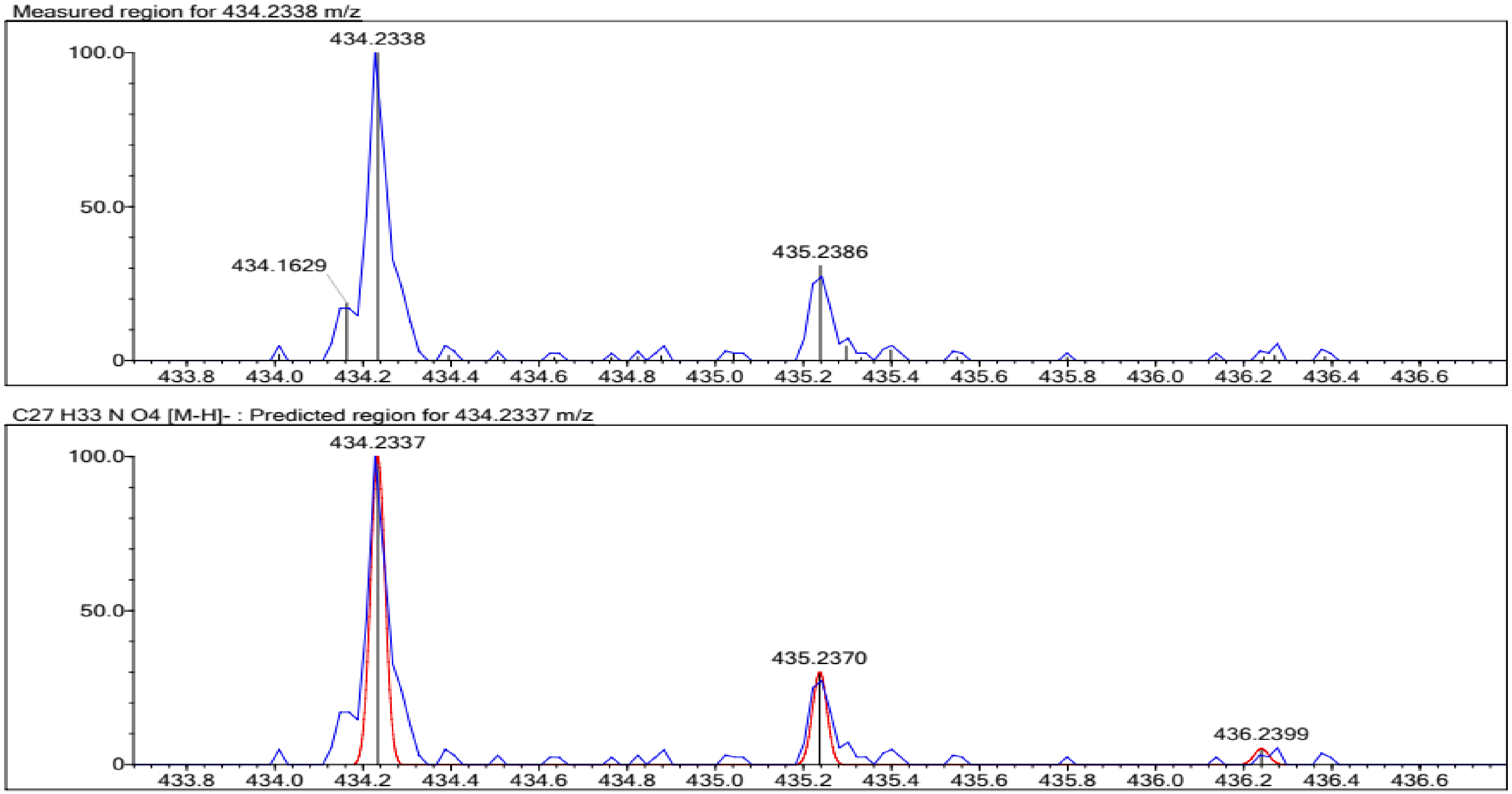

Formula (M)	Ion	Meas. m/z	Pred. m/z	Df. (mDa)	Df. (ppm)	DBE
C27 H33 N O4	[M-H]-	434.2338	434.2337	0.1	0.23	12.0

Figure S37. The HRESIMS Spectroscopic Data of Compound **4**

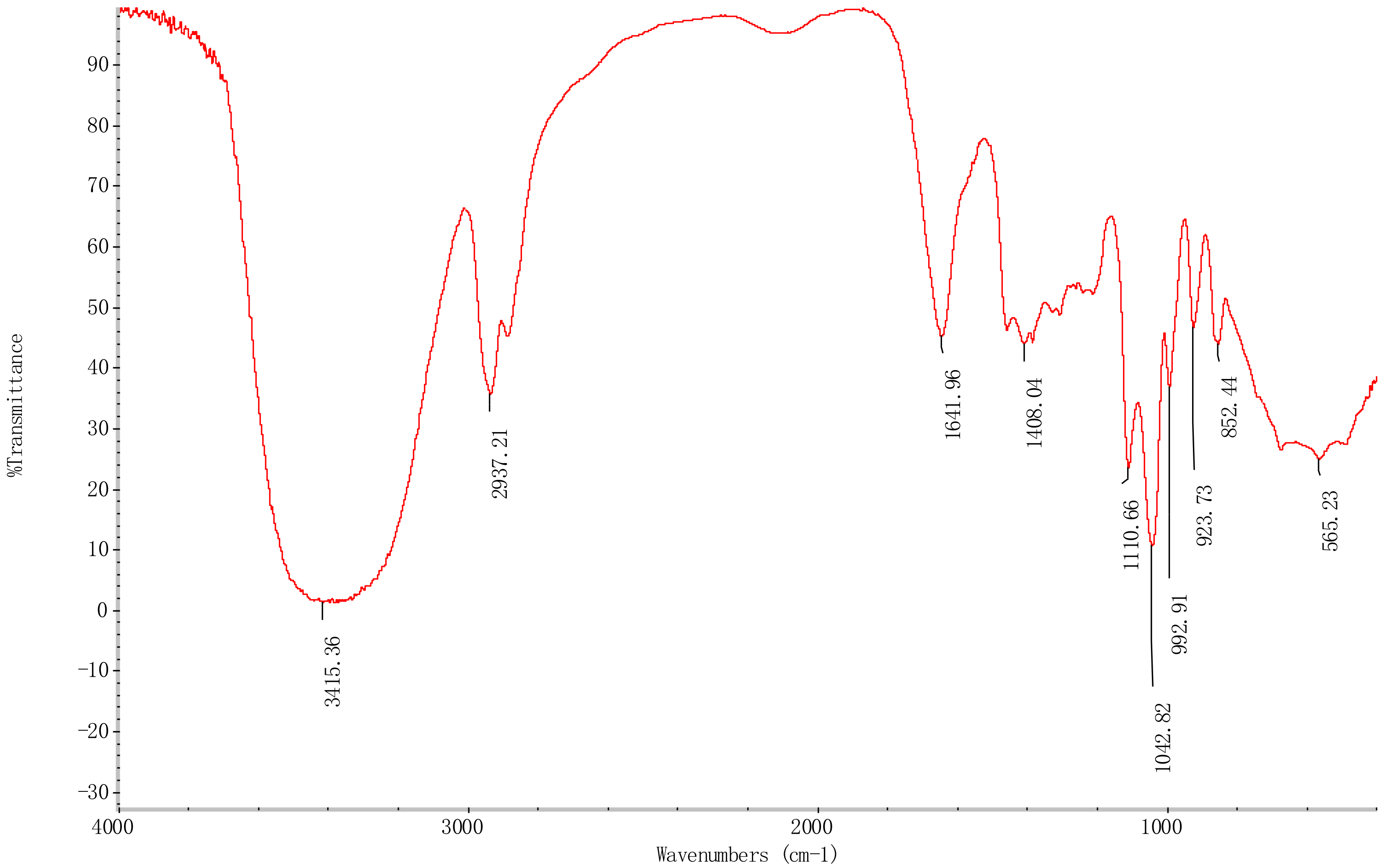

Figure S38. The IR Spectrum of Compound **4**

The strain of *Penicillium* sp. KFD28

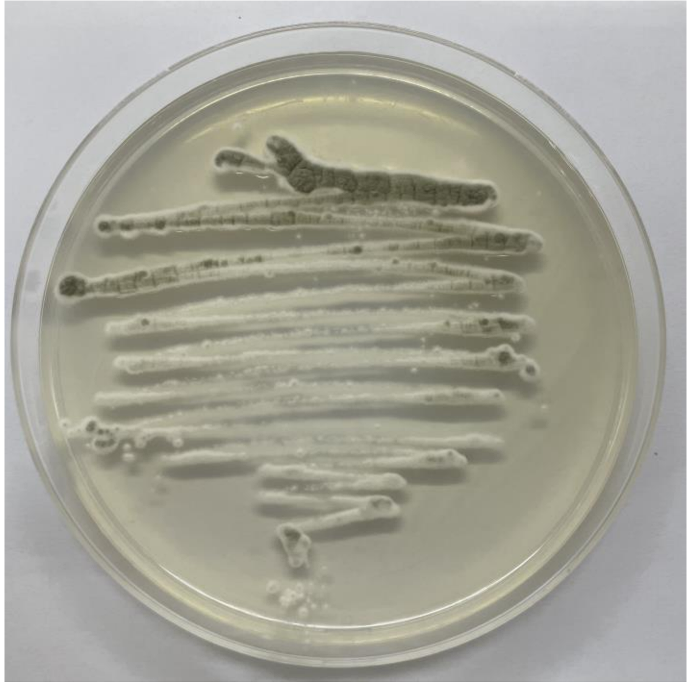

Figure S39. The picture of strain *Penicillium* sp. KFD28

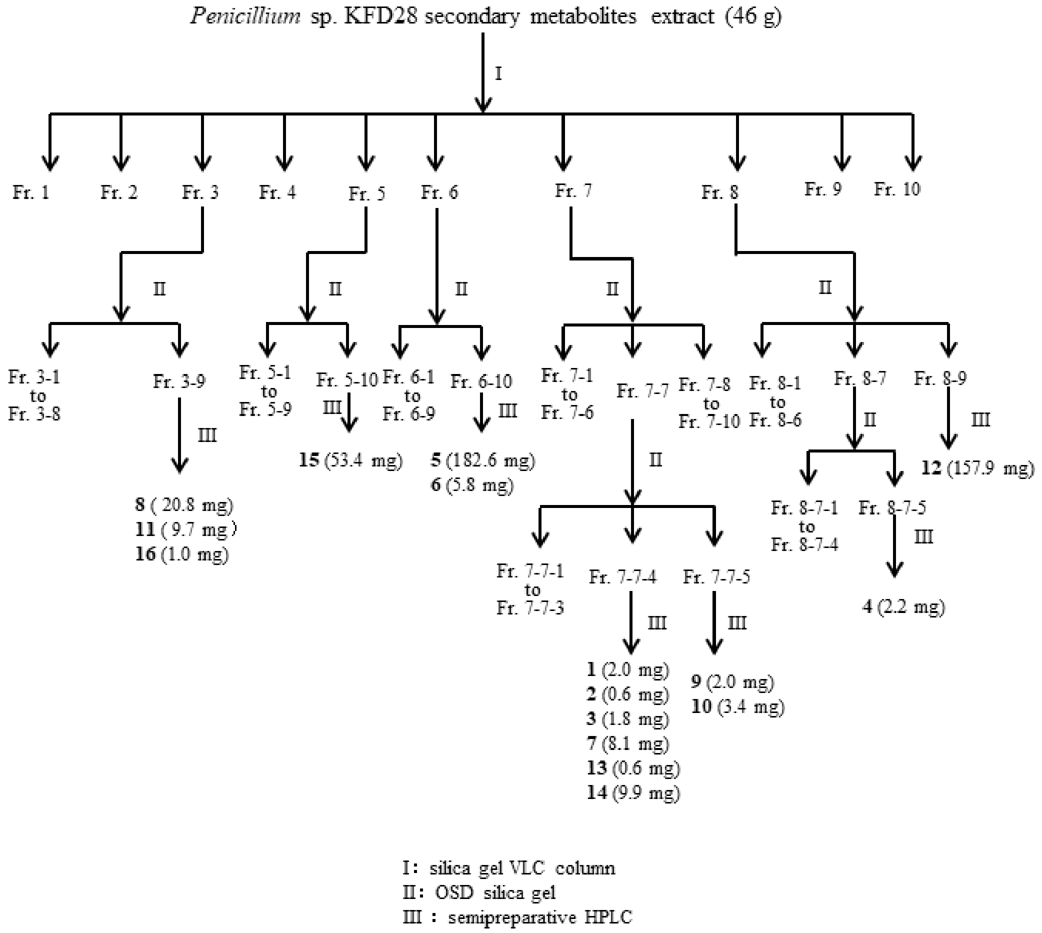

Figure S40. Separation flow chart of *Penicillium* sp. KFD28 secondary metabolites extract

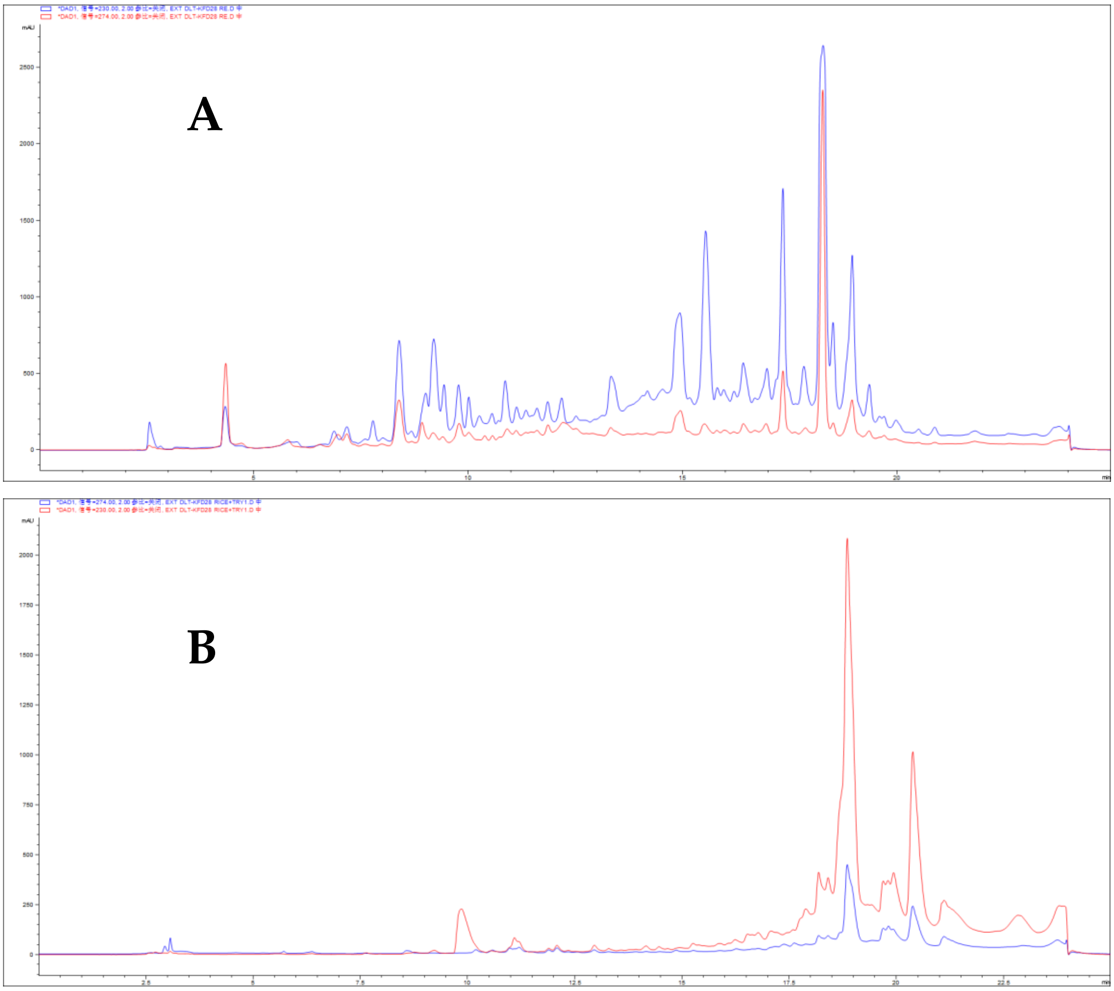

Figure S41. HPLC chromatograms of the EtOAc extracts monitored at wavelengths of 230 nm (blue line) and 274 nm (red line). (A) Liquid medium; (B) Solid rice medium with L-tryptophan.

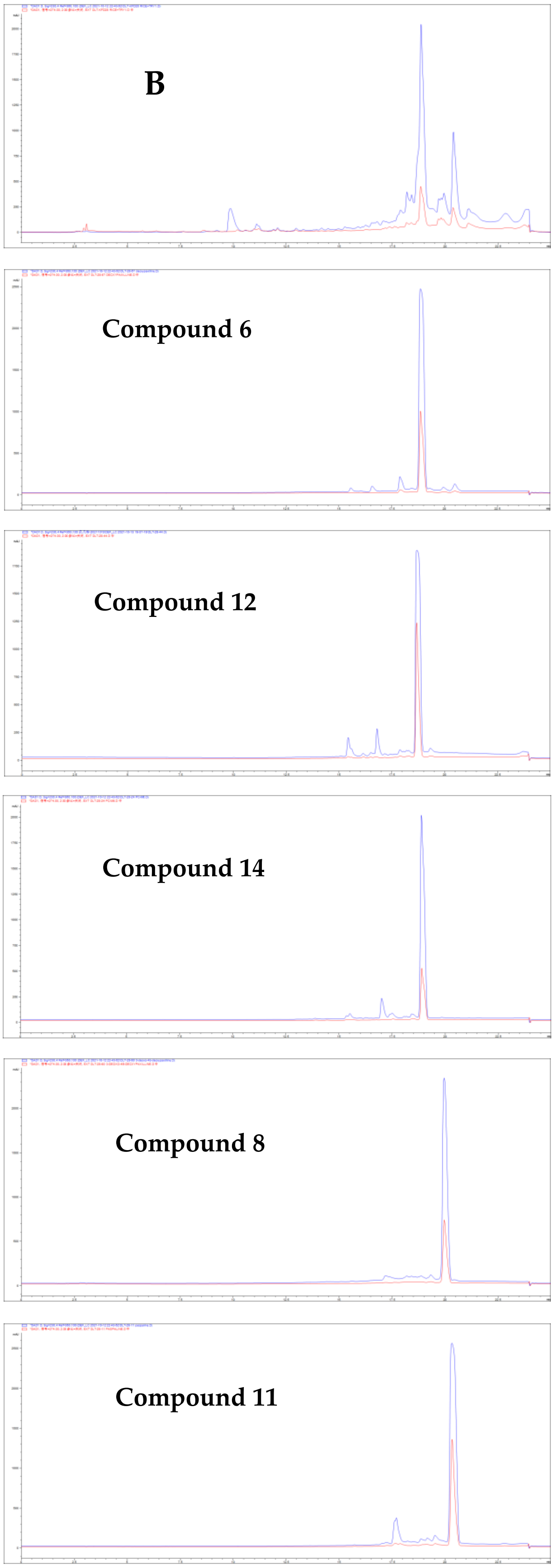

Figure S42. HPLC chromatograms of the EtOAc extracts using Solid rice medium with L-tryptophan, and compounds **6**, **12**, **14, 8** and **11** monitored at wavelengths of 230 nm (blue line) and 274 nm (red line)

ECD curve for compound 4

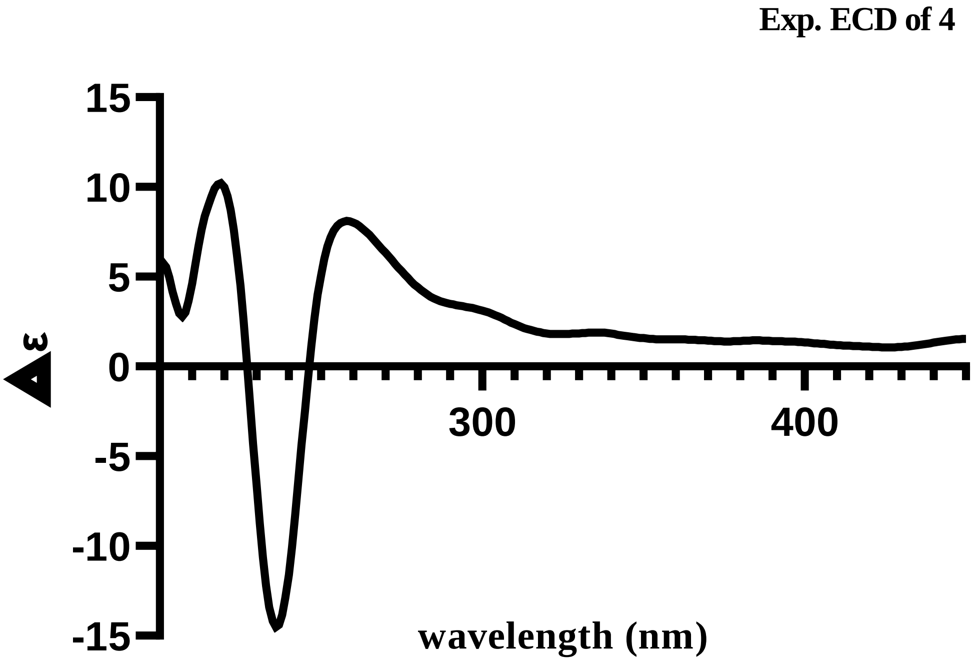

Figure S43. ECD curve for compound **4**

Computation section

Conformational searches were executed using the iMTD-GC method embeded in Crest program[1]. Density functional theory calculations were performed with the Gaussian 16 package[2]. The obtained conformers within an energy window of 3 kcal/mol were optimized at B3LYP-D3BJ/6-31G(d) with IEFPCM solvent model in methanol, and frequency analysis of all optimized conformations were also performed at the same level of theory to exclude the imaginary frequencies. ECD spectra were calculated by the TDDFT methodology at the PBE1PBE/def2TZVP utilizing IEFPCM in methanol. The final ECD spectra were simulated by averaging the spectra of lowest energy conformers according to the Boltzmann distribution theory and their relative Gibbs free energy (ΔG) using SpecDis 1.71[3] with σ = 0.40 eV and uv shift = 5 nm.

1. Pracht, P. Bohle, F. Grimme, S. Automated exploration of the low-energy chemical space with fast quantum chemical methods, Phys. Chem. Chem. Phys. 2020, 22,

7169–7192.
2. Frisch, M. J. Trucks, G. W. Schlegel, H. B. Scuseria, G. E. Robb, M. A. Cheeseman, J. R. Scalmani, G. Barone, V. Petersson, G. A. Nakatsuji, H. Li, X. Caricato, M. Marenich, A. V. Bloino, J. Janesko, B. G. Gomperts, R. Mennucci, B. Hratchian, H. P. Ortiz, J. V. Izmaylov, A. F. Sonnenberg, J. L. Williams-Young, D. Ding, F. Lipparini, F. Egidi, F. Goings, J. Peng, B. Petrone, A. Henderson, T. Ranasinghe, D. Zakrzewski, V. G. Gao, J. Rega, N. Zheng, G. Liang, W. Hada, M. Ehara, M. Toyota, K. Fukuda, R. Hasegawa, J. Ishida, M. Nakajima, T. Honda, Y. Kitao, O. Nakai, H. Vreven, T. Throssell, K. Montgomery, Jr., J. A. Peralta, J. E. Ogliaro, F. Bearpark, M. J. Heyd, J. J. Brothers, E. N. Kudin, K. N. Staroverov, V. N. Keith, T. A. Kobayashi, R. Normand, J. Raghavachari, K. Rendell, A. P. Burant, J. C. Iyengar, S. S. Tomasi, J. Cossi, M. Millam, J. M. Klene, M. Adamo, C. Cammi, R. Ochterski, J. W. Martin, R. L. Morokuma, K. Farkas, O. Foresman, J. B. and Fox D. J. Gaussian 16, Revision C.01, Gaussian, Inc., Wallingford CT, 2019.
3. Bruhn, T.; Schaumloffel, A.; Hemberger, Y.; Bringmann, G. Chirality 2013, 25, 243.

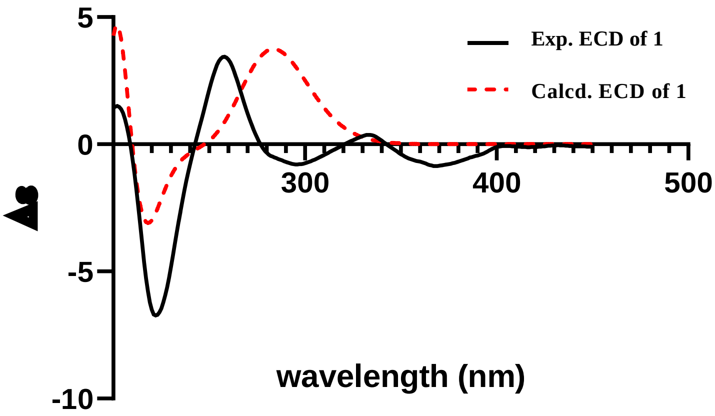

Figure S44. Experimental (black bold) spectrum of **1** in methanol and calculated ECD spectra of **1** (red dash).

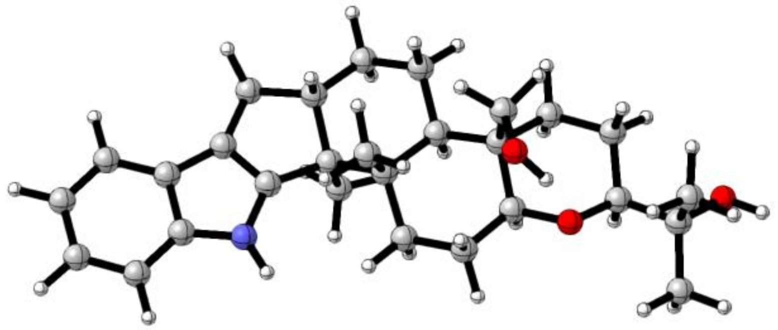

Figure S45. Optimized geometries of **1** at B3LYP-D3BJ/6-31G(d) level in methanol.

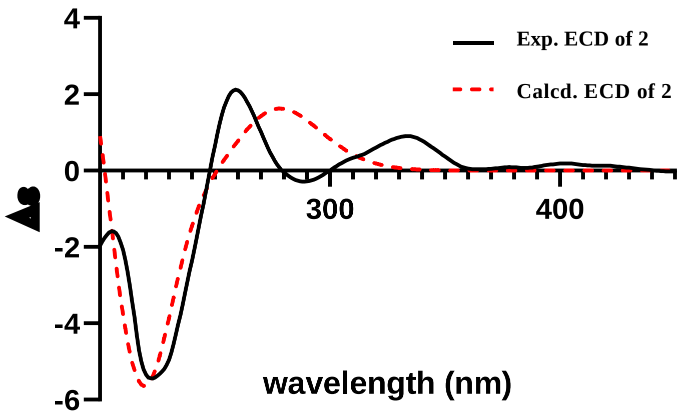

Figure S46. Experimental (black bold) spectrum of **2** in methanol and calculated ECD spectra of **2** (red dash).

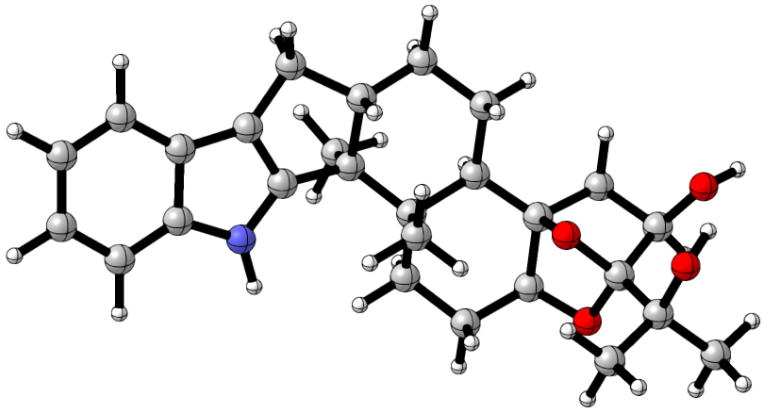

Figure S47. Optimized geometries of **1** at B3LYP-D3BJ/6-31G(d) level in methanol.

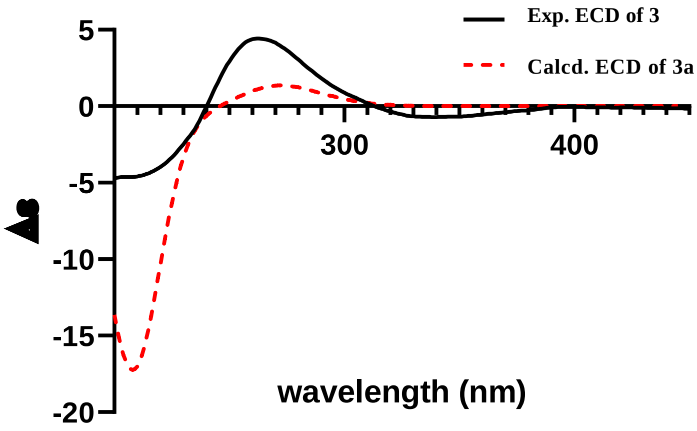

Figure S48. Experimental (black bold) spectrum of **3** in methanol and calculated ECD spectra of simplified structure (**3a**) of **3** (red dash).

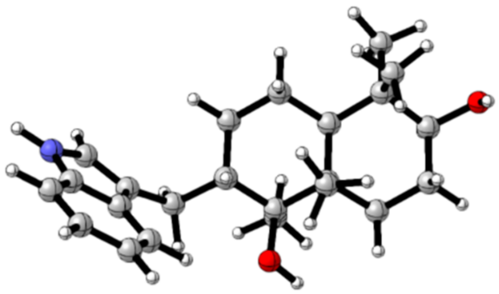

3-1

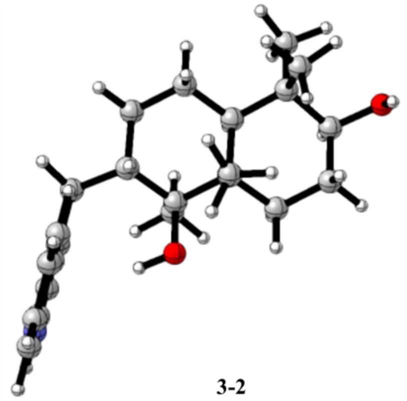

3-2

Figure S49. Optimized geometries of isomers of simplified structure (**3a**) of **3** at B3LYP-D3BJ/6-31G(d) level in methanol.

Table S1. Conformational analysis of the optimized isomers **1** at B3LYP/6-311G(d) level in chloroform.

Conformations	*G* (hartree)	*ΔG* (kcal/mol)	Boltzmann distributions (%)
**3-1**	-691306.8071	0	54.5
**3-2**	-691306.4925	0.314566033	45.5
